# MAIT cells launch a rapid, robust and distinct hyperinflammatory response to bacterial superantigens and quickly acquire an anergic phenotype that impedes their cognate antimicrobial function: Defining a novel mechanism of superantigen-induced immunopathology and immunosuppression

**DOI:** 10.1371/journal.pbio.2001930

**Published:** 2017-06-20

**Authors:** Christopher R. Shaler, Joshua Choi, Patrick T. Rudak, Arash Memarnejadian, Peter A. Szabo, Mauro E. Tun-Abraham, Jamie Rossjohn, Alexandra J. Corbett, James McCluskey, John K. McCormick, Olivier Lantz, Roberto Hernandez-Alejandro, S.M. Mansour Haeryfar

**Affiliations:** 1 Department of Microbiology and Immunology, Western University, London, Ontario, Canada; 2 Division of General Surgery, Department of Surgery, Western University, London, Ontario, Canada; 3 Department of Biochemistry and Molecular Biology, Monash University, Clayton, Victoria, Australia; 4 Biomedicine Discovery Institute, Monash University, Clayton, Victoria, Australia; 5 Institute of Infection and Immunity, Cardiff University School of Medicine, Cardiff, United Kingdom; 6 Department of Microbiology and Immunology, Peter Doherty Institute for Infection and Immunity, University of Melbourne, Parkville, Victoria, Australia; 7 Centre for Human Immunology, Western University, London, Ontario, Canada; 8 Lawson Health Research Institute, London, Ontario, Canada; 9 Laboratoire d'Immunologie and INSERM U932, Institut Curie, Paris, France; 10 Division of Transplantation, Department of Surgery, University of Rochester Medical Center, Rochester, New York, United States of America; 11 Division of Clinical Immunology and Allergy, Department of Medicine, Western University, London, Ontario, Canada

## Abstract

Superantigens (SAgs) are potent exotoxins secreted by *Staphylococcus aureus* and *Streptococcus pyogenes*. They target a large fraction of T cell pools to set in motion a “cytokine storm” with severe and sometimes life-threatening consequences typically encountered in toxic shock syndrome (TSS). Given the rapidity with which TSS develops, designing timely and truly targeted therapies for this syndrome requires identification of key mediators of the cytokine storm’s initial wave. Equally important, early host responses to SAgs can be accompanied or followed by a state of immunosuppression, which in turn jeopardizes the host’s ability to combat and clear infections. Unlike in mouse models, the mechanisms underlying SAg-associated immunosuppression in humans are ill-defined. In this work, we have identified a population of innate-like T cells, called mucosa-associated invariant T (MAIT) cells, as the most powerful source of pro-inflammatory cytokines after exposure to SAgs. We have utilized primary human peripheral blood and hepatic mononuclear cells, mouse MAIT hybridoma lines, HLA-DR4-transgenic mice, MAIT^high^HLA-DR4^+^ bone marrow chimeras, and humanized NOD-*scid* IL-2Rγ^null^ mice to demonstrate for the first time that: **i)** mouse and human MAIT cells are hyperresponsive to SAgs, typified by staphylococcal enterotoxin B (SEB); **ii)** the human MAIT cell response to SEB is rapid and far greater in magnitude than that launched by unfractionated conventional T, invariant natural killer T (*i*NKT) or γδ T cells, and is characterized by production of interferon (IFN)-γ, tumor necrosis factor (TNF)-α and interleukin (IL)-2, but not IL-17A; **iii)** high-affinity MHC class II interaction with SAgs, but not MHC-related protein 1 (MR1) participation, is required for MAIT cell activation; **iv)** MAIT cell responses to SEB can occur in a T cell receptor (TCR) Vβ–specific manner but are largely contributed by IL-12 and IL-18; **v)** as MAIT cells are primed by SAgs, they also begin to develop a molecular signature consistent with exhaustion and failure to participate in antimicrobial defense. Accordingly, they upregulate lymphocyte-activation gene 3 (LAG-3), T cell immunoglobulin and mucin-3 (TIM-3), and/or programmed cell death-1 (PD-1), and acquire an anergic phenotype that interferes with their cognate function against *Klebsiella pneumoniae* and *Escherichia coli*; **vi)** MAIT cell hyperactivation and anergy co-utilize a signaling pathway that is governed by p38 and MEK1/2. Collectively, our findings demonstrate a pathogenic, rather than protective, role for MAIT cells during infection. Furthermore, we propose a novel mechanism of SAg-associated immunosuppression in humans. MAIT cells may therefore provide an attractive therapeutic target for the management of both early and late phases of severe SAg-mediated illnesses.

## Author summary

Superantigens (SAgs) are toxins produced by *Staphylococcus aureus* and *Streptococcus pyogenes*, microbes that are responsible for a multitude of infectious diseases and conditions. Once released, SAgs activate many immune cells, resulting in a massive inflammatory response that is often followed by a state of immunosuppression, a state that favors opportunistic infections. Using primary human cells as well as wild-type and genetically altered mice, we have now identified a subset of unconventional, innate-like T lymphocytes, called mucosa-associated invariant T (MAIT) cells, as one of the most powerful and quick-acting sources of inflammatory mediators in the aftermath of systemic exposure to SAgs. We also demonstrate that robust activation of MAIT cells by SAgs quickly leads to their exhaustion, and this exhaustion interferes with their ability to participate in antimicrobial host defense and contributes to the immunosuppressive state. Our findings thus define a pathogenic role for MAIT cells during Gram-positive bacterial infections and also uncover a novel mechanism of SAg-mediated immunosuppression. Accordingly, we propose that MAIT cells can be targeted for efficacious treatment of SAg-mediated illnesses.

## Introduction

Bacterial exotoxins known as superantigens (SAgs) constitute a family of virulence factors deployed by common bacterial pathogens such as *S*. *aureus* and *S*. *pyogenes* [1]. SAgs cause a variety of illnesses, including but not limited to food poisoning, scarlet fever, and menstrual and non-menstrual toxic shock syndrome (TSS). Certain SAg-mediated illnesses inflict severe morbidity or even death and are, as such, considered serious clinical emergencies [2]. Also, alarmingly, SAgs can be weaponized and used against civilian populations. As a matter of fact, staphylococcal enterotoxin B (SEB), a major cause of non-menstrual TSS, is listed by the Centers for Disease Control and Prevention among “category B priority” bioterrorism agents [3].

As intact and unprocessed proteins, SAgs bind to lateral surfaces of MHC class II molecules found on antigen (Ag)-presenting cells [4] and to T cell receptor (TCR) Vβ regions of many T cells [5]. These unorthodox interactions short-circuit the normal sequence of events that typically activates only a tiny proportion of T cells with unique TCR specificities for cognate peptide:MHC complexes, which is approximately 1 in every 10,000 T cells. By defying the rule of MHC restriction, SAgs activate as many as 20% of all exposed T cells, regardless of their TCR specificity [1]. This, in turn, leads to a massive “cytokine storm” and hyperinflammation and, under certain circumstances, to organ failure. In addition, in vivo exposure to SAgs punches “holes” in the T cell repertoire by deleting many T cells [5], while other SAg-responsive T cells may undergo anergy [6]. Consequently, a fraction of pathogen-specific T cells are physically or functionally removed from action in the battle against microbes, hypothetically including the very bacteria that produce SAgs. Of note, SAg-induced T cell deletion and anergy have been extensively studied in mouse models. Whether human conventional T (T_conv_) cells or innate-like T cells are similarly affected by SAgs remains poorly understood.

CD4^+^ and CD8^+^ T_conv_ cells are known targets of SAgs. In contrast, in what capacity non-MHC-restricted T cells may participate in SAg-mediated immunopathology is far from clear. γδ T cells have been implicated in host responses to SAgs [7,8]. We and others also reported that invariant natural killer T (*i*NKT) cells can be directly activated by group II bacterial SAgs in a CD1d-independent fashion [9,10]. However, human *i*NKT cells are infrequent, especially in comparison with mucosa-associated invariant T (MAIT) cells that comprise 1%–10% of all peripheral blood T cells, up to 10% of intestinal T cells, and approximately 45% of all hepatic lymphocytes in humans [11,12].

MAIT cells are innate-like T lymphocytes that express an invariant TCRα (*i*TCRα) chain with a unique Vα19-Jα33 rearrangement in mice and Vα7.2-Jα33 in humans [13,14]. They are restricted by MHC-related protein 1 (MR1) [15], a monomorphic MHC class I—like molecule that is highly conserved among mammalian species [16] and presents microbe-derived vitamin B metabolites [17]. These discoveries underpinned the recent invention of MR1 tetramer reagents that enable MAIT cell identification [18,19]. Human MAIT cells are also phenotyped as CD3^+^Vα7.2^+^CD161^high^ cells.

MAIT cells can be viewed as “emergency responders” to infection. This is because: **i)** they occupy strategic locations at the host—pathogen interface; **ii)** they quickly amass at infection sites where they respond to a variety of bacteria and fungi [20–22]; **iii)** they exhibit an effector memory-like phenotype [11] and are capable of producing pro- and/or anti-inflammatory cytokines (e.g., IFN-γ, TNF-α, IL-4, IL-10) readily, amply and promptly after *i*TCR stimulation. The nature of cytokines released by MAIT cells is likely to influence the function(s) of various downstream effector cell types. This, in turn, either promotes immunity to or immunopathology caused by microbial intruders. It is noteworthy that MAIT cells can also be activated by a combination of IL-12 and IL-18 [23], which are released during many infections.

Despite all the above attributes, it is unclear whether MAIT cells respond to bacterial SAgs. This is an important question in light of the enormous immunomodulatory properties of these cells. MAIT cells are enriched in the intestine and in the human liver, which receive continuous, heavy exposure to microbes, including SAg-producing bacteria. Equally important, cells expressing “SAg-responsive” Vβs are present within the MAIT cell *i*TCR repertoire [24].

In this work, we have utilized MAIT hybridoma cell lines, wild-type mice, HLA-DR4-transgenic (DR4 tg) mice, MAIT cell-enriched bone marrow chimeric mice, and humanized NOD-*scid* IL-2Rγ^null^ (NSG) mice, as well as human peripheral blood mononuclear cells (PBMCs) and non-parenchymal hepatic mononuclear cells (HMNCs) to investigate the responsiveness of MAIT cells to a wide panel of bacterial SAgs in vitro and/or in vivo. We report for the first time, to our knowledge, that select staphylococcal and streptococcal SAgs trigger rapid activation of MAIT cells in an MR1-independent manner. In addition, MAIT cell activation by SAgs occurs through *i*TCR triggering and/or IL-12/IL-18 signaling. Interestingly, the responses launched by human MAIT cells were far greater in magnitude than those elicited by T_conv_ cells, *i*NKT cells, or γδ T cells. However, the generation of multifunctional, hyperinflammatory MAIT cells by SAgs was followed by a state of anergy that hampered their cognate response to bacterial pathogens. We propose a novel mechanism of immunosuppression in the aftermath of exposure to bacterial SAgs, which involves a distinct subset of unconventional, innate-like human T cells.

## Results

### Human MAIT cells are the most potent early producers of IFN-γ in response to SEB

**Fig 1:**
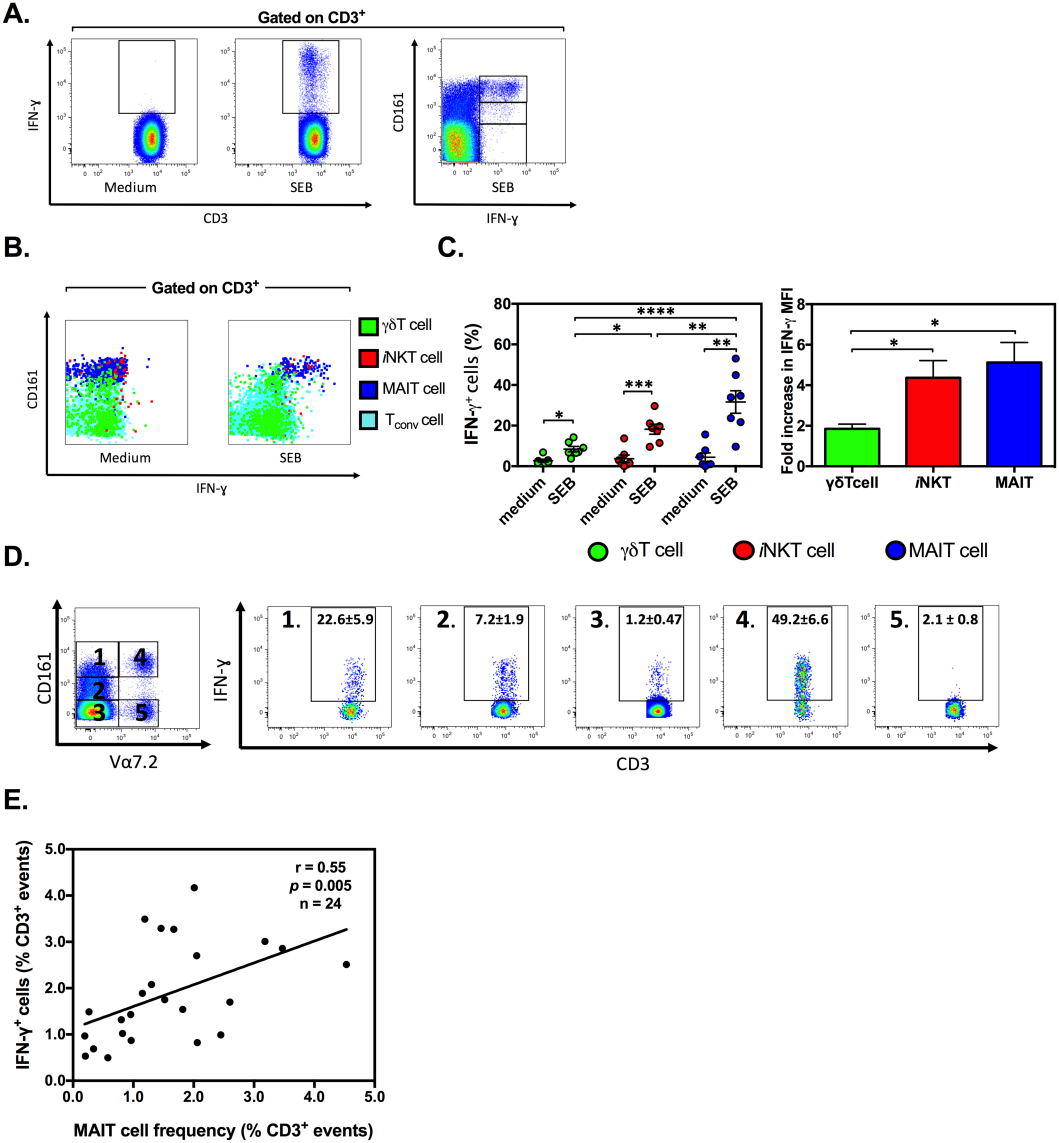
Human peripheral blood mucosa-associated invariant T (MAIT) cells are a predominant source of interferon (IFN)-γ after exposure to staphylococcal enterotoxin B (SEB). Human peripheral blood mononuclear cells (PBMCs) were left untreated or stimulated for 24 h with 100 ng/mL of SEB. Intracellular IFN-γ was detected by flow cytometry among bulk CD3^+^, CD3^+^CD161^-^, CD3^+^CD161^low^, and CD3^+^CD161^high^ cells (**A**). PBMCs were additionally stained with monoclonal antibodies (mAbs) to T cell receptor (TCR) γδ and TCR Vα7.2 and with PBS-57-loaded CD1d tetramer. Events corresponding to γδ T (green), invariant natural killer T (*i*NKT) (red), MAIT (blue), and conventional T (T_conv_) (cyan) cells were superimposed to generate a dot plot (**B**). The frequency of IFN-γ^+^ cells and the mean fluorescence intensity (MFI) of IFN-γ staining were determined in 7 donors, each of whom is represented by a circle (**C**). Five subpopulations were defined among SEB-stimulated CD3^+^ cells based on their co-expression of CD161 and Vα7.2, or lack thereof. The proportion of IFN-γ^+^ cells for each subpopulation is demonstrated in representative FACS plots (**D**). Historical data from 24 donors were subjected to Spearman’s rank correlation analysis to test the association between MAIT cell and IFN-γ^+^ cell frequencies (**Fig 1E**). Mean ± SEM values are shown in **C** (*n* = 7) and **D** (*n* = 9). *, **, ***, and **** in panel C denote statistical differences with *p* < 0.05, *p* < 0.01, *p* < 0.001, and *p* < 0.0001, respectively. The underlying data for this figure can be found in S1 Data, and our gating strategies are provided in S2 Data.

While T_conv_ cells are considered the main targets of bacterial SAgs, the effector functions of “innate-like” T cells following their exposure to SAgs have been largely overlooked, due perhaps to their lower frequencies in the circulation. When investigating the relative contribution of various human T cell subsets to SEB-triggered production of IFN-γ, a pro-inflammatory cytokine that is key to the pathogenesis of SAg-mediated illnesses, we found the vast majority of IFN-γ-secreting CD3^+^ T cells to strongly express CD161 (Fig 1A).

CD161 is a C-type lectin that is more abundantly expressed by innate-like γδ T, *i*NKT, and MAIT cells than by T_conv_ cells [25]. Therefore, we evaluated the contribution of these cell types to IFN-γ production. Our experiments revealed that MAIT cells are the major source of this cytokine after stimulation with SEB (Fig 1B and 1C). The frequency of *i*NKT and γδ T cells with detectable intracellular IFN-γ was increased upon SEB stimulation (Fig 1C). However, MAIT cells were clearly the predominant IFN-γ^+^ population (Fig 1B and 1C). Of note, MAIT and *i*NKT cells synthesized more IFN-γ on a per cell basis, as judged by their mean fluorescence intensity (MFI) of IFN-γ expression, in comparison with γδ T cells (Fig 1C). Importantly, when compared with other CD3^+^ T cell fractions, MAIT cells elicited a more robust IFN-γ response to SEB (Fig 1D). Finally, using data from a relatively large cohort of blood donors, we found a positive correlation between MAIT cell and IFN-γ^+^ cell frequencies following SEB stimulation of PBMCs (Fig 1E).

It is important to note that in control experiments, we have ruled out the possibility of a role for endotoxin contamination in MAIT cell activation. We demonstrated that adding lipopolysaccharide (LPS) to PBMC cultures does not increase SEB-induced IFN-γ production by MAIT cells (S1A Fig). Furthermore, adding polymyxin B to PBMC cultures before SEB stimulation did not lower the response to SEB (S1A Fig). To make sure polymyxin B was effective in blocking the action of LPS in our hands, we stimulated the human monocytic cell line THP-1 with LPS in the presence or absence of this antibiotic. As expected, treatment with polymyxin B dramatically reduced LPS-induced TNF-α production by THP-1 cells (S1B Fig).

**Fig 2:**
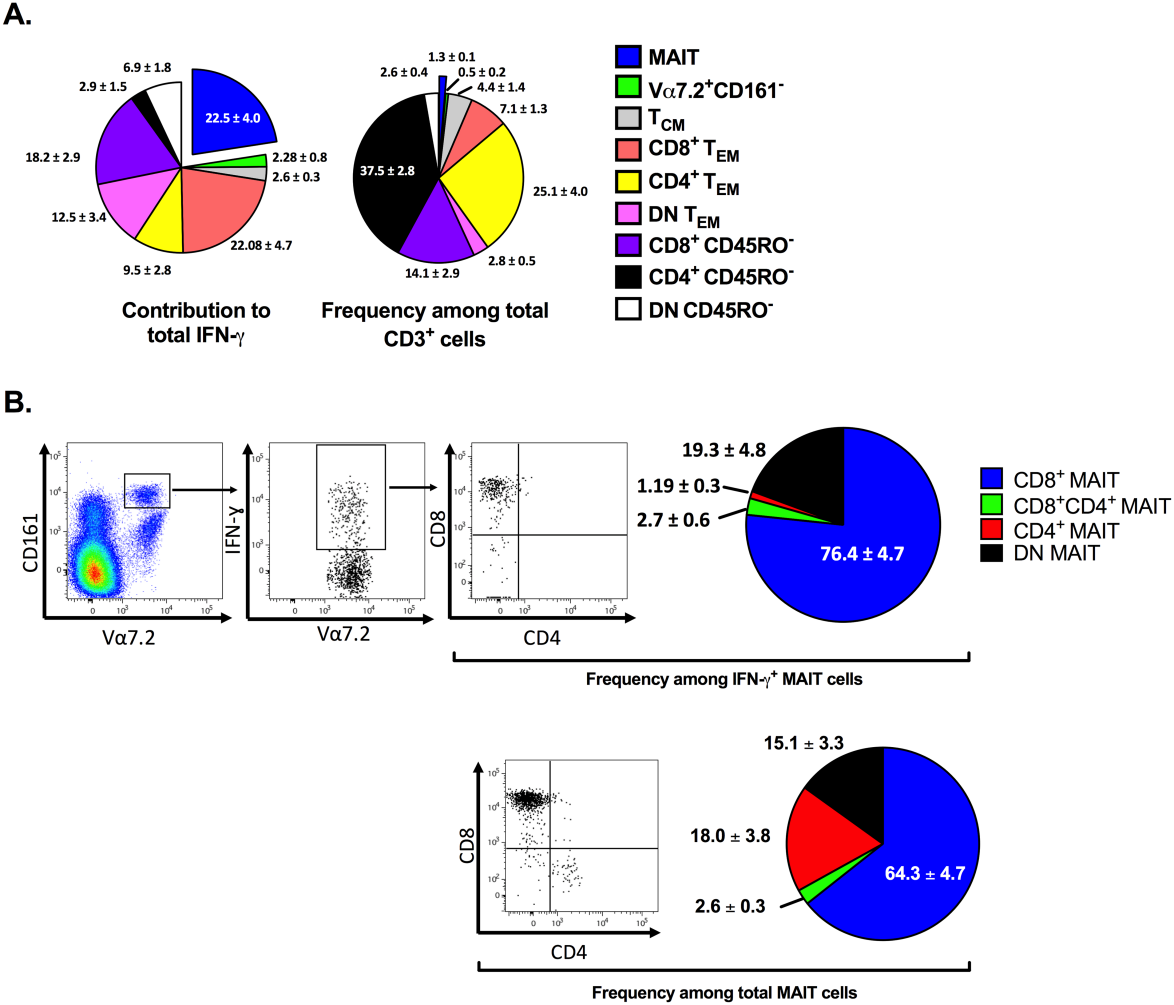
Human CD8^+^ mucosa-associated invariant T (MAIT) cells mount a more intense interferon (IFN)-γ response to staphylococcal enterotoxin B (SEB) than do memory conventional T (T_conv_) cells at the individual cell level. Human peripheral blood mononuclear cells (PBMCs) (*n* = 7) were exposed to SEB for 24 h. IFN-γ-producing T cells were then immunophenotyped by flow cytometry to determine the percentages of Vα7.2^+^CD161^high^ (MAIT) cells, Vα7.2^+^CD161^-^ cells, CD45RO^+^CCR7^+^Vα7.2^-^ central memory T (T_CM_) cells, the CD4^+^, CD8^+^, or double negative (DN) subsets of CD45RO^+^CCR7^-^Vα7.2^-^ effector memory T (T_EM_) cells, and the CD4^+^, CD8^+^, or DN subsets of CD45RO^-^ cells (**A**). The frequencies of bulk and IFN-γ^+^ MAIT cells expressing CD4 and/or CD8 were also calculated and presented in a pie chart (*n* = 8) (**B**). The underlying data for this figure can be found in S1 Data, and our gating strategies are provided in S2 Data.

Given that MAIT cells have an effector memory-like phenotype [11], we next made comprehensive, head-to-head comparisons between MAIT cells and effector memory T_conv_ cells among other T cell subsets in a separate cohort (*n* = 7). We found that CD3^+^CD8^+^CD45RO^+^CCR7^-^Vα7.2^-^ effector memory T cells (T_EM_) and CD3^+^Vα7.2^+^CD161^high^ MAIT cells make significant contributions to the overall IFN-γ response (Fig 2A).

The contribution of MAIT cells was particularly impressive given their lower frequency. To be exact, MAIT cells were responsible for 22.5% of total IFN-γ production, despite their average frequency of 1.3%. In contrast, CD8^+^ and CD4^+^ T_EM_ comprised 7.1% and 25.1% of all CD3^+^ cells and could account for 22.1% and 9.5% of the IFN-γ response, respectively (Fig 2A). We verified the above results by MR1 tetramer staining. Accordingly, MR1 tetramer^+^ MAIT cells constituted only 1.3% (± 0.5%) of CD3^+^ PBMCs, but 17.4% (± 5.2%) of all CD3^+^IFN-γ^+^ cells.

MAIT cells can be divided into several subsets based on the type of co-receptor(s) they express, and it was of interest to determine whether treatment with SEB affects the subset distribution of MAIT cells. We found the majority of SEB-exposed blood MAIT cells to be CD8^+^, which is similar to their resting, steady state [18]. In addition, most IFN-γ-producing MAIT cells fell within the CD8^+^ subset (Fig 2B).

**Fig 3:**
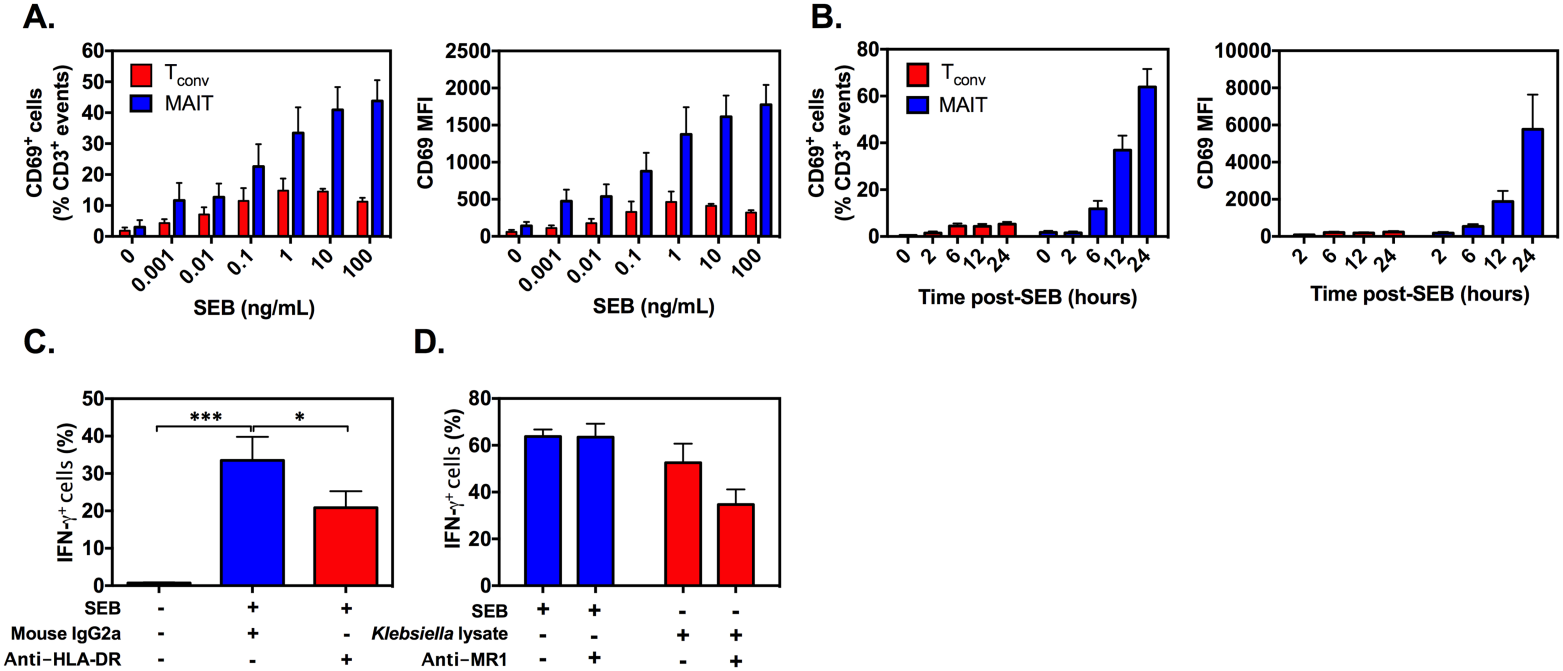
Rapid, exaggerated mucosa-associated invariant T (MAIT) cell responses to staphylococcal enterotoxin B (SEB) require the presence of HLA class II but not MHC-related protein 1 (MR1). Human peripheral blood mononuclear cells (PBMCs) were stimulated for 12 h with indicated doses of SEB (**A**) or for indicated durations with 100 ng/mL of SEB (**B**) before the frequency of CD69^+^ events and the mean fluorescence intensity (MFI) of CD69 were determined among MAIT and conventional T (T_conv_) cells. Data were pooled from at least 3 independent experiments, and mean ± SEM values are shown (*n* = 5 for A and *n* = 8 for B). PBMCs (*n* = 9 in C; *n* = 3 in D) were incubated for 24 h with SEB (**C-D**) or *Klebsiella* lysate (**D**) in the presence of an HLA-DR-blocking monoclonal antibody (mAb) or an isotype control (**C**) or an anti-MR1 mAb (**D**). The percentage of interferon (IFN)-γ^+^ events among MAIT cells is reported, and error bars represent SEM. The underlying data for this figure can be found in S1 Data, and our gating strategies are provided in S2 Data.

Next, we demonstrated that SEB-induced activation of both MAIT and T_conv_ cells follows a dose-dependent pattern and reaches its plateau at around 1 ng/mL of SEB (Fig 3A).

The readout in these experiments was the expression of CD69, an early activation marker, which also allowed us to compare the kinetics of MAIT and T_conv_ cell responses to SEB. Both cell types exhibited readily detectable CD69 expression as early as 6 h post-SEB exposure (Fig 3B). However, the magnitude of MAIT cell activation in bulk cultures was far greater than that of T_conv_ cells at multiple time points (6 h, 12 h, and 24 h), thus recapitulating our intracellular IFN-γ results.

In the next series of experiments, we asked whether and to what extent human MAIT cell responses to SEB may depend on HLA class II or MR1. Unlike a mouse IgG2a isotype control, an HLA-DR-blocking monoclonal antibody (mAb), which was added to PBMC cultures before the SEB challenge, decreased the frequency of IFN-γ^+^ MAIT cells (Fig 3C). In contrast, blockade of MR1 failed to alter this response (Fig 3D). The anti-MR1 mAb used in these experiments was functional, as evidenced by its ability to attenuate MAIT cell activation by *K*. *pneumonia* lysate (Fig 3D), which was used as a crude source of MAIT cell cognate Ags [26,27].

Collectively, the results summarized in Figs 1–3 demonstrate that MAIT cells are hyperresponsive to bacterial SAgs and rapidly produce high levels of IFN-γ as a result. This response does not require MR1 participation and is more robust than those mounted by T_conv_ cells or innate-like T cell types other than MAIT cells.

### SEB-stimulated blood and hepatic MAIT cells exhibit a distinct cytokine signature

**Fig 4:**
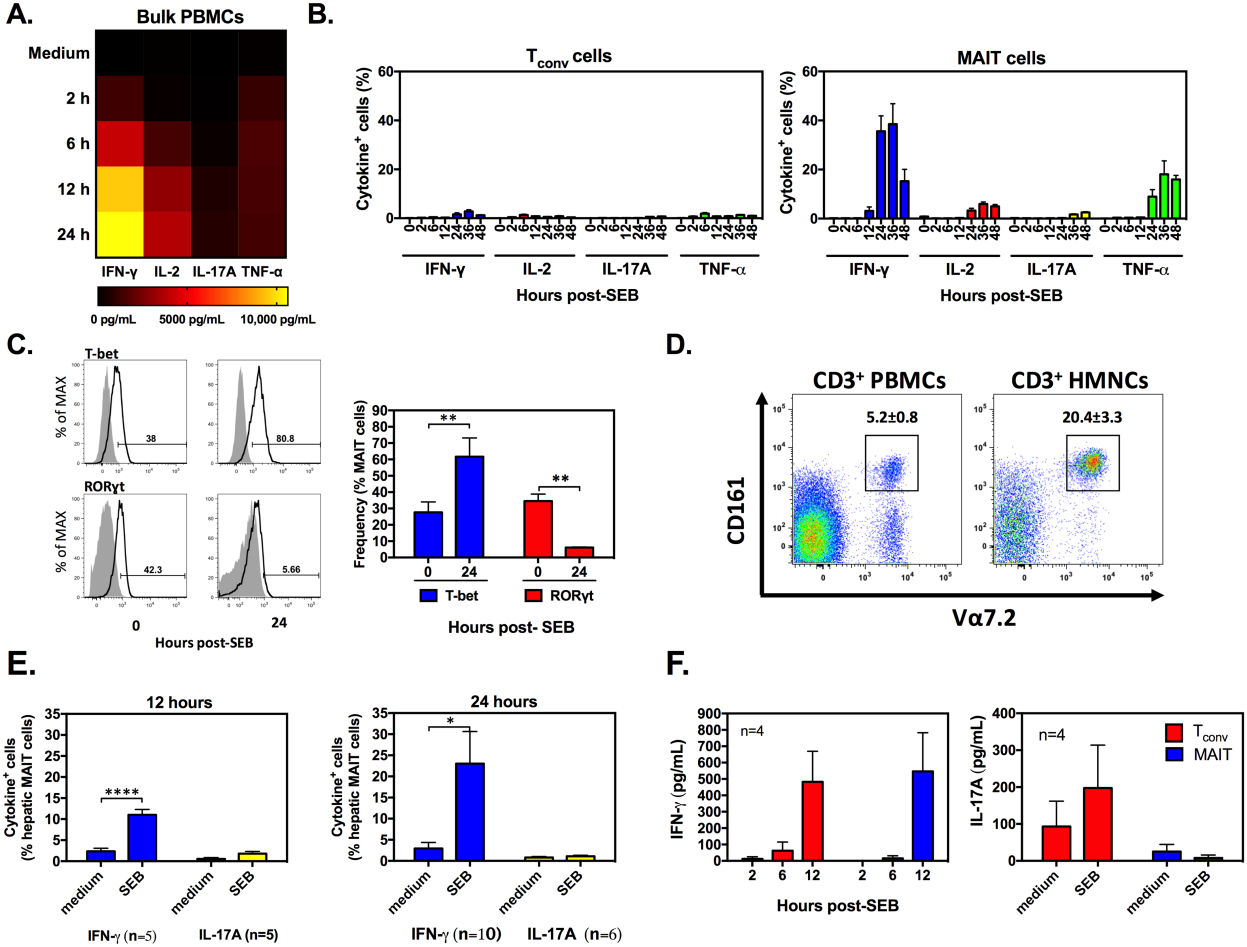
Staphylococcal enterotoxin B (SEB) stimulates human peripheral blood and hepatic mucosa-associated invariant T (MAIT) cells to produce classic pro-inflammatory cytokines except interleukin (IL)-17. Human peripheral blood mononuclear cells (PBMCs) were stimulated with 100 ng/mL of SEB, and culture supernatants were collected at indicated time points for cytokine analysis (**A**). Data from 4 healthy donors were averaged and used to generate a heat map illustrating interferon (IFN)-γ, interleukin (IL)-2, IL-17A, and tumor necrosis factor (TNF)-α levels. PBMCs (*n* = 8) were exposed to SEB, and the frequencies of IFN-γ^+^, IL-2^+^, TNF-α^+^, and IL-17^+^ events among conventional T (T_conv_) and MAIT cells were determined at indicated time points (**B**). Freshly isolated and SEB-stimulated PBMCs (*n* = 3) were also examined to assess the intracellular T-bet and RORγT contents of MAIT cells relative to background staining with isotype controls (filled histograms in representative plots) (**C**). Non-parenchymal hepatic mononuclear cells (HMNCs) were isolated from tumor-free liver tissue samples of patients with colorectal carcinoma (*n* = 20), in which CD3^+^Vα7.2^+^CD161^+^ MAIT cell frequencies were calculated and compared with those determined in 20 PBMC samples (**D**). HMNCs were incubated for 12 h or 24 h with SEB, followed by cytofluorimetric analysis of IFN-γ and IL-17 production by hepatic MAIT cells (**E**). In a limited number of experiments, blood MAIT and T_conv_ cells were purified using a cell sorter and co-incubated with autologous CD14^+^ monocytes in the presence or absence of SEB. IFN-γ and IL-17A contents of culture supernatants were measured after 2 h, 6 h, or 12 h by ELISA (**F**). *, **, and **** denote *p* < 0.05, *p* < 0.01, and *p* < 0.0001, respectively. The underlying data for this figure can be found in S1 Data, and our gating strategies are provided in S2 Data.

Exposure to SEB triggers the release of multiple inflammatory cytokines from multiple cell types. To begin to address the role of MAIT and T_conv_ cells in pro-inflammatory cytokine production, we first performed cytokine multiplexing on PBMC culture supernatants harvested at 2 h, 6 h, 12 h, and 24 h post-SEB stimulation (Fig 4A). We found a gradual increase in the IFN-γ, IL-2, IL-17A, and TNF-α content of these samples.

Since these cytokines can be of T_conv_ and MAIT cell origin, we set out to examine the relative contribution of these cells to cytokine responses. IFN-γ^+^, IL-2^+^, and TNF-α^+^ MAIT cells accumulated gradually and dramatically within SEB-stimulated PBMC cultures, thus yielding frequencies that far exceeded those of T_conv_ cells (Fig 4B). To our surprise, however, IL-17A^+^ MAIT cells were barely detectable (Fig 4B). Consistent with this observation, while the expression of T-bet, a transcription factor linked to a Th1 phenotype and IFN-γ production, doubled in SEB-exposed MAIT cells, the intracellular levels of RORγt, a master regulator of Th17-type responses, decreased, rather than increased, after SEB stimulation (Fig 4C).

The inflammatory cytokine profiles of MAIT cells can be impacted by the anatomical location of these cells and by the experimental conditions and stimuli employed for their activation [11,12,28]. Therefore, we sought to examine how exposure to SEB affects the production of pro-inflammatory cytokines by liver-resident MAIT cells. We chose to work with HMNCs because the liver accommodates a large number of MAIT cells [11,12]. To ascertain whether hepatic MAIT cells behave similarly in response to SEB, we first confirmed that tumor-free liver tissue samples obtained from colorectal carcinoma patients contained many CD3^+^Vα7.2^+^CD161^+^ MAIT cells (Fig 4D). HMNCs were isolated and exposed to SEB for 12 h or 24 h before IFN-γ^+^ and IL-17A^+^ MAIT cells were enumerated. Similar to their blood counterparts, hepatic MAIT cells launched a strong IFN-γ response and only a negligible IL-17A response to SEB (Fig 4E).

Next, we extended our investigation to assess the cytokine secretion capacity of purified MAIT cells. Sorted CD3^+^Vα7.2^+^CD161^+^ cells were co-incubated for 2 h, 6 h, and/or 12 h with autologous CD14^+^ monocytes, as accessory cells, in the presence or absence of SEB (Fig 4F). As with bulk PBMC cultures, SEB stimulation of purified MAIT cells led to substantial IFN-γ production but no IL-17A secretion.

Taken together, these results indicate that: **i)** peripheral blood and hepatic MAIT cells respond similarly to SEB; **ii)** MAIT cell activation by SAgs results in selective, as opposed to global, pro-inflammatory cytokine release, with the notable and surprising absence of an IL-17A component.

### Mouse and human MAIT cell activation by bacterial SAgs can be TCR Vβ–specific

**Fig 5:**
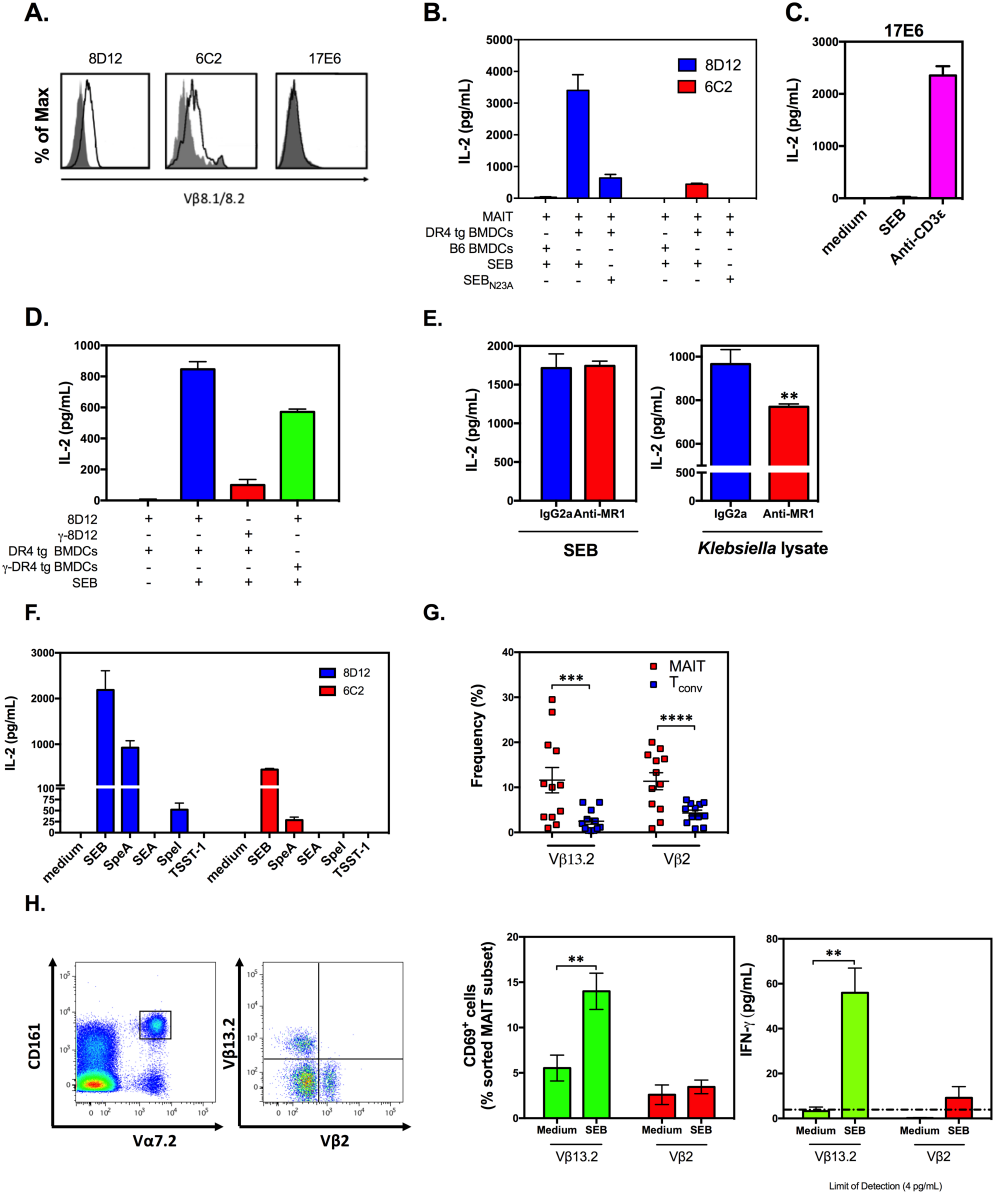
Mouse and human mucosa-associated invariant T (MAIT) cells can be activated by staphylococcal and streptococcal superantigens (SAgs) in a T cell receptor (TCR) Vβ-dependent manner. Mouse MAIT hybridoma lines 8D12, 6C2, and 17E6 were evaluated by flow cytometry for TCR Vβ8.1/2 expression (**A**). Indicated hybridoma(s) were exposed to 100 ng/mL of staphylococcal enterotoxin B (SEB) (**B-F**), SEB_N23A_ (**B**), or several SAgs other than SEB (**F**) or stimulated with 0.5 μg/mL of an anti-CD3ε mAb (**C**) or with *Klebsiella* lysate (**E**) in the presence of DR4-transgenic (DR4 tg) bone marrow-derived dendritic cells (BMDCs) (**B-F**), wild-type C57BL/6 (B6) BMDCs (**B**), or γ-irradiated DR4 tg BMDCs (**D**). In several experiments, 5 μg/mL of an MHC-related protein 1 (MR1)-blocking monoclonal antibody (mAb) or an IgG2a isotype control was added to 8D12 cultures prior to stimulation with SEB or *Klebsiella* lysate (**E**). Culture supernatants were collected after 24 h, and interleukin (IL)-2 levels were quantified by ELISA. Representative data from 3 to 4 independent experiments yielding similar results are illustrated in panels A-F. Error bars represent SD to demonstrate variation among technical replicates. The frequencies of TCR Vβ13.2^+^ and Vβ2^+^ MAIT and conventional T (T_conv_) cell subsets were determined among human peripheral blood mononuclear cells (PBMCs) isolated from 12 donors (**G**). To purify the above MAIT cell fractions, CD3^+^Vα7.2^+^CD161^+^ PBMCs were co-stained with monoclonal antibodies (mAbs) to TCR Vβ13.2 and Vβ2. MAIT cell subsets were then sorted and co-incubated with autologous CD14^+^ monocytes in the absence or presence of SEB (*n* = 3 to 4). Twenty-four hours later, CD69^+^ cell percentages and interferon (IFN)-γ levels in culture supernatants were determined (**H**). Error bars in panels G and H represent SEM. **, ***, and **** indicate *p* < 0.01, *p* < 0.001, and *p* < 0.0001, respectively. The underlying data for this figure can be found in S1 Data, and our gating strategies are provided in S2 Data.

To determine whether MAIT cells expressing “SEB-responsive” TCR Vβ chains are directly activated by this SAg, we took advantage of several well-characterized mouse MAIT hybridomas, namely lines 8D12, 6C2, and 17E6 [14,16]. 8D12 and 6C2 cells express Vβ8 (Fig 5A), a known target of SEB in mice, whereas 17E6 is a Vβ2^+^ hybridoma [14] that should not respond to SEB.

When stimulated with SEB in the presence of wild-type bone marrow-derived dendritic cells (BMDCs), neither 8D12 nor 6C2 cells released IL-2 into the culture supernatant (Fig 5B). We posited that this was merely a reflection of the low affinity of mouse MHC class II molecules for SEB [29]. Indeed, when we used BMDCs generated from DR4 tg mice as accessory cells, both these hybridomas, but not 17E6, were responsive to SEB (Fig 5B and 5C). The failure of 17E6 cells to produce IL-2 was not due to defective *i*TCR expression or function because an agonistic anti-CD3ε mAb was able to trigger their activation (Fig 5C). To confirm that DR4 tg BMDCs were only physically required for mouse MAIT cell responses—that is, to mainly supply HLA DR4 and perhaps cell-surface costimulatory molecules of mouse origin—we compared γ-irradiated and nonirradiated BMDCs in 8D12 stimulation cultures. While γ irradiation of 8D12 cells nearly abrogated their ability to secrete IL-2, as expected, γ-irradiated DR4 tg BMDCs could still prompt an impeccable response that was only marginally weaker than that elicited by nonirradiated BMDCs (Fig 5D).

To definitively show that *i*TCR engagement by SEB was a prerequisite for IL-2 secretion by 8D12 and 6C2, we used SEB_N23A_, a mutated version of SEB with a partially impaired TCR binding capacity [30], in parallel cultures. As anticipated, SEB_N23A_ did not equal SEB in eliciting an IL-2 response (Fig 5B). We also demonstrated that as with human MAIT cells, mouse MAIT cell activation by SEB was MR1-independent (Fig 5E).

Finally, we extended our study to examine MAIT cell responses to a relatively wide panel of staphylococcal and streptococcal toxins belonging to multiple evolutionary groups of SAgs [1]. These included toxic shock syndrome toxin -1 (TSST-1), streptococcal pyrogenic exotoxin A (SpeA), staphylococcal enterotoxin A (SEA), and SpeI, which represent groups I, II, III, and V SAgs, respectively [1]. Interestingly, SEB and SpeA, which originate from 2 different Gram-positive pathogens but are grouped together under the same phylogenetic branch of SAgs (i.e., group II) with known reactivity to mouse Vβ8 [31], provoked MAIT cell activation (Fig 5F).

The majority of human MAIT cells have a remarkably stable TCRβ repertoire that is biased towards Vβ2 and Vβ13 families [14,18,24]. On the other hand, SEB targets human Vβ13.2 but not Vβ2. This presented a unique opportunity to explore the direct responsiveness of the respective human MAIT cell subpopulations to SEB. Using PBMCs obtained from 12 healthy donors, we first confirmed the higher frequencies of TCR Vβ13.2^+^ and TCR Vβ2^+^ fractions among MAIT cells in comparison with T_conv_ cells (Fig 5G). We then purified these fractions and stimulated them with SEB in the presence of autologous monocytes. As hypothesized, Vβ13.2^+^, but not Vβ2^+^, MAIT cells upregulated CD69 and secreted IFN-γ 12 h after they were exposed to SEB (Fig 5H). Therefore, both mouse and human MAIT cells that express “SAg-responsive” TCR Vβ families can be directly activated by bacterial SAgs.

### SEB-induced IL-12 and IL-18 production results in MAIT cell hyperactivation

**Fig 6:**
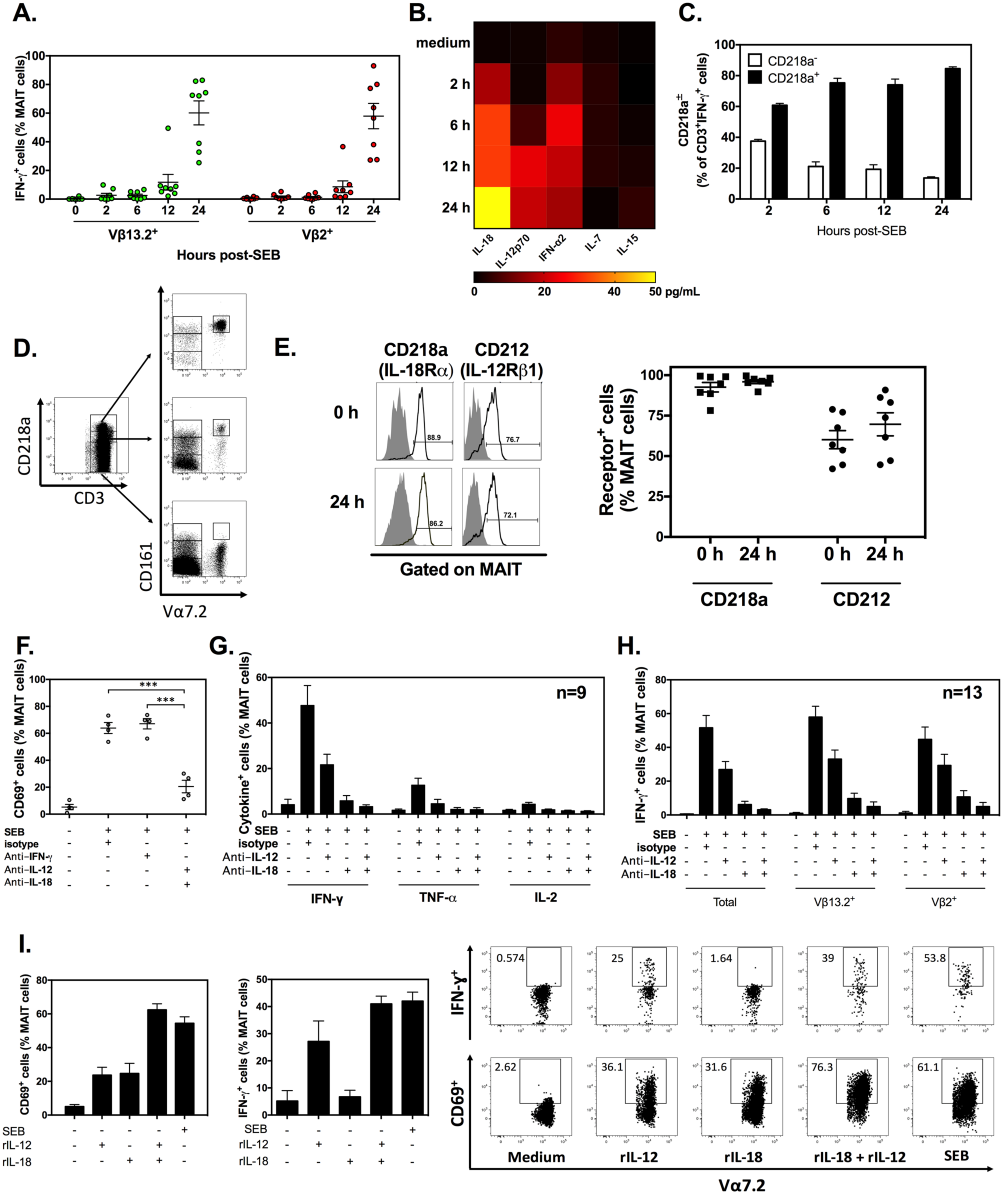
Staphylococcal enterotoxin B (SEB) can activate human mucosa-associated invariant T (MAIT) cells by an interleukin (IL)-12/IL-18-dependent mechanism. Peripheral blood mononuclear cells (PBMCs) were exposed to SEB for 24 h, and interferon (IFN)-γ^+^ events were enumerated among Vβ13.2^+^ and Vβ2^+^ MAIT cells (**A**). Human PBMCs (*n* = 4) were stimulated with SEB before culture supernatants were harvested at indicated time points to assay for IL-18, IL-12p70, IFN-α2, IL-7, and IL-15. Data were averaged to generate a heat map (**B**). The relative frequencies of CD218a^+^ and CD218a^-^ cells within the CD3^+^IFN-γ^+^ gate was determined at indicated time points post-SEB stimulation (*n* = 3) (**C**). CD3^+^ cells exhibiting high, intermediate and low surface levels of CD218a were further analyzed for CD161 and Vα7.2 positivity (**D**). CD218a and CD212 expression by MAIT cells was assessed in untreated and SEB-stimulated PBMC cultures (*n* = 7) (**E**). Filled and open histograms correspond to staining with isotype controls and anti-CD218a/CD212, respectively (**E**). In several experiments, neutralizing monoclonal antibodies (mAbs) to IFN-γ, IL-12, and/or IL-18 (or isotype control[s]) were added to PBMC cultures prior to SEB stimulation. Twenty-four hours later, the percentages of CD69^+^ (**F**) and cytokine^+^ events (**G-H**) were determined among total (**F-H**) or fractionated (**H**) MAIT cells. In additional cultures, PBMCs were stimulated for 24 h with SEB or with recombinant human IL-12 (rIL-12) and/or recombinant human IL-18 (rIL-18) in parallel before cell-surface expression of CD69 (*n* = 8) and intracellular IFN-γ accumulation (*n* = 4) in MAIT cells were evaluated (**I**). Error bars represent SEM, and *** indicates a statistically significant difference with *p* < 0.001. The underlying data for this figure can be found in S1 Data, and our gating strategies are provided in S2 Data.

Our experiments using purified MAIT cells demonstrated that *i*TCR ligation contributes to SAg-mediated responses (Fig 5). However, in vivo T cell responses occur in the presence of other cell types and amid an intricate cytokine milieu, which can be simulated in bulk cultures. To determine the impact of the microenvironment in which MAIT cells encounter SAgs, we compared Vβ13.2^+^ and Vβ2^+^ MAIT fractions in unfractionated human PBMC cultures. When exposed to SEB, Vβ2^+^ cells were capable of making IFN-γ (Fig 6A).

Intriguingly, IFN-γ synthesis by Vβ13.2^+^ and Vβ2^+^ MAIT cells was equally vigorous and followed a similar kinetics (Fig 6A). When we used the expression of the early activation marker CD69 as a readout, a similar pattern emerged, although, interestingly, the activation of Vβ2^+^ MAIT cell appeared to lag slightly behind that of the Vβ13.2^+^ subset (S2 Fig). These results suggested that a cytokine-mediated pathway was operational and fully capable of compensating for a lack of Vβ2^+^ *i*TCR cross-linking by SEB. Therefore, we considered the possibility of MAIT cell transactivation by a combination of IL-18 and IL-12 or by IFN-γ, which contribute to innate lymphocyte activation in other settings [23,32]. In fact, IL-18 is known to potentiate IFN-γ responses—hence its historical name “IFN-γ-inducing factor” [33]. Several other cytokines have also been implicated in direct or indirect activation of MAIT cells. These include IL-7 [12,34], IL-15 [35], and IFN-α [36]. Therefore, we first assayed for these cytokines in SEB-stimulated PBMC cultures. We found substantial quantities of IL-18 and IL-12p70, some IFN-α2, and only negligible quantities of IL-7 and IL-15 (Fig 6B).

Next, we assessed what proportion of SEB-stimulated CD3^+^IFN-γ^+^ cells expressed CD218a, the IL-18Rα chain and an integral part of the high affinity receptor for IL-18. We found a disproportionate pattern whereby the majority of IFN-γ^+^ T cells were among the CD218a^+^ population (approximately 80% at 24 h) (Fig 6C). This suggested that SEB-hyperresponsive T cells fell within a population that either constitutively expressed CD218a or had upregulated this molecule upon exposure to SEB. In our receptor expression analyses, SEB failed to increase the frequency of CD3^+^CD218a^+^ cells among PBMCs or the intensity of CD218a expression in T lymphocytes (S3A and S3B Fig).

Furthermore, in the absence of SEB stimulation and within total peripheral blood T cells, we found coincident expression of CD218a and CD161 (Fig 6D). Equally important, CD218a^high^CD161^high^ cells were almost exclusively Vα7.2^+^ MAIT cells (Fig 6D). In their steady state, most MAIT cells expressed CD218a along with CD212, the β1 subunit of the receptor for IL-12, a potent cytokine that cooperates with IL-18 to induce innate T cell activation [23,37]. In addition, and consistent with our findings in unfractionated CD3^+^ cells, SEB did not alter the expression levels of CD218a or CD212 in MAIT cells (Fig 6E). We found only a small fraction of resting T_conv_ cells to express CD218a and CD212 (S4 Fig). The frequency of CD218a^+^ T_conv_ cells only marginally increased upon SEB stimulation, and that of CD212^+^ cells was slightly reduced (S4 Fig). Consistent with this finding, neutralizing IL-18 and/or IL-12 failed to decrease the minute, but still detectable, TCR-dependent response of Vβ13.2^+^ T_conv_ to SEB (S5 Fig).

In the subsequent series of experiments, we evaluated the functional contribution of IL-18, IL-12 and IFN-γ signaling to MAIT cell responses to SAgs. Neutralizing IFN-γ did not prevent the accumulation of CD69^+^ (Fig 6F), IFN-γ^+^, TNF-α^+^, or IL-2^+^ MAIT cells in PBMC cultures (S6 Fig). In contrast, the frequency of cytokine-secreting MAIT cells was diminished partially by an anti-IL-12 mAb and almost completely by an IL-18-neutralizing mAb (Fig 6G and 6H). Moreover, co-neutralization of IL-12 and IL-18 led to a complete or near complete inhibition of cytokine production and CD69 expression by MAIT cells (Fig 6F and 6G). The inhibitory effects of IL-12 and IL-18 neutralization were evident not only for unfractionated MAIT cells but also for their Vβ13.2^+^ and Vβ2^+^ subsets (Fig 6H).

Finally, we compared MAIT cell responses to SEB, recombinant human IL-12 (rIL-12), and/or recombinant human IL-18 (rIL-18) in parallel. Treatment with rIL-12 or rIL-18 alone gave rise to CD69^+^ MAIT cells in culture (Fig 6I). When combined, these cytokines induced a stronger response, which was quantitatively similar to that triggered by SEB. When IFN-γ was used as the readout, rIL-18 alone did not induce a response above the background but further boosted the response to rIL-12, thus closely mimicking the SEB response (Fig 6I).

Altogether, the above findings indicate that: **i)** MAIT cells constitutively express high levels of IL-12R and IL-18R and are thus poised to respond to these cytokines during infection with SAg-producing bacteria; **ii)** the cytokine-mediated pathway of MAIT cell activation is dominant over the *i*TCR-dependent pathway during exposure to SAgs; **iii)** and this pathway is driven by IL-18 and IL-12 but not by IFN-γ at the outset.

### p38 and MEK1/2 control SEB-provoked, IL-12/IL-18-mediated MAIT cell transactivation

**Fig 7:**
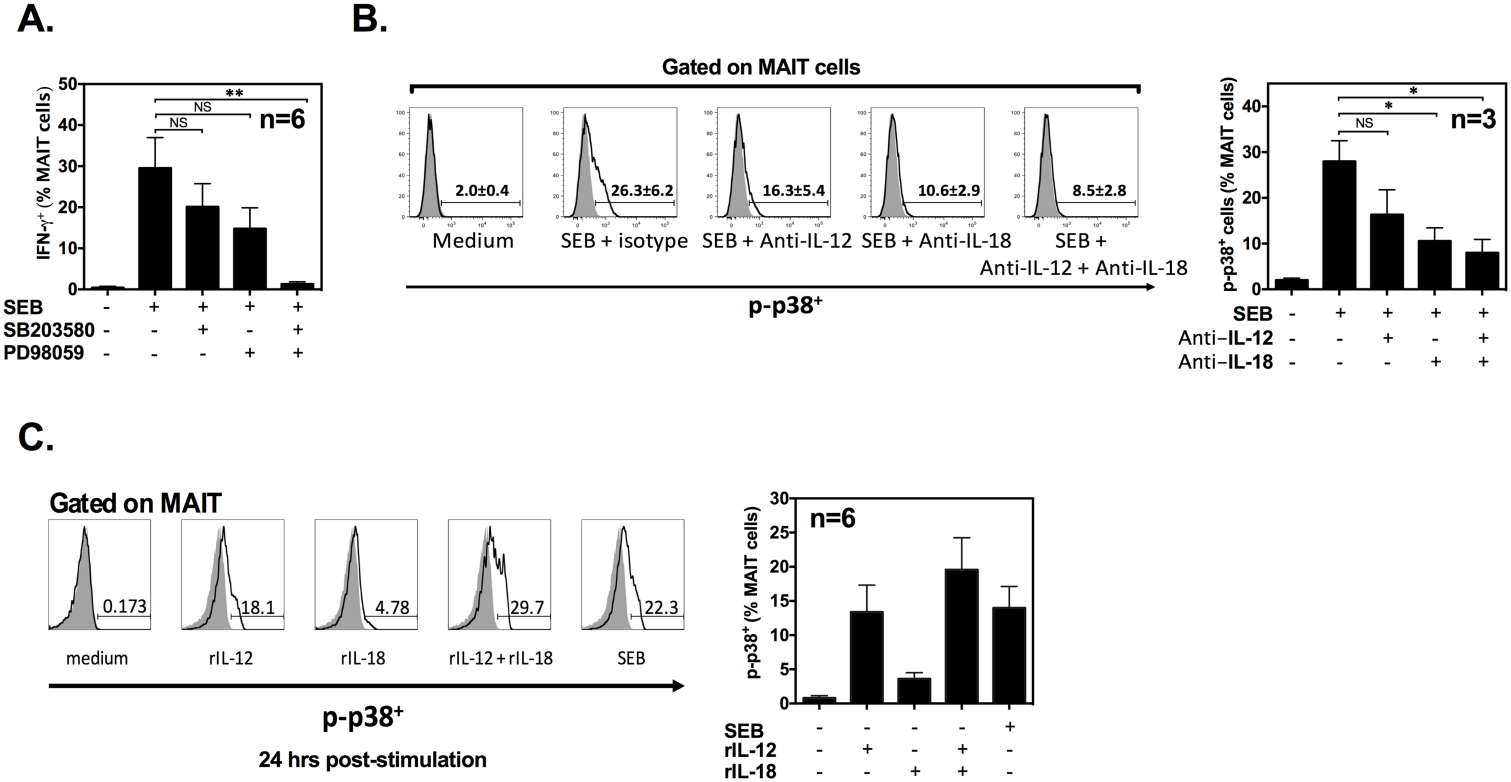
IL-12/IL-18-mediated transactivation of mucosa-associated invariant T (MAIT) cells following staphylococcal enterotoxin B (SEB) stimulation requires signaling through p38 and MEK1/2. Human peripheral blood mononuclear cells (PBMCs) (*n* = 6) were exposed to SEB in the absence or presence of 20 μM SB203580 and/or PD98059, and intracellular interferon (IFN)-γ accumulation in MAIT cells was detected after 24 h by flow cytometry (**A**). Intracellular phosphorylated p38 was traced in MAIT cells after stimulation with SEB in the absence or presence of anti-IL-12 and/or anti-IL-18 monoclonal antibodies (mAbs). Representative FACS plots are shown along with a bar graph summarizing data obtained from 3 individuals (*: *p* < 0.05; NS: non-significant) (**B**). In separate experiments, PBMCs were left untreated or exposed to SEB or to recombinant human IL-12 (rIL-12) and/or recombinant human IL-18 (rIL-18) for 24 h before intracellular p38 phosphorylation in MAIT cells was analyzed (*n* = 6). Error bars represent SEM (**C**). The underlying data for this figure can be found in S1 Data, and our gating strategies are provided in S2 Data.

Mitogen-activated protein kinases (MAPKs) have been implicated in cytokine-driven IFN-γ production by NK, *i*NKT, effector and memory Th1 cells [38–41]. However, whether they control MAIT cell transactivation is unknown. To delineate the intracellular pathway(s) governing MAIT cell responses to SAgs, we used pharmacological inhibitors of key intermediates of the MAPK signaling network. We found a marginal inhibition of the IFN-γ response when either SB203580 or PD98059 was present in cultures (Fig 7A).

However, a combination of these 2 inhibitors completely disabled IFN-γ production by MAIT cells (Fig 7A). Therefore, p38 and MEK1/2 work synergistically in MAIT cells to allow for a powerful IFN-γ response to SEB. SEB stimulation led to p38 phosphorylation within MAIT cells, which was inhibited by mAbs to IL-12 and IL-18 (Fig 7B). In addition, while rIL-18 generated a minute but still consistently detectable level of p38 phosphorylation, rIL-12 was much more efficient in this capacity (Fig 7C), and combined cytokine treatment was similar to SEB stimulation in inducing p38 phosphorylation in many MAIT cells. Therefore, MAIT cell activation by SEB that leads to IFN-γ production requires p38 and MEK1/2 kinases.

### MAIT cell hyperactivation by SEB renders them unresponsive to cognate Ags

**Fig 8:**
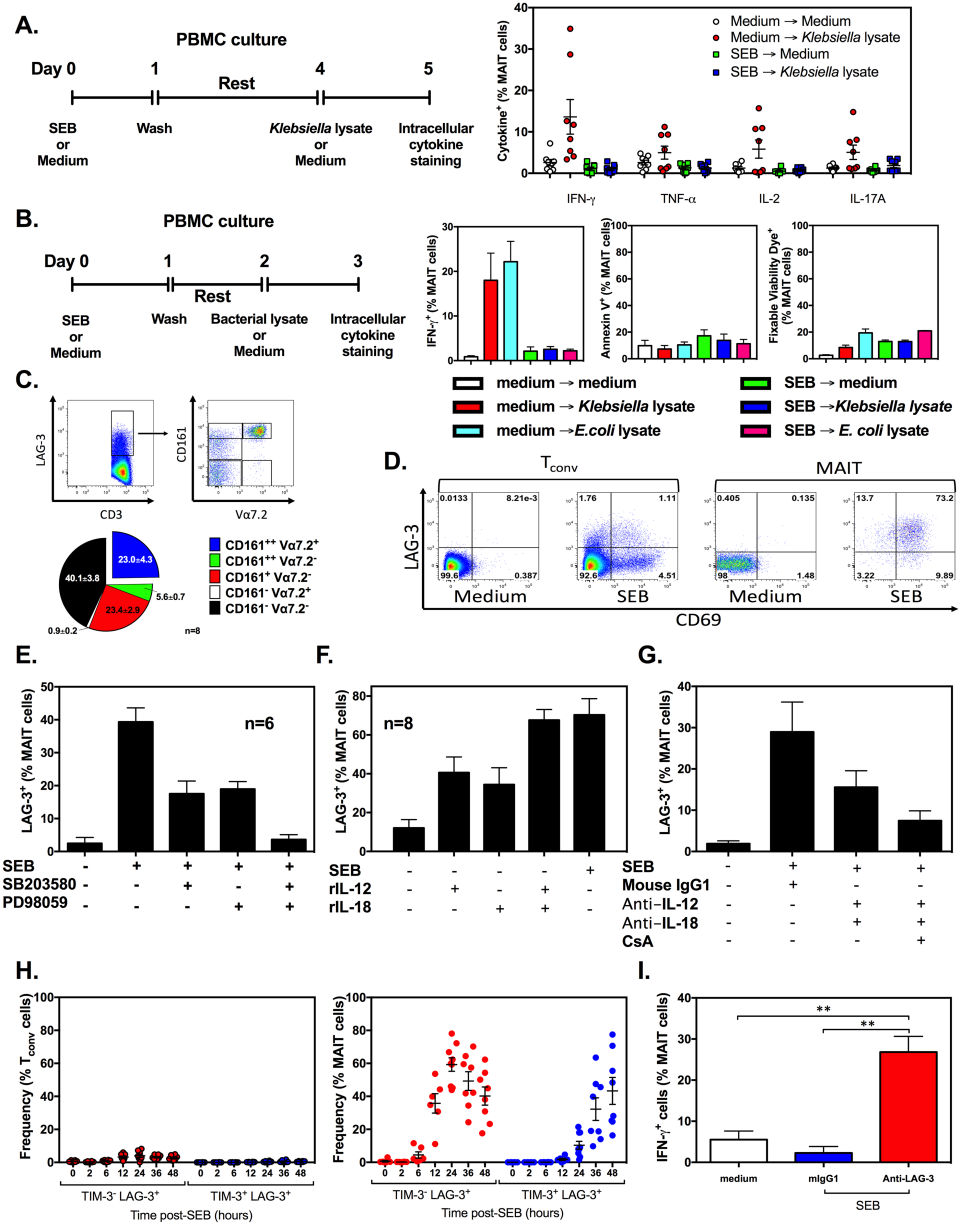
Staphylococcal enterotoxin B (SEB) stimulation of mucosa-associated invariant T (MAIT) cells results in lymphocyte-activation gene 3 (LAG-3)/ T cell immunoglobulin and mucin-3 (TIM-3) upregulation and interferes with their cognate antibacterial activity. Human peripheral blood mononuclear cells (PBMCs) (*n* = 8) were left untreated or stimulated for 24 h with SEB. They were then washed and rested for 3 days before they were exposed to *K*. *pneumoniae* lysate. Interferon (IFN)-γ^+^, tumor necrosis factor (TNF)-α^+^, interleukin (IL)-2^+^, and IL-17A^+^ events among MAIT cells were enumerated 24 h later by intracellular cytokine staining (**A**). PBMCs from a separate cohort were subjected to SEB stimulation, followed by 24 h of resting, before they were challenged with either *K*. *pneumoniae* or *E*. *coli* lysate. Twenty-four hours later, cells were interrogated for their intracellular IFN-γ content (*n* = 13) and evaluated for staining with Annexin V or Fixable Viability Dye (*n* = 4) (**B**). SEB-exposed CD3^+^ cells co-expressing LAG-3 were divided into 5 subpopulations based on CD161 and Vα7.2 staining. The relative contribution of each subpopulation to total CD3/LAG-3 double-expressors is depicted in a pie chart generated using PBMCs from 8 donors (**C**). Concomitant upregulation of CD69 and LAG-3 by SEB, or lack thereof, was also examined in conventional T (T_conv_) and MAIT cell compartments. Representative FACS plots are shown (*n* = 8) (**D**). In additional experiments, the frequencies of LAG-3^+^ MAIT cells were determined in PBMC cultures containing 20 μM SB203580 or PD98059 (**E**), 5 ng/mL recombinant human IL-12 (rIL-12) and/or recombinant human IL-18 (rIL-18) (**F**), 5 μg/mL anti-IL-12 and anti-IL-18 ± 200 ng/mL cyclosporine A (CsA) (*n* = 3) (**G**). The proportions of LAG-3^+^TIM-3^-^ and LAG-3/TIM-3 double-expressors among T_conv_ and MAIT cells were also calculated at indicated time points after SEB stimulation of PBMCs (*n* = 8) (**H**). In separate experiments, PBMC cultures were stimulated for 24 h with SEB. Cells were washed, and cultures were replenished with fresh medium containing 20 μg/mL of an anti-human LAG-3 monoclonal antibody (mAb) or a mouse IgG1 isotype control. *Klebsiella* lysate was added to cultures, followed, 24 h later, by enumeration of IFN-γ^+^ MAIT cells (*n* = 3) (**I**). Error bars represent SEM. The underlying data for this figure can be found in S1 Data, and our gating strategies are provided in S2 Data.

We found that human MAIT cells respond much more rigorously to SEB or to a combination of rIL-12 and rIL-18 than they do against lysates prepared from several bacterial pathogens known to harbor MR1-restricted Ags (S7 Fig). These microbes include *K*. *pneumoniae* [26,27], *E*. *coli* [42–44], *Pseudomonas aeruginosa* [45], *and Salmonella typhimurium* [17,42]. Therefore, we became curious if the enormity and the speed with which MAIT cells respond to SEB result in their exhaustion or anergy. We tested the ability of SAg-pre-exposed MAIT cells to respond to cognate stimulation. Twenty-four hours after incubation with SEB (or in medium as a control), PBMCs were washed and rested for an additional 72 h before they were challenged with *Klebsiella* lysate (Fig 8A).

A leading cause of community-acquired and nosocomial Gram-negative bacterial pneumonia [46], *K*. *pneumoniae* contains MAIT cell cognate Ags [26,27]. Despite resting in culture for 4 days, previously unstimulated MAIT cells were still capable of producing IFN-γ, TNF-α, IL-2, or IL-17A to *Klebsiella* Ags (Fig 8A). In stark contrast, SEB-exposed MAIT cells failed to produce these cytokines (Fig 8A). This was not due to cell death since only a very small fraction of MAIT cells stained positively with Annexin V (3.6%-7.2%) or retained the Fixable Viability Dye eFluor 780 (0.64%-1.75%) at the 24-h time point. Therefore, they were neither undergoing apoptosis nor dead. In a separate cohort (*n* = 13), we reduced the resting period to 24 h and additionally used *E*. *coli* lysate as a secondary challenge. Consistent with the data we obtained in the previous setup, SEB-exposed, but not unstimulated, MAIT cells were unresponsive to either bacterial challenge (Fig 8B). We also detected no tangible difference between the 3 groups in terms of Annexin V positivity or Fixable Viability Dye retention (Fig 8B). It is noteworthy that in a reverse experimental setting, stimulation of PBMCs with *Klebsiella* lysate prevented the MAIT cell response to SEB (S8 Fig). Therefore, it appears that repeated TCR triggering incapacitates MAIT cells. This theory is supported by our additional finding that priming PBMCs with *Klebsiella* lysate could also prevent the MAIT cells’ recall response to the same bacterial preparation (S8 Fig). On the contrary, TCR-independent signaling through IL-12 and IL-18 receptors enhanced, rather than attenuated, the IFN-γ response to *Klebsiella* (S8 Fig).

To explore the possibility of MAIT cell anergy or exhaustion in our system, we evaluated the expression of co-inhibitory molecules associated with these phenomena, including LAG-3, TIM-3 and PD-1. We found that a large proportion of CD3^+^ cells that co-expressed LAG-3 at 24 h post-SEB exposure were Vα7.2^+^CD161^high^ cells, thus fitting the phenotypic definition of MAIT cells (Fig 8C). Interestingly, while only a tiny subpopulation (approximately 1%) of T_conv_ cells were CD69^+^LAG-3^+^, more than 70% of MAIT cells co-expressed CD69 and LAG-3 (Fig 8D), suggesting that MAIT cell hyperactivation and anergy programs are simultaneously set in motion by SEB stimulation. By the same token, a combination of SB203580 and PD98059 prevented SEB-induced LAG-3 up-regulation (Fig 8E), indicating that both p38 and MEK1/2 were required for the observed phenotype. This was reminiscent of the role played by MAPKs in IFN-γ production by MAIT cells (Fig 7A), which supports the notion that SEB-induced MAIT cell activation and anergy go hand in hand.

SEB-induced up-regulation of LAG-3 on MAIT cells appears to rely on IL-12 and IL-18. First, SEB and a combination of these cytokines elevated the population size of LAG-3^+^ MAIT cells to a comparable level (Fig 8F). Second, co-neutralizing IL-12 and IL-18 led to a 50% reduction in the frequency of this population (Fig 8G). Since the observed inhibition was incomplete, we asked whether *i*TCR signaling was also required for full induction of LAG-3. Indeed, when we used the nuclear factor of activated T cells (NFAT) inhibitor cyclosporine A (CsA) in conjunction with anti-IL-12 and anti-IL-18, LAG-3^+^ MAIT cell proportions dropped further (Fig 8G).

Investigating the potential role of other co-inhibitory molecules, we found that most LAG-3^+^ MAIT cells did not co-express TIM-3 at earlier time points (Fig 8H). However, after 12 h of SEB stimulation, LAG-3^+^TIM-3^+^ cells were easily detectable within the MAIT cell population but not among T_conv_ cells (Fig 8H). Finally, only few MAIT cells (approximately 10%) expressed PD-1 by 48 h post-SEB stimulation.

To ascertain the significance of early LAG-3 upregulation in SEB-induced MAIT cell anergy, we added a LAG-3-blocking mAb or a mouse IgG1 isotype control to PBMC cultures 24 h after SEB stimulation. Cells were then rested for 24 h before they were challenged with *Klebsiella* and examined, 24 h later, for their intracellular IFN-γ content (Fig 8I). Importantly, the blockade of LAG-3 restored the ability of MAIT cells to respond to *Klebsiella* Ags, further indicating that exposure to SEB does not simply kill MAIT cells but renders them anergic.

In summary, we conclude that: **i)** hyperactivation of MAIT cells by SEB interferes with their ability to respond to bacterial pathogens; **ii)** the failure of MAIT cells to produce cytokines in response to cognate Ags is accompanied by LAG-3 and TIM-3 upregulation; **iii)** SEB-induced MAIT cell anergy can be reversed by blocking LAG-3; **iv)** and a cytokine-dominant pathway dictates both the hyperactivation and the anergy of MAIT cells in the aftermath of an SEB challenge.

### In vivo exposure to SEB results in mouse MAIT cell anergy

In the next series of experiments, we investigated whether SAgs induce MAIT cell anergy in vivo. SEB exhibits poor affinity for MHC class II molecules expressed in certain mouse strains, such as B6 mice [29]. Therefore, we and others have routinely used DR4 tg mice in which high-affinity interactions with SEB induce rigorous host responses to this SAg, thus simulating many aspects of clinical TSS [9,47–50].

**Fig 9:**
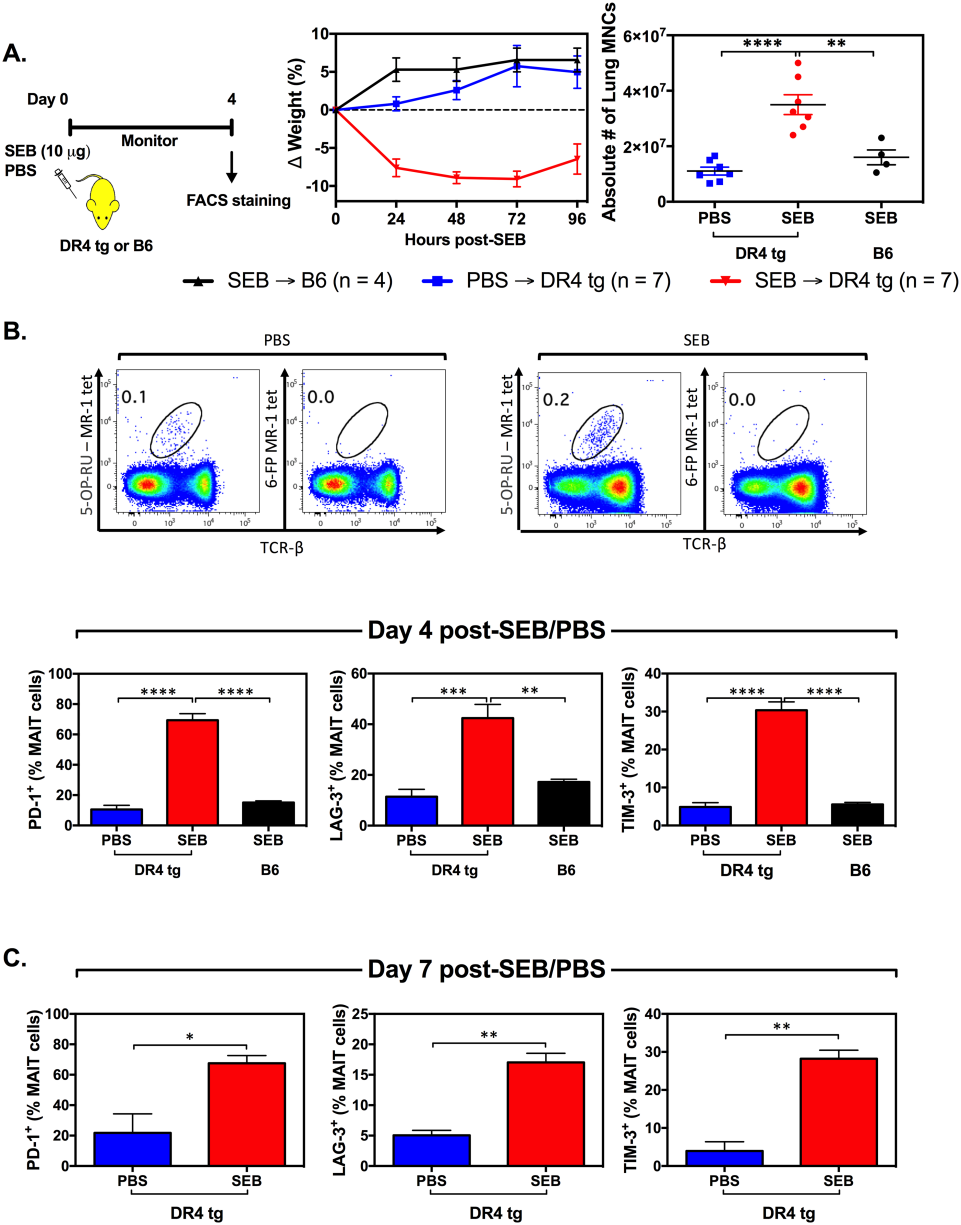
In vivo exposure to staphylococcal enterotoxin B (SEB) causes weight loss, non-parenchymal lung mononuclear hypercellularity, and mucosa-associated invariant T (MAIT) cell anergy in DR4-transgenic (DR4 tg) mice. Wild-type B6 and DR4 tg mice (*n* = 4 or *n* = 7 as indicated) were injected intraperitoneally (i.p.) with 10 μg SEB or with PBS and monitored for weight loss (**A**). On day 4 post-SEB/PBS injection, mice were euthanized, and total non-parenchymal lung mononuclear cells (MNCs) were enumerated (**A**). In addition, lung MAIT cells were detected via co-staining with an anti-mouse T cell receptor (TCR)β monoclonal antibody (mAb) and 5-OP-RU-loaded mouse MHC-related protein 1 (MR1) tetramer, and further evaluated for their expression levels of PD-1, LAG-3 and TIM-3 (**B**). In a separate cohort of DR4 tg mice (*n* = 3), PD-1, LAG-3 and TIM-3 expression by lung MAIT cells was assessed on day 7 post-SEB/PBS injection (**C**). Error bars represent SEM, and *, **, ***, and **** denote *p* < 0.05, *p*< 0.01, *p* < 0.001, and *p* <0.0001, respectively. The underlying data for this figure can be found in S1 Data, and our gating strategies are provided in S2 Data.

A single, low-dose (10 μg) intraperitoneal (i.p.) injection of SEB into DR4 tg mice caused morbidity in these animals as judged by their weight loss. In contrast, SEB-injected wild-type B6 mice and PBS-injected DR4 tg mice showed no signs of morbidity as anticipated (Fig 9A).

We noticed a significant numerical increase in non-parenchymal lung mononuclear cells (MNCs) in SEB-exposed DR4 tg mice (Fig 9A). This was of interest since lungs can attract a substantial number of MAIT cells during infection [21]. We found dramatic increases in PD-1^+^, LAG-3^+^ and TIM-3^+^ cells among lung MAIT cells in DR4 tg mice 4 days (Fig 9B) or 7 days (Fig 9C) after SEB administration. Mouse MAIT cells in these experiments were identified through co-staining with an anti-mouse TCRβ mAb (or anti-mouse CD3 mAb) and 5-(2-oxopropylideneamino)-6-D-ribitylaminouracil (5-OP-RU)-loaded mouse MR1 tetramer reagents [19].

Unlike in humans, MAIT cells are infrequent in laboratory mouse strains such as B6 mice [51] and in DR4 tg mice (Please read below). This is with the exception of CAST/EiJ mice that harbor an unusually high number of MAIT cells, approximately 20 times more than B6 mice, in their T cell repertoire [51]. We found CAST-EiJ mice to be responsive to SEB (S9 Fig).

**Fig 10:**
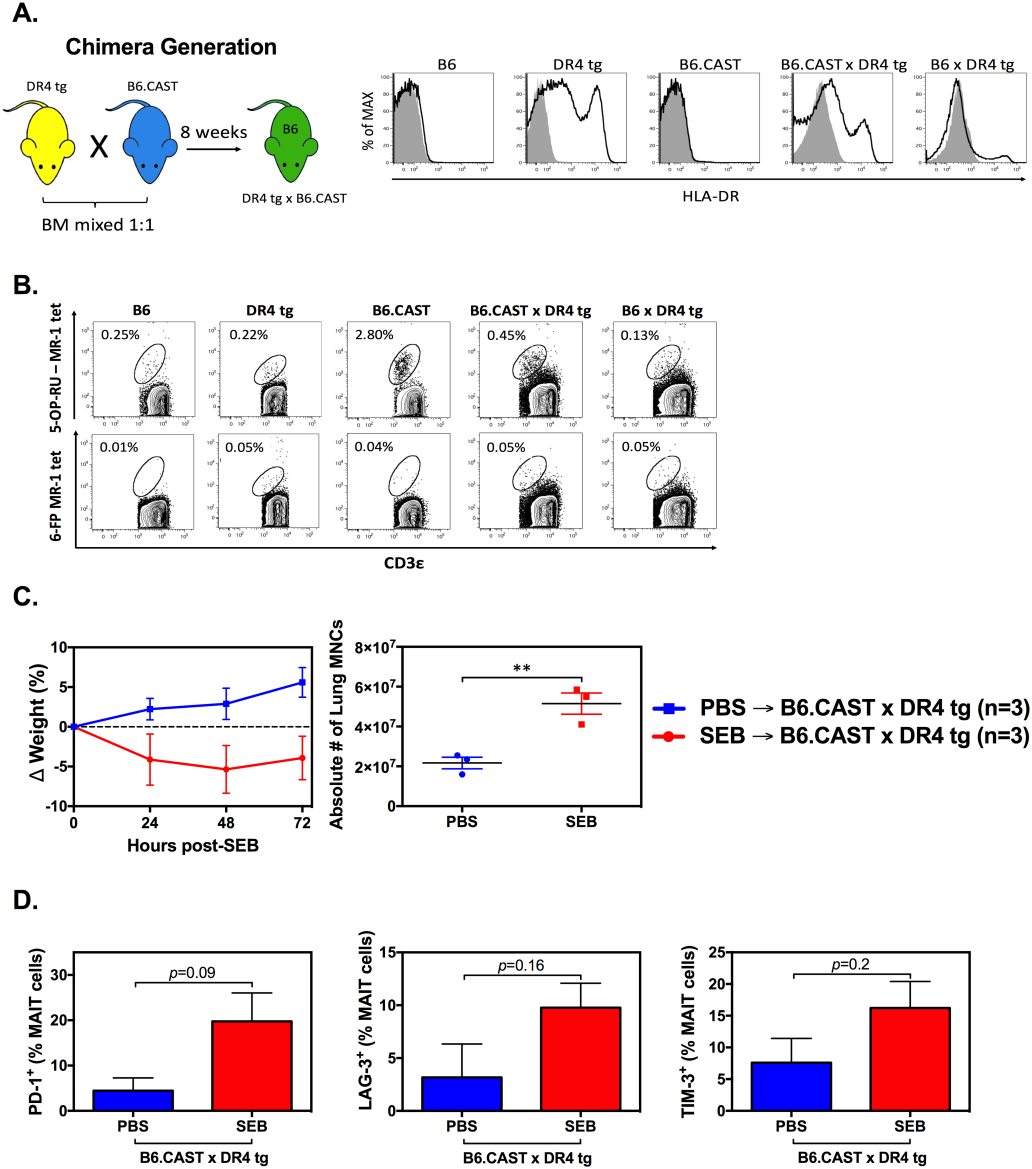
Staphylococcal enterotoxin B (SEB) administration to mucosa-associated invariant T (MAIT)^high^ DR4^+^ chimeric mice upregulates anergy markers on MAIT cells. Bone marrow cells from DR4-transgenic (DR4 tg) and B6.CAST mice were prepared and co-injected at a 1:1 ratio into γ-irradiated B6 mice. Control chimeras were generated by co-transferring B6 and DR4 tg marrow cells into B6 recipients. The expression of HLA-DR, or lack thereof, was assessed by flow cytometry in the above chimeras as well as in wild-type B6, DR4 tg, and B6.CAST mice. Filled histograms correspond to background staining with a mouse IgG2a isotype control (**A**). In addition, lung MAIT (CD3^+^ 5-OP-RU-MR1 tetramer^+^) cell frequencies in the above animals were determined (**upper panels in B**). 6-formylpterin (6-FP)-loaded MR1 tetramer was used as a negative staining control (**lower panels in B**). Chimeric mice (*n* = 3 per group) were injected i.p. with PBS or with 10 μg SEB, monitored for weight loss, and sacrificed on day 3, at which point total non-parenchymal lung mononuclear cells (MNCs) were enumerated (**C**) and PD-1^+^, LAG-3^+^ and TIM-3^+^ lung MAIT cell proportions were determined by flow cytometry (**D**). Error bars represent SEM, and ** denotes *p* < 0.01. The underlying data for this figure can be found in S1 Data, and our gating strategies are provided in S2 Data.

However, CAST-EiJ and DR4 tg mice could not be directly compared due to their different genetic backgrounds. A MAIT^high^ congenic strain on the B6 background (B6.CAST mice) has been recently generated [51]. However, these mice express MHC class II molecules of B6 origin that have inadequate affinity for SEB. Therefore, to confirm MAIT cell anergy in SAg-responsive, MAIT cell-sufficient mice, which more closely resemble humans, we generated B6.CAST×DR tg bone marrow chimeras. We first verified the expression of HLA-DR4 in DR4 tg mice, B6.CAST×DR tg chimeras, and B6×DR tg chimeric controls, but not in wild-type B6 and B6.CAST mice (Fig 10A).

Using 5-OP-RU-loaded mouse MR1 tetramer reagents, we also confirmed heightened MAIT cell frequencies in the lungs of B6.CAST×DR tg chimeras in comparison with B6×DR tg controls (Fig 10B). There was no reactivity with 6-formylpterin (6-FP)-loaded MR1 tetramer, which was used as a staining control. Consistent with our findings in DR4 tg mice (Fig 9), B6.CAST×DR tg chimeras gradually lost weight and exhibited lung mononuclear hypercellularity following SEB administration (Fig 10C). In addition, we observed a trend towards higher proportions of PD-1^+^, LAG-3^+^ and TIM-3^+^ cells among lung MAIT cells in these animals (Fig 10D).

Together, the above results indicate that mouse MAIT cells are anergized after in vivo exposure to SEB.

### Systemic administration of SEB anergizes human MAIT cells in vivo

Human PBMC-reconstituted NSG (hPBMC-NSG) mice provide a valuable model in which to study human immune responses in an in vivo setting. This model offers an additional advantage for our purpose—that is, cellular responses to SAgs can be deciphered in the absence of severe morbidity. This is because non-hematopoietic cells (e.g., endothelial cells, intestinal, and respiratory epithelial cells) of hPBMC-NSG mice lack human receptors that mediate the adverse manifestations of SAg-mediated illnesses. However, it is still possible to quantify human cytokines in the circulation and to assess human leukocytes for phenotypic changes indicative of anergy/exhaustion.

**Fig 11:**
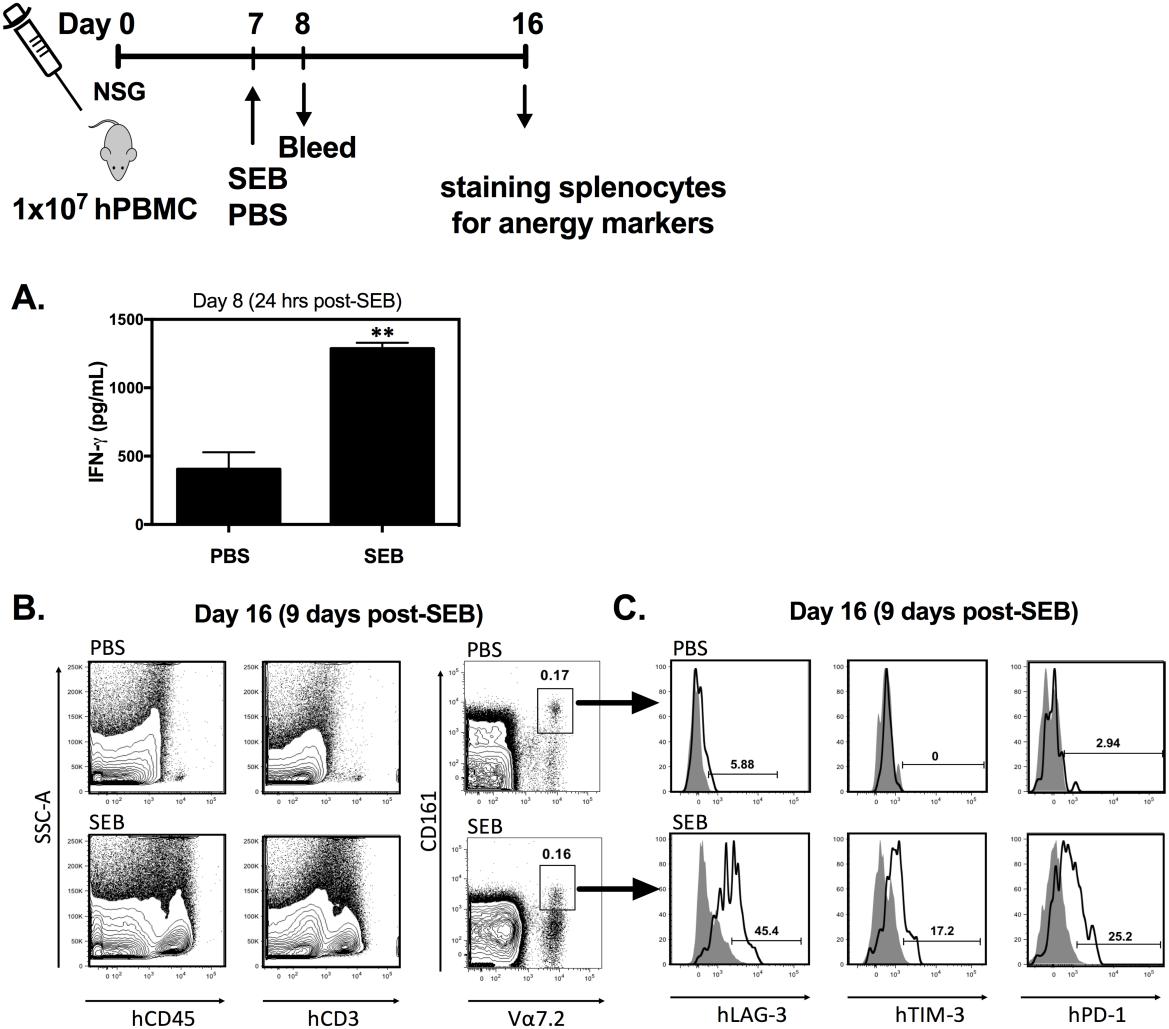
Staphylococcal enterotoxin B (SEB) induces rapid expression of LAG-3, TIM-3 and PD-1 by human mucosa-associated invariant T (MAIT) cells in vivo. Human peripheral blood mononuclear cell—reconstituted NOD-*scid* IL-2Rγ^null^ (hPBMC-NSG) mice (*n* = 3/group) were injected with PBS or 100 μg SEB. Twenty-four hours later, human interferon (IFN)-γ was measured in the serum by ELISA. Error bars represent SEM, and ** denotes *p* < 0.01 (**A**). Nine days after SEB injection, splenic cells were stained with anti-human CD45, CD3, CD161, and Vα7.2 monoclonal antibodies (mAbs) to confirm the presence and/or expansion of human hematopoietic cells, T cells and MAIT cells in hPBMC-NSG mice (**B**). In addition, MAIT cells were assessed for surface expression of human LAG-3, TIM-3 and PD-1 (open histograms). Filled histograms represent background staining with isotype controls (**C**). The underlying data for this figure can be found in S1 Data, and our gating strategies are provided in S2 Data.

We found that a single i.p. injection of SEB results in an approximately 3-fold increase in the serum concentration of IFN-γ (Fig 11A) and also confirmed that both the human CD45^+^ hematopoietic cell population and the human CD3^+^ T cell population are expanded by SEB (Fig 11B).

Vα7.2^+^CD161^high^ MAIT cells were present in hPBMC-NSG mice but did not show any sign of expansion by SEB, at least on day 9 post-stimulation, a time point at which the general T cell population size was markedly enlarged (Fig 11B).

Unlike PBS-injected control mice, SEB-primed mice exhibited substantial expression of human LAG-3, TIM-3 and PD-1 on their splenic MAIT cells (Fig 11C). Finally, as with our in vitro human PBMC cultures, LAG-3 was apparently the main co-inhibitory molecule expressed by SEB-exposed MAIT cells. Therefore, the hPBMC-NSG mouse model validates our in vitro findings pertaining to human MAIT cell anergy.

## Discussion

MAIT cells are thought to participate in host defense against a wide range of bacteria and fungi. These include *K*. *pneumoniae*, *E*. *coli*, *P*. *aeruginosa*, *S*. *typhimurium*, *S*. *aureus*, *Staphylococcus epidermidis*, *Candida albicans*, *C*. *glabrata*, and *Saccharomyces cerevisiae* among other microbes [17,20–22,26,27,42–45]. In this work, we have defined a pathogenic, as opposed to protective, role for MAIT cells during infection.

While investigating the early sources of IFN-γ among human PBMCs following exposure to SEB, a prototypical staphylococcal SAg, we found a subpopulation of T cells that expressed a very high level of CD161 (>100-fold higher than that expressed by the general T cell population) and also harbored a Vα7.2 TCR α chain. This finding and our subsequent experiments led to the identification of MAIT cells as extremely potent and fast-acting producers of pro-inflammatory cytokines in response to bacterial SAgs. Therefore, MAIT cells are likely to be a key effector of the characteristic cytokine storm associated with these potentially deadly toxins.

The tissue distribution of MAIT cells may affect their inflammatory cytokine profiles. In addition, the type of cytokine sets secreted by MAIT cells is dictated by the experimental conditions or by the nature of the stimuli employed. For instance, Dusseaux et al. reported that human blood MAIT cell stimulation with phorbol 12-myristate 13-acetate (PMA) and ionomycin leads to IFN-γ, TNF-α, IL-2 and IL-17 production [11]. In contrast, human MAIT cell co-culture with *E*. *coli*-fed autologous monocytes resulted in secretion of IFN-γ, but not IL-2, and a mixed IL-17 response [11]. A subsequent study by Tang et al. found that hepatic MAIT cells produce more IL-17 in response to PMA and ionomycin than their blood counterparts do [12]. However, when liver or blood MAIT cells were incubated with anti-CD3/CD28-coated beads, IL-17 production was not induced at either mRNA or protein level. As another example, MAIT cells isolated from the fetal small intestine, but not those harvested from the fetal liver or lungs, respond to *E*. *coli* and an agonistic anti-CD28 mAb by secreting large quantities of IL-22, a cytokine that modulates parenchymal tissue responses during inflammation [28]. The findings of these studies suggest that *i*TCR-dependent and -independent stimulation of MAIT cells yield distinct cytokine responses. In the current study, stimulation with SEB resulted in robust induction of IFN-γ and TNF-α and moderate up-regulation of IL-2 in both peripheral blood and liver MAIT cells. However, there was no appreciable IL-17A response, and the frequency of RORγt^+^ MAIT cells, in fact, decreased after exposure to SEB.

We found that SEB-provoked MAIT cell activation does not involve MR1 but requires MHC class II molecules. Since human MAIT cells express HLA-DR [12], a scenario can be envisaged in which MAIT cells cross-activate each other when incubated with SEB. However, we observed no cytokine production by SEB-exposed, purified MAIT cells in the absence of autologous monocytes. This rules out the above possibility and indicates a requirement for accessory molecules, which would perhaps supply costimulatory signals, for maximal MAIT cell responses to SAgs.

SEB activates MAIT cells through *i*TCR ligation and IL-18R/IL-12R signaling, although the latter pathway appears dominant. Therefore, whether MAIT cells produce IL-17 is not simply a matter of the presence or absence of *i*TCR signaling. Our preliminary findings suggest that the choice to launch the RORγt/IL-17 program is not influenced by internalization of a critical number of *i*TCRs in MAIT cells either. This is because stimulation with anti-CD3/CD28 mAbs, but not SEB, resulted in rapid and complete *i*TCR internalization, thus making MAIT cells undetectable (S10 Fig); yet, both stimuli failed to induce IL-17.

We previously reported that SEB-exposed mouse and human *i*NKT cells similarly fail to down-regulate their Vα14-Jα18 and Vα24-Jα18 *i*TCR versions, respectively [9]. Furthermore, *i*NKT cells are indispensable for SEB-induced early IL-17 production [50]. Whether these findings are coincidental or mechanistically linked remains to be elucidated.

We found that even in the absence of “SAg-responsive” TCR Vβ chains, human MAIT cells can be activated in an IL-12/IL-18-dependent, bystander fashion. MAIT cells expressed high levels of the receptors for these cytokines and were the main, if not the only, population of CD218a^high^CD161^high^ cells among PBMCs. A combination of rIL-12 and rIL-18 was able to mimic the SEB response in several experiments. However, unlike rIL-12, rIL-18 alone induced little IFN-γ production, which is consistent with its role as a “co-factor” rather than a driver of Th1-type responses [52,53]. IL-12 stimulation can increase the expression of IL-18R on human T cells, leading to IFN-γ production [54]. On the other hand, IL-12 and IL-18 use signal transducer and activator of transcription (STAT) 4 and activator protein 1 (AP-1) in their signaling pathways, respectively, and STAT4 potentiates AP-1-mediated IFN-γ promoter activation without directly binding to it [55]. This is significant because AP-1 can only weakly interact with an AP-1-binding region within the IFN-γ promotor, and IL-18 can only minimally induce an IFN-γ response on its own. Since MAIT cells express IL-18R constitutively, we postulate that IL-18 and IL-12 cooperate through the latter mechanism. Intriguingly, neutralization of IL-18 alone was more potent than that of IL-12 in multiple set-ups. This may be a unique feature of MAIT cell activation in response to SAgs because anti-IL-12 and anti-IL-18 were equally efficient in inhibiting the minute response mounted by T_conv_ cells against SEB (S11 Fig).

Stimulation with rIL-12 and/or IL-18 in our system does not involve *i*TCR ligation, and when endogenous IL-12 and IL-18 are neutralized, *i*TCR triggering by SEB is still present. This may reconcile, at least partially, our seemingly paradoxical observations on exogenous IL-18 stimulation versus endogenous IL-18 neutralization. In fact, IL-18 and TCR signaling have been recently demonstrated to work synergistically to induce IFN-γ secretion by human γδ T cells [56], another type of innate-like T cells, which lends support to the above hypothesis.

In addition to cytokine production, MAIT cells also reportedly exert direct cytotoxic effector functions [43]. In preliminary experiments, we have found that following SEB stimulation of human PBMCs, the frequencies of CD107a^+^GZM A^+^, CD107a^+^GZM B^+^, and GZM A^+^GZM B^+^ cells increase dramatically in the MAIT cell compartment but only minimally among T_conv_ cells (S12A Fig), a response that was partially inhibited by IL-12/IL-18 co-neutralization (S12B Fig). Therefore, SAgs can induce MAIT cell—mediated cytotoxicity with unclear outcomes at this point, a subject that we are currently investigating.

SAgs can enter the systemic circulation in the absence of overt bacteremia, a condition that is perhaps best exemplified by menstrual TSS. It has also been suggested that SAgs liberated in the gastrointestinal tract by food-borne pathogens may cross the perturbed gut mucosa either directly or with the assistance of other bacterial toxins [57,58] and consequently access the liver via the portal vein [12]. The abundance of MAIT cells in the intestine and in the liver inevitably puts them in a unique position to respond to SAgs with deleterious outcomes.

SAgs may also contribute to the pathogenesis of sepsis [59,60]. During polymicrobial sepsis, certain common bacteria (e.g., *Staphylococcus* spp. and *Streptococcus* spp.) likely release the SAgs they harbor. However, predicting the net effect is not easy. First, the type and combination of microbes involved may vary in different patients. Second, how host responses to multiple SAgs secreted by multiple pathogens may cross-regulate each other is ill-defined. Third, SAg-induced responses are modulated by Toll-like receptor (TLR) ligands embedded in the cell wall of the very bacteria that secrete them, as we previously described [47]. Finally, we recently reported that SAgs promote bacterial colonization and infection [61], although the significance of this phenomenon in the initial stages of sepsis is unclear. It will be important to assess MAIT cell functions in polymicrobial infections. A prospective study by Grimaldi et al. found an early and selective decline in MAIT cell blood counts of patients with severe sepsis and septic shock [62]. Interestingly, the cumulative incidence of intensive care unit (ICU)-acquired infections in patients inversely correlated with their peripheral blood MAIT cell numbers.

We believe that pro-inflammatory cytokine (especially IFN-γ) production in excessive quantities by MAIT cells, in comparison with T_conv_, *i*NKT and γδ T cells, is not the only mechanism by which MAIT cells may inflict harm. Our findings also implicate these cells in SAg-associated immunosuppression. Several mechanisms have been proposed to contribute to this phenomenon. Exposure to SAgs may delete or anergize many T cells [5,6], thus physically or functionally depleting a fraction of T cells with antimicrobial specificities or properties. Regulatory or suppressor cell types may also take part in SAg-mediated immunosuppression. In vitro stimulation of human PBMCs with SEA, SpeA, and SpeK/L converts CD25^-^ T_conv_ cells to IL-10–producing CD25^+^FoxP3^+^ regulatory T (Treg) cells [63]. However, Tilahun et al. demonstrated that the expansion of endogenous Treg cells or their adoptive transfer into HLA-DR3 transgenic mice fails to prevent SEB-provoked T cell proliferation or the organ damage sustained in these animals [64]. This calls into question the in vivo significance of Treg cells in SAg-mediated illnesses. Additionally, not all immunosuppressive mechanisms are detrimental to the host. We recently reported a profound and tissue-selective influx of granulocytic myeloid-derived suppressor cells (MDSCs) into the liver of DR4 tg mice shortly after an SEB challenge [49]. In this model, MDSCs attenuated SEB-induced T cell proliferation, which prompted us to propose that their local accumulation in the liver may benefit the host by ameliorating SAg-induced tissue damage.

It needs to be emphasized that SAg-induced T_conv_ cell anergy has been documented in mouse models. It is not completely clear whether human T cells, especially the subsets that are poised to swiftly respond to pathogens, undergo anergy following exposure to SAgs. We report, herein, that human MAIT cell hyperactivation by SEB is accompanied by the acquisition of an anergic state that hinders their antimicrobial functions, for instance, against *K*. *pneumoniae* and *E*. *coli*. Accordingly, we propose a novel mechanism of immunosuppression in the human T cell compartment. In addition, using MAIT^low^ and MAIT^high^ HLA-DR4 transgenic mice as well as humanized NSG mice, we have documented that MAIT cell anergy can occur in vivo.

SEB-induced MAIT cell anergy was primarily accompanied by LAG-3 upregulation, which was evident not only in human PBMC cultures but also in humanized NSG mice. We hypothesize that LAG-3 upregulation on a large proportion of SEB-exposed MAIT cells confers upon them an anergic state that would prevent their optimal MR1-restricted responses against microbial pathogens. LAG-3 is known to interact with MHC class II molecules expressed by Ag-presenting cells [65,66]. It is tempting to speculate that LAG-3 upregulation by MAIT cells enables them to compete with CD4^+^ T_conv_ cells for access to MHC class II, thus potentially interfering with helper T cell functions, which would in turn cripple a critical arm of adaptive immunity to microbes.

Several lines of evidence suggest that MAIT cell activation and anergy involve the same pathway. First, SEB-induced upregulation of CD69 and LAG-3 follow the same kinetics. Second, IFN-γ and LAG-3 induction both depend on p38 and MEK1/2 MAP kinases. Third, CD69 upregulation, pro-inflammatory cytokine production, p38 phosphorylation and LAG-3 induction are comparably inhibitable by IL-12/IL-18 co-neutralization. Fourth, all these phenotypic changes are inducible with strikingly similar magnitudes by SEB and IL-12/IL-18 stimulation.

Based on the findings of the current investigation, we believe that MAIT cells may constitute attractive therapeutic targets in the context of SAg-mediated illnesses. First, they are naturally enriched at anatomical ports of entry for many SAg-producing microbes. Second, they mount massive pro-inflammatory cytokine responses almost immediately after their encounter with SAgs. Therefore, blocking their function may help mitigate the cytokine storm caused by these toxins in a timely fashion. Third, interfering with MAIT cell hyperactivation may also prevent their anergy and the suppression of certain antimicrobial defense mechanisms. Fourth, MR1, the restriction element for MAIT cells, is monomorphic [16]. Therefore, MR1-restricted MAIT cell antagonists, similar to recently described MAIT cell ligands [17,44], should potentially work in genetically diverse human populations.

## Materials and methods

### Ethics statement

Animal experiments were performed following an animal care protocol (AUP# 2010–241) approved by the Animal Care Committee of Animal Care and Veterinary Services at Western University and in compliance with the Canadian Council on Animal Care guidelines. Human samples were collected after informed written consent was obtained and according to protocols approved by the Western University Research Ethics Board for Health Sciences Research Involving Human Subjects (approval numbers: HSREB 5545 and HSREB 106937).

### Human PBMC and HMNC isolation

PBMCs were isolated from heparinized whole blood of healthy donors by density gradient centrifugation using low-endotoxin (<0.12 EU/mL) Ficoll-Paque PLUS (GE Healthcare Life Sciences) and 50-mL SepMate tubes (Stemcell Technologies Inc., Vancouver, BC), as per manufacturer’s instructions. HMNCs were immediately extracted from tumor-free liver tissues surgically removed from patients undergoing liver resection for colorectal carcinoma metastasis at the London Health Sciences Centre University Hospital (London, ON). Tissue samples were pressed through a wire mesh, and the resulting homogenate was washed in 2% fetal calf serum (FCS) in cold PBS. Pelleted cells were washed again, placed in 33.75% low-endotoxin Percoll PLUS (GE Healthcare Life Sciences), and spun at 700 × *g* for 12 min at room temperature. Pelleted cells were then treated with ACK lysis buffer for 2 min to lyse erythrocytes and washed before a nylon mesh strainer with 70-μm pores was used to remove clumps and debris.

### Mice

Adult, female C57BL/6 (B6) mice were from Charles River Canada Inc. (St. Constant, QC). B6.CAST mice [51] and DR4 tg mice on a B6 background were housed and bred in a barrier facility at Western University. DR4 tg mice lack endogenous MHC II molecules and instead express a chimeric HLA molecule that is composed of HLA-DRA-IEα and HLA-DRB1*0401-IEβ [67]. CAST/EiJ mice and NSG mice were purchased from The Jackson Laboratory (Bar Harbor, ME). NSG mice were partially humanized via an i.p. injection of 1 × 10^7^ human PBMCs.

### Generation of mixed bone marrow chimeras

B6, B6.CAST and DR4 tg mice were sacrificed by cervical dislocation, and bone marrow was flushed, using 5-mL RPMI 1640 medium, out of femurs and tibias. Marrow cells were spun, exposed to ACK lysis buffer for 2 min to remove erythrocytes, washed in sterile PBS, and passed through a 70-μm nylon mesh strainer. Bone marrow cells from DR4 tg mice were mixed at a 1:1 ratio with either B6.CAST or wild-type B6 marrow cells. Two million mixed cells were injected intravenously (i.v.) into wild-type B6 recipients, which were lethally irradiated at 1,100 cGy using a ^137^Cs γ-irradiator prior to adoptive transfer. Reconstituted recipients were provided with drinking water supplemented with 2 mg/mL neomycin sulfate to prevent infection. Eight weeks after reconstitution, chimeric mice were injected with PBS or SEB as indicated.

### Mouse BMDC culture

Bone marrow cells from wild-type B6 or DR4 tg mice were prepared as described above. In a T75 flask, cells were seeded at a density of 1 × 10^6^ cells/mL in RPMI 1640 medium containing 10% heat-inactivated FCS, GlutaMAX-I, 0.1 mM MEM nonessential amino acids, 1 mM sodium pyruvate, 100 U/mL penicillin, 100 μg/mL streptomycin, and 10 mM HEPES (complete medium), which was further supplemented with 10 ng/mL mouse GM-CSF and IL-4 (PeproTech Inc., Rocky Hill, NJ). Cultures were replenished with fresh medium and cytokines every other day after discarding the floating cells. On day 7, harvested cells were enriched for CD11c^+^ DCs using an EasySep Mouse CD11c Positive Selection Kit (Stemcell Technologies).

### MAIT cell lines

Mouse MAIT hybridoma lines 8D12, 6C2, and 17E6 [14,16] were grown in complete medium and maintained at 37°C in a humidified atmosphere containing 6% CO_2_. The expression of TCR Vβ8 by these hybridomas, or lack thereof, was verified by flow cytometry after staining with a FITC-conjugated anti-Vβ8.1/Vβ8.2 mAb (clone KJ16-133) or a rat IgG2a isotype control.

### Bacterial SAgs

Recombinant SAgs were made using an approved institutional biosafety protocol adhering to the Public Health Agency of Canada regulations. SEB was cloned from *S*. *aureus* (strain COL), expressed in BL21 (DE3) competent *E*. *coli*, and purified by nickel column chromatography [47]. SEA and TSST-1 were generated using a similar procedure. SpeA and SpeI were made and purified as previously described [31,68]. Using site-directed mutagenesis, we also generated a largely inactive form of SEB that carries an N→A point mutation at position 23, which is essential for optimal binding to mouse TCR Vβ8.2 [30]. As in our past studies [9,48,49], we used this mutant, which we refer to as SEB_N23A_, as a negative control.

### Preparation of bacterial lysate

A frozen stock of a *K*. *pneumoniae* clinical isolate, Parkwood-18, was a gift from Dr. Miguel Valvano (Queen’s University Belfast, Belfast, United Kingdom). *P*. *aeruginosa* (ATCC 27853) was generously provided by Dr. Lori Burrows (McMaster University, Hamilton, Canada). We also generated lysate from *E*. *coli* strain DH5α and *S*. *typhimurium* strain LT2 (ATCC 700720).

*K*. *pneumoniae*, *E*. *coli*, and *S*. *typhimurium* were grown in Luria broth, and *P*. *aeruginosa* was grown in Tryptic Soy Broth. Following overnight culture at 37°C, bacterial cells were washed 3 times in PBS, and the OD_600_ was adjusted to 2.0 or 6.5 (in the case of *K*. *pneumoniae*). *Klebsiella* cells were subjected to pressure at 30,000 pounds per square inch (PSI) for 5 min to induce membrane rupture. For all other bacteria, lysates were prepared through repeated freeze—thawing of the cells. Lysates were stored at −80°C until use.

### Cytofluorimetric analyses of MAIT cell phenotypes and functions

Human bulk PBMCs, HMNCs, or fluorescence-activated cell sorting (FACS)-purified CD3^+^Vα7.2^+^CD161^high^ cells or cell subsets were left untreated or stimulated with stated doses of indicated SAgs, with a 1:5 dilution of *K*. *pneumoniae* lysate or a 1:2 dilution of other bacterial lysates, with 5 ng/mL of rIL-12 (PeproTech) and/or 5 ng/mL of rIL-18 (R&D Systems, Minneapolis, MN), with 100 ng/mL of LPS from *E*. *coli* 0111:B4 (Sigma-Aldrich SKU # L4391), or with a combination of 100 ng/mL of SEB and 100 ng/mL of LPS.

Various experimental designs required the blockade of MR1, HLA-DR, or LAG-3 by the addition of 5 μg/mL of a LEAF-purified anti-human/mouse/rat MR1 mAb (clone 26.5, BioLegend, San Diego, CA), 5 μg/mL of a mouse anti-HLA-DR mAb (clone G46-6, BD Biosciences, San Jose, CA), or 20 μg/mL of a mouse anti-human LAG-3 mAb (clone 17B4, Adipogen, San Diego, CA), respectively. IFN-γ, IL-12, and/or IL-18 were neutralized using 5 μg/mL of NIB42 (eBioscience, San Diego, CA), B-T21 (eBioscience), and/or 125-2H (R&D Systems) mAbs, respectively. SB203580 (a selective inhibitor of p38 MAP kinase) and PD98059 [a MAPK/ERK kinase (MEK) 1/2 inhibitor] were purchased from Sigma-Aldrich, dissolved at 1 mg/mL DMSO, and used at a 20-μM final concentration in cultures. Finally, 200 ng/mL of the NFAT inhibitor CsA (Focus Biomolecules, Plymouth Meeting, PA) was present in some cultures.

Freshly isolated, untreated, or stimulated cells were washed and stained at 4°C with fluorochrome-conjugated mAbs to cell surface CD3, CD14, CD69, CD161, CD212 (IL-12Rβ1), CD218a (IL-18Rα), CD223 (LAG-3), CD279 (PD-1), CD366 (TIM-3), TCR Vα7.2, TCR Vβ2, TCR Vβ13.2, and/or TCR γδ (S1 Table), which were diluted in 2% FCS in cold PBS. After 30 min, cells were thoroughly washed, interrogated using a BD FACSCanto II flow cytometer, and analyzed by FlowJo software (Tree Star, Ashland, OR). *i*NKT cells were identified through co-staining with an anti-CD3 mAb and allophycocyanin-conjugated, PBS-57-loaded human CD1d tetramers generously supplied by the NIH Tetramer Core Facility (Atlanta, GA). For intracellular detection of IFN-γ, IL-2, IL-17A, TNF-α, CD107a, granzymes (GZM) A and B, T-bet, RORγt, or phospho-p38, a combination of 1 μM brefeldin A (Sigma-Aldrich) and 2 μM monensin (eBioscience) was added to the cells either at the beginning of the short-term cultures or during the final 5 h. When cell surface and intracellular staining needed to be combined, cells were first stained with mAbs to surface molecules, washed, resuspended in Intracellular Fixation & Permeabilization Buffer Set (eBioscience), and kept in the dark for 20 min at room temperature. In the case of transcription factors, a FoxP3 Staining Buffer Set (eBioscience) was used. Cells were subsequently washed and stained with mAbs against indicated intracellular molecules (S1 Table).

To detect cell death and early apoptosis, cells were first stained with a 1:100 dilution of Fixable Viability Dye eFluor 780 (eBioscience) in PBS to allow for dead cell exclusion. Annexin V^+^ cells were then identified using an Apoptosis Detection kit from eBioscience.

To confirm successful humanization of NSG mice, their splenic cells were stained with anti-human CD45 and anti-human CD3 mAbs (S1 Table).

For detection of mouse MAIT cells, MR1 tetramer reagents were assembled, labeled, and used [18,19]. In brief, Phycoerythrin (PE) Streptavidin (BD Biosciences) was added at 10-min intervals to biotinylated mouse MR1 monomers at room temperature. The resulting tetramerized MR1 molecules were stored at 4°C in the dark until use. Non-parenchymal lung MNCs were prepared after digestion of lung tissue homogenate with 0.5 mg/mL of collagenase type IV (Sigma-Aldrich) in RPMI medium for 1 h at 37°C. Cells were then gently forced through a 70-μm filter, washed, spun, erythrocyte-depleted, washed, and filtered again and incubated with a 1:400 dilution of 5-OP-RU-loaded MR1 tetramer in PBS containing 2% FCS for 30 min at room temperature. This step was followed by staining at 4°C with either a FITC-conjugated anti-mouse TCRβ mAb (clone H57-597) or with an allophycocyanin-conjugated rat anti-mouse CD3ε mAb (clone 17A2), along with fluorochrome-labeled mAbs to mouse PD-1 (clone J43), LAG-3 (clone eBioC9B7W), and TIM-3 (clone RMT3-23) (S1 Table). Finally, cells were stained with Fixable Viability Dye eFluor 780 (eBioscience) to allow for dead cell exclusion. Cells were subsequently washed and analyzed by flow cytometry. PE-conjugated, 6-FP-loaded MR1 tetramers served as a staining control in these experiments.

In all flow cytometry experiments, isotype controls corresponding to fluorochrome-labeled mAb were used in parallel to allow for appropriate gating.

### Cytokine measurements

Mouse MAIT hybridomas were seeded at 1 × 10^5^ cells/250 μL complete medium/well of a U-bottom polystyrene microplate along with 2 × 10^4^ B6 or DR4 tg BMDCs. Cultures were stimulated with indicated SAgs at a final concentration of 100 ng/mL, with 0.5 μg/mL of a hamster anti-mouse CD3ε mAb (clone 145-2C11 from Cedarlane Labs, Burlington, ON), or with a 1:5 dilution of *K*. *pneumoniae* lysate. In several experiments, γ-irradiated (3,000 rad) MAIT cells or BMDCs were utilized, and blockade of MR1 was achieved by the addition of 5 μg/mL of 26.5 (BioLegend). The IL-2 content of culture supernatants was quantified after 24 h by an ELISA kit from eBioscience.

Bulk human PBMCs were stimulated with 100 ng/mL of SEB for 2 h, 6 h, 12 h, and 24 h before culture supernatant samples were collected and analyzed by bead-based multiplexing (Eve Technologies, Calgary, AB). Heat maps for indicated cytokines were generated using GraphPad Prism 7 software.

CD3^+^Vα7.2^+^CD161^high^ human MAIT cells and CD3^+^Vα7.2^-^ T_conv_ cells were purified from human PBMCs using a BD FACSAria III sorter. In a limited number of experiments, concomitant staining for Vβ2 and Vβ13.2 was performed to enable sorting of MAIT cell subpopulations bearing these TCR Vβs. The purity of the sorted populations was always greater than 95%. In a microplate, 1 × 10^5^ bulk MAIT cells or T_conv_ cells, or 4 × 10^4^ Vβ2^+^ or Vβ13.2^+^ MAIT cells were co-cultured for up to 12 h with 2 × 10^4^ FACS-purified, autologous CD3^-^CD14^+^ monocytes in the absence or presence of 100 ng/mL SEB. Human IFN-γ, IL-17A, IL-12p70 and IL-18 levels were then determined in culture supernatants as indicated.

Human TNF-α was quantified by ELISA in supernatant samples harvested 24 h after THP-1 cells were stimulated with 100 ng/mL of LPS in the presence or absence of 100 μg/mL of polymyxin B.

Seven days after NSG mice received human PBMCs, they were injected with 100 μg SEB i.p. Animals were bled 24 h later, and circulating levels of human IFN-γ were quantitated by ELISA.

## Statistical analyses

Statistical assessments were made with the aid of GraphPad Prism software. Comparisons were performed using Student *t*-test or ANOVA, as appropriate, and differences with *p* < 0.05 were deemed significant. *, **, ***, and **** denote *p* < 0.05, *p* < 0.01, *p* < 0.001, and *p* < 0.0001, respectively. Association analyses were conducted by the non-parametric Spearman’s rank correlation test.

## Supporting information

S1 Table Antibodies used in this study. (DOCX)

S1 Data Raw data for all figures and supplemental figures. (XLSX)

S2 Data Cytofluorimetric gating strategies for main and supplemental figures. (PDF)

S1 Fig LPS does not affect SEB-induced MAIT cell activation. Human PBMCs from 6 healthy donors were left untreated or stimulated with 100 ng/mL of SEB in the presence or absence of 100 ng/mL of LPS or 100 μg/mL of polymyxin B as indicated. Twenty-four h later, the frequency of IFN-γ^+^ MAIT cells was determined by flow cytometry. Error bars represent SEM (A). THP-1 human monocytic cells were exposed to LPS for 24 h in the presence or absence of 100 μg/mL of polymyxin B, followed, 24 h later, by quantification of TNF-α in culture supernatant samples by ELISA. Error bars represent SD from triplicate culture wells (B).
(TIFF)

S2 Fig SEB-triggered CD69 upregulation is slightly delayed in the TCRVβ2^+^ subset of MAIT cells. Human PBMCs from 3 donors were exposed to 100 ng/mL of SEB, and the expression of CD69 on TCRVβ13.2^+^ and TCRVβ2^+^ MAIT cell subsets was assessed at indicated time points by flow cytometry. Error bars represent SEM.
(TIFF)

S3 Fig SEB stimulation does not raise the expression of CD218a in peripheral blood T cells. Human PBMCs (n = 3) were left untreated or stimulated with 100 ng/mL of SEB for indicated durations. The percentage of CD218a^+^ cells among unfractionated T cells (A) and the mean fluorescence intensity (MFI) of CD218a staining (B) were determined by flow cytometry.
(TIFF)

S4 Fig Most conventional T cells do not express CD218a or CD212 in their resting state or following SEB stimulation. Freshly isolated and SEB-stimulated human PBMCs (n = 7) were analyzed by flow cytometry to determine the frequencies of CD218a^+^ and CD212^+^ cells among CD3^+^Vα7.2^-^ T_conv_ cells. Filled and open histograms (left panel) correspond to staining with isotype controls and anti-CD218a/CD212, respectively. Each circle represents an individual in the right panel where error bars represent SEM.
(TIFF)

S5 Fig TCRVβ13.2^+^ T_conv_ cells mount a modest IFN-γ response to SEB that is IL-12/IL-18-independent. Human PBMCs (n = 6) were stimulated with 100 ng/mL of SEB in the presence of IL-12- and/or IL-18-neutralizing mAbs or an isotype control. Twenty-four h later, the frequency of IFN-γ^+^ cells among TCRVβ13.2^+^ T_conv_ cells was determined by flow cytometry. Error bars represent SEM.
(TIFF)

S6 Fig Endogenous IFN-γ is dispensable for SEB-induced cytokine production by MAIT cells. Human PBMCs (n = 4) were stimulated with SEB in the presence of an anti-IFN-γ mAb or isotype control. Twenty-four h later, the frequency of IFN-γ-, TNF-α- and IL-2-producing MAIT cells was determined by flow cytometry. Error bars represent SEM.
(TIFF)

S7 Fig MAIT cells respond more vigorously to SEB than against gram-negative bacteria. Human PBMCs (n = 7) were left untreated or exposed to SEB, a combination of rIL-12 and rIL-18, or bacterial cell lysates prepared from *K*. *pneumoniae*, *E*. *coli*, *P*. *aeruginosa* or *S*. *typhimurium*. Twenty-four h later, the percentage of IFN-γ-producing MAIT cells were calculated. Error bars represent SEM.
(TIFF)

S8 Fig *i*TCR-dependent stimulation of MAIT cells renders them unresponsive to a subsequent *i*TCR-based challenge. Human PBMCs (n = 13) were left untreated or subjected to stimulation with *K*. *pneumoniae* lysate or a combination rIL-12 and rIL-18. Twenty-four h later, cells were washed and rested for an additional 24 h before they were left in complete medium or challenged with SEB or *K*. *pneumoniae* lysate as indicated. This was followed, 24 h later, by cytofluorimetric calculation of IFN-γ^+^ MAIT cell frequencies. Error bars represent SEM.
(TIFF)

S9 Fig Wild-type CAST/EiJ mice are responsive to SEB. In a pilot experiment, one CAST/EiJ mouse was injected with sterile PBS and another mouse received a 100-μg *i*.*p*. injection of SEB. Twelve h later, serum IFN-γ levels were quantitated by ELISA (A). In addition, 4 days after PBS/SEB injection, splenic, hepatic and lung non-parenchymal mononuclear cells (MNCs) were enumerated (B).
(TIFF)

S10 Fig Unlike CD3/CD28 co-ligation, SEB stimulation does not result in *i*TCR downregulation in human MAIT cells. PBMCs (n = 3) were cultured in the absence or presence of SEB (100 ng/mL) or a combination of agonistic anti-human CD3 (clone OKT3) and anti-human CD28 (clone 9.3) mAbs, each of which was used at 0.5 μg/mL. Twenty-four h later, the presence and the frequency, when applicable, of Vα7.2^+^CD161^high^ MAIT cells were assessed by flow cytometry (A). The extent of *i*TCR internalization, or lack thereof, was determined at indicated time points through step-wise staining of SEB-exposed cells with Vα7.2 mAbs labeled with two different fluorochromes. Cells were stained for surface *i*TCR before they were washed, fixed, permeablized and stained for intracellular *i*TCR. Representative data from one donor are illustrated.
(TIFF)

S11 Fig SEB-induced IFN-γ production by T_conv_ cells can be equally inhibited by IL-12 and IL-18 neutralization. PBMCs (n = 7) were stimulated with SEB in the presence of anti-human IL-12, anti-human IL-18 or isotype control. Twenty-four h later, the frequency of IFN-γ^+^ T_conv_ was determined by flow cytometry. Error bars represent SEM.
(TIFF)

S12 Fig SEB stimulation increases the frequencies of potentially cytotoxic MAIT cells in an IL-12/IL-18-dependent fashion. Human PBMCs (n = 7–8) were stimulated with SEB for indicated durations, and the percentages of cells co-expressing CD107a and/or granzymes A/B were determined among T_conv_ (A) and MAIT cells (B). For five samples, a combination of anti-IL-12 and IL-18 (or isotype control) was present in cultures (C). Error bars represent SEM.
(TIFF)

## Abbreviations

Ag: antigen
AP-1: activator protein 1
BMDC: bone marrow-derived dendritic cell
CsA: cyclosporine A
DN: double negative
DR4 tg: DR4-transgenic
FACS: fluorescence-activated cell sorting
HMNC: hepatic mononuclear cell
hPBMC-NSG: human PBMC-reconstituted NSG
i.p.: intraperitoneal
ICU: intensive care unit
IFN: interferon
IL: interleukin
*i*NKT: invariant natural killer T cell
*i*TCRα: invariant T cell receptor α
LAG-3: lymphocyte-activation gene 3
LPS: lipopolysaccharide
mAb: monoclonal antibody
MAIT: mucosa-associated invariant T cell
MAPK: Mitogen-activated protein kinase
MDSC: myeloid-derived suppressor cell
MFI: mean fluorescence intensity
MHC: major histocompatibility complex
MNC: mononuclear cell
MR1: MHC-related protein 1
NSG: NOD-*scid* IL-2Rγ^null^
PBMC: peripheral blood mononuclear cell
PD-1: programmed cell death-1
PMA: phorbol 12-myristate 13-acetate
rIL-12: recombinant human IL-12
rIL-18: recombinant human IL-18
SAgs: superantigens
SEA: staphylococcal enterotoxin A
SEB: staphylococcal enterotoxin B
Spe: streptococcal pyrogenic exotoxin
STAT: signal transducer and activator of transcription
T_CM_: central memory T cell
T_conv_: conventional T cell
TCR: T cell receptor
T_EM_: effector memory T cell
TIM-3: T cell immunoglobulin and mucin-3
TLR: Toll-like receptor
TNF: tumor necrosis factor
Treg: regulatory T cell
TSS: toxic shock syndrome
TSST-1: toxic shock syndrome toxin-1
