# Supplementary material for: MAIT cells launch a rapid, robust and distinct hyperinflammatory response to bacterial superantigens and quickly acquire an anergic phenotype that impedes their cognate antimicrobial function: Defining a novel mechanism of superantigen-induced immunopathology and immunosuppression
Source: PLoS Biol. 2017 Jun 20;15(6):e2001930. doi: 10.1371/journal.pbio.2001930 (PMC5478099; doi:10.1371/journal.pbio.2001930)
Supplement: S1 Table — (DOCX) [file pbio.2001930.s001.docx]

| Marker | Reactivity | Label | Clone | Isotype | Host species | Source | |
| --- | --- | --- | --- | --- | --- | --- | --- |
| *Surface Markers* |  |  |  |  |  |  | |
| CCR7 | Human | PE-eFluor 610 | 3D12 | IgG2a, κ | Rat | eBioscience |  |
| CD3 | Human | FITC | HIT3a | IgG2a, κ | Mouse | eBioscience |  |
| CD3 | Human | APC-eFluor 780 | UCHT1 | IgG1, κ | Mouse | eBioscience |  |
| CD4 | Human | Alexa Fluor 700 | RPA-T4 | IgG1, κ | Mouse | eBioscience | |
| CD8 | Human | FITC | SK1 | IgG1, χ | Mouse | BD Biosciences | |
| CD14 | Human | eVolve 605 | 61D3 | IgG1, κ | Mouse | eBioscience | |
| CD45RO | Human | APC | UCHL1 | IgG2a, κ | Mouse | eBioscience | |
| CD69 | Human | PeCy7 | FN50 | IgG1, κ | Mouse | eBioscience | |
| CD161 | Human | APC | HP-3G10 | IgG1 | Mouse | eBioscience | |
| CD212 | Human | PE | 2.4E6 | IgG1, κ | Mouse | BD Biosciences | |
| CD218a | Human | APC | H44 | IgG1, κ | Mouse | eBioscience | |
| CD223 (LAG-3) | Human | PE-eFluor 610 | 3DS223H | IgG1, κ | Mouse | eBioscience | |
| CD279 (PD-1) | Human | APC-eFluor 780 | eBioJ105 | IgG1, κ | Mouse | eBioscience | |
| CD366 (TIM-3) | Human | PE | F38-2E2 | IgG1, κ | Mouse | eBioscience | |
| HLA-DR | Human | PE | G46-6 | IgG2a, κ | Mouse | BD Biosciences | |
| TCR Vα7.2 | Human | PercpCy5.5 | [3C10](http://www.biolegend.com/index.php?page=pro_sub_cat&action=search_clone&criteria=3C10) | IgG1, κ | Mouse | BioLegend | |
| TCR Vβ2 | Human | FITC | MPB2D5 | IgG1 | Mouse | Beckman Coulter | |
| TCR Vβ13.2 | Human | PE | H132 | IgG1 | Mouse | Beckman Coulter | |
| CD3 | Mouse | APC | 17A2 | IgG2b, κ | Rat | eBioscience | |
| CD223 (LAG-3) | Mouse | PerCP-eFluor 710 | eBioC9B7W | IgG1, κ | Rat | eBioscience | |
| CD279 (PD-1) | Mouse | PE-eFluor 610 | J43 | IgG | Armenian Hamster | eBioscience | |
| CD366 (TIM-3) | Mouse | PeCy7 | RMT3-23 | IgG2a, κ | Rat | eBioscience | |
| TCRβ | Mouse | FITC / PE / APC | H57-597 | IgG | Armenian Hamster | eBioscience | |
| TCR Vβ8.1/82 | Mouse | FITC | KJ16-133 | IgG2a, κ | Rat | eBioscience | |
| *Intracellular Molecules* |  |  |  |  |  |  | |
| CD107a (LAMP-1) | Human | APC/Cy7 | H4A3 | IgG1, κ | Mouse | BioLegend | |
| Granzyme A | Human | Alexa Fluor 700 | CB9 | IgG1, κ | Mouse | BioLegend | |
| Granzyme B | Human | PE | GB11 | IgG1 | Mouse | eBioscience | |
| Granzyme K | Human | PerCP-eFluor 710 | G3H69 | IgG2a, κ | Mouse | eBioscience | |
| IL-2 | Human | PE-eFluor 610 | MQ1-17H12 | IgG2a, κ | Rat | eBioscience | |
| IL-17A | Human | PeCy7 | eBio64DEC17 | IgG1, κ | Mouse | eBioscience | |
| IFN-γ | Human | APC-eFluor 780 / PeCy7/ PE | 4S.B3 | IgG1, κ | Mouse | eBioscience | |
| Phospho-p38 MAPK (T180/Y182) | Human/Mouse | PE / PECy7 | 4NIT4KK | IgG2b, κ | Mouse | eBioscience | |
| RORγt | Human | APC | AFKJS-9 | IgG2a | Rat | eBioscience | |
| TNF-α | Human | PE | MAb11 | IgG1, κ | Mouse | eBioscience | |
| T-bet | Human | PE | O4-46 | IgG1, κ | Mouse | BD Biosciences | |
| *Blocking/Neutralizing mAbs* |  |  |  |  |  |  | |
| HLA-DR | Human | Purified | G46-6 | IgG2a, κ | Mouse | BD Biosciences | |
| IL-12 | Human | Purified | B-T21 | IgG1 | Mouse | eBioscience | |
| IFN-γ | Human | Purified | NIB42 | IgG1, κ | Mouse | eBioscience | |
| IL-18/IL1F4 | Human | Purified | 125-2H | IgG1 | Mouse | R&D Systems | |
| LAG-3 | Human | Purified | 17B4 | IgG1 | Mouse | Adipogen | |
| MR1 | Human/Mouse/Rat | Purified | 26.5 | IgG2a, κ | Mouse | BioLegend | |
|  |  |  |  |  |  |  | |
|  |  |  |  |  |  |  | |
|  |  |  |  |  |  |  | |
|  |  |  |  |  |  |  | |
|  |  |  |  |  |  |  | |
|  |  |  |  |  |  |  | |
|  |  |  |  |  |  |  | |
