# Supplementary material for: MAIT cells launch a rapid, robust and distinct hyperinflammatory response to bacterial superantigens and quickly acquire an anergic phenotype that impedes their cognate antimicrobial function: Defining a novel mechanism of superantigen-induced immunopathology and immunosuppression
Source: PLoS Biol. 2017 Jun 20;15(6):e2001930. doi: 10.1371/journal.pbio.2001930 (PMC5478099; doi:10.1371/journal.pbio.2001930)
Supplement: S2 Data — (PDF) [file pbio.2001930.s003.pdf]

Cytofluorimetric Gating Strategies for Main and Supplemental Figures

Fig 1

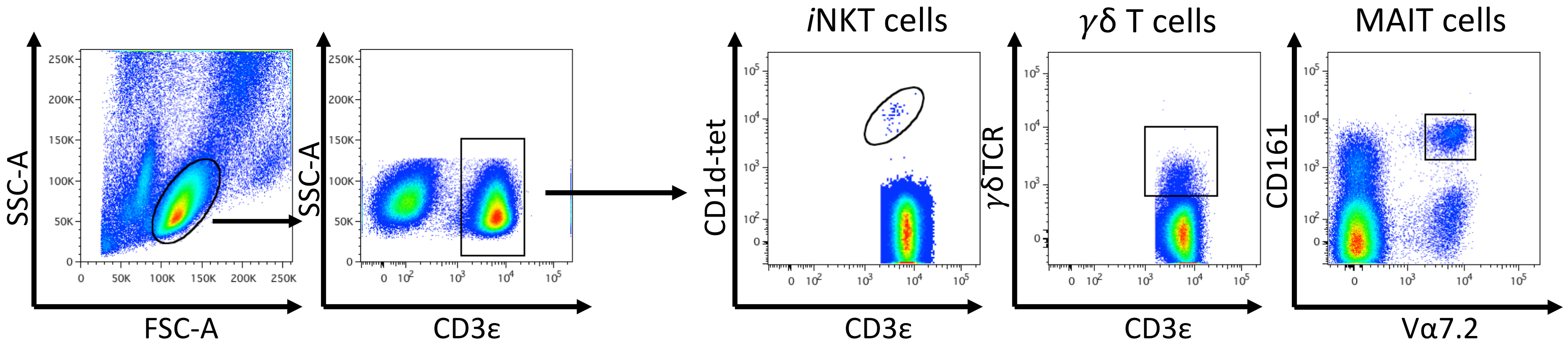

iNKT cells
γδ T cells
MAIT cells
SSC-A
FSC-A
SSC-A
CD3ε
CD1d-tet
CD3ε
γδTCR
CD3ε
CD161
Vα7.2

Fig 2a

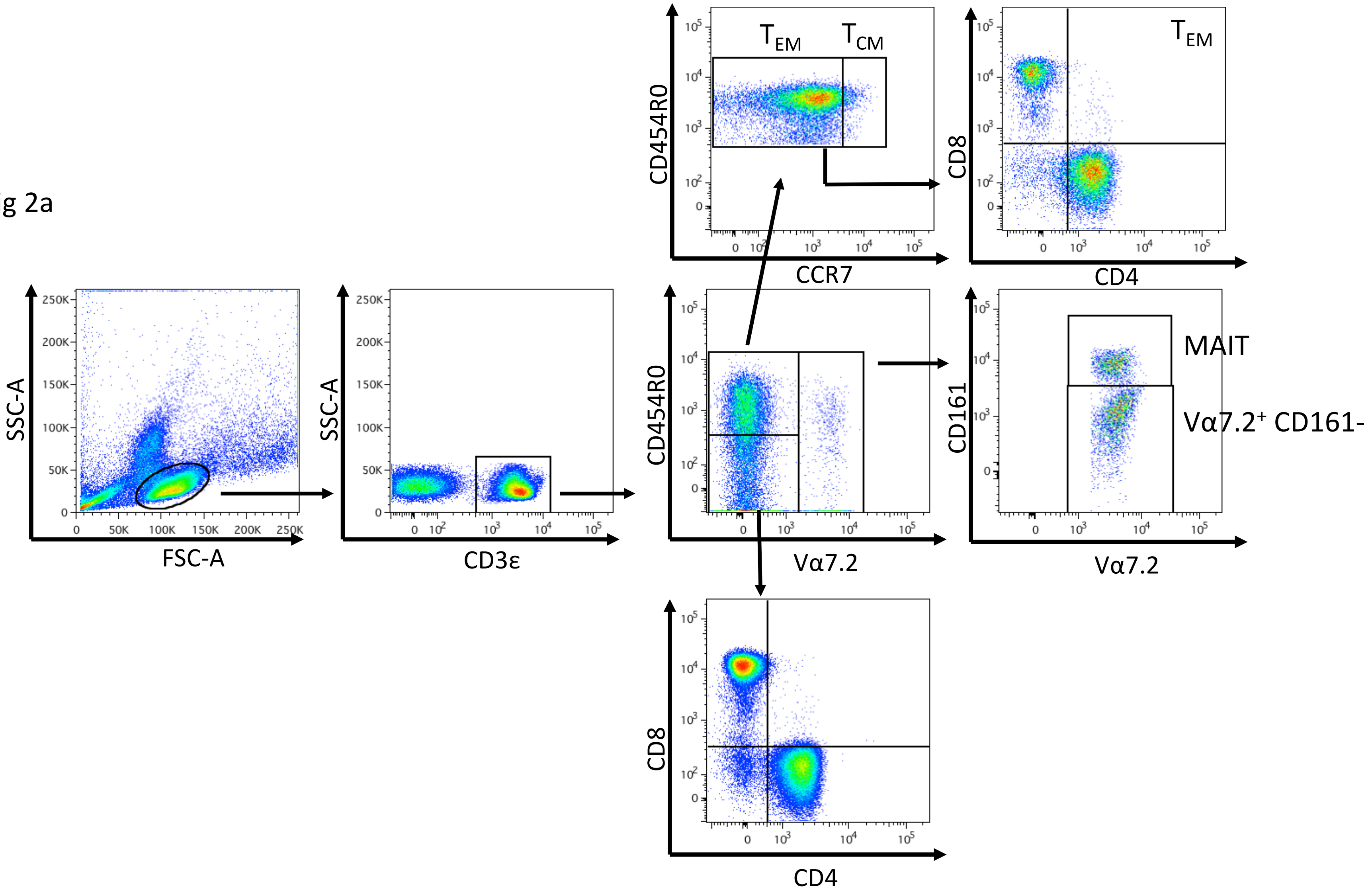
SSC-A
FSC-A
CD3ε
CD454R0
CCR7
T_{EM} T_{CM}
Vα7.2
CD8
CD4
T_{EM}
CD161
MAIT
Vα7.2+ CD161-

Fig 2A&B

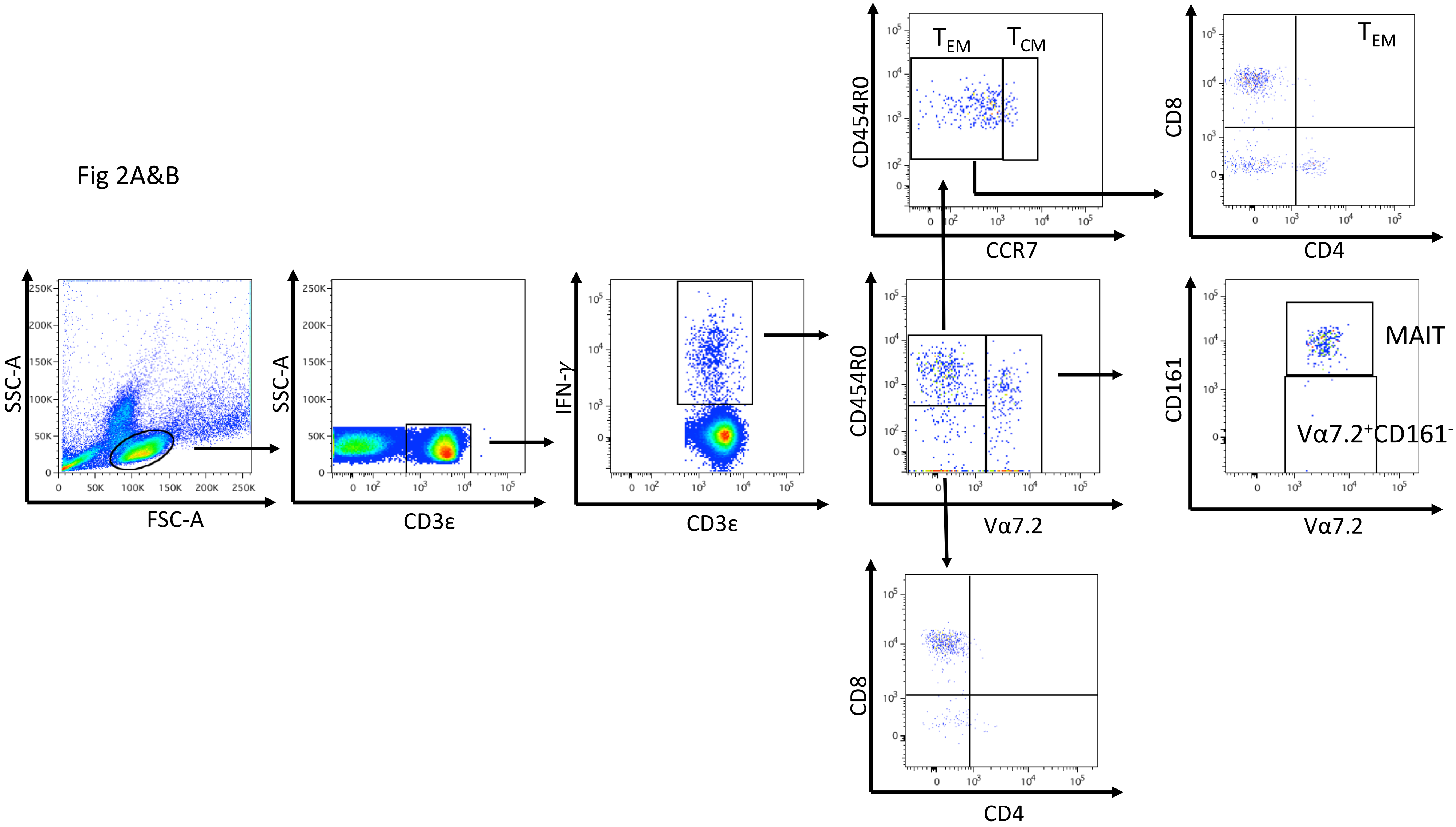
SSC-A
FSC-A
SSC-A
CD3ε
IFN-γ
CD3ε
CD454R0
Vα7.2
CD454R0
CCR7
TEM
TCM
CD8
CD4
TEM
CD161
Vα7.2
MAIT
Vα7.2+CD161-
CD8
CD4

Fig 3A&B

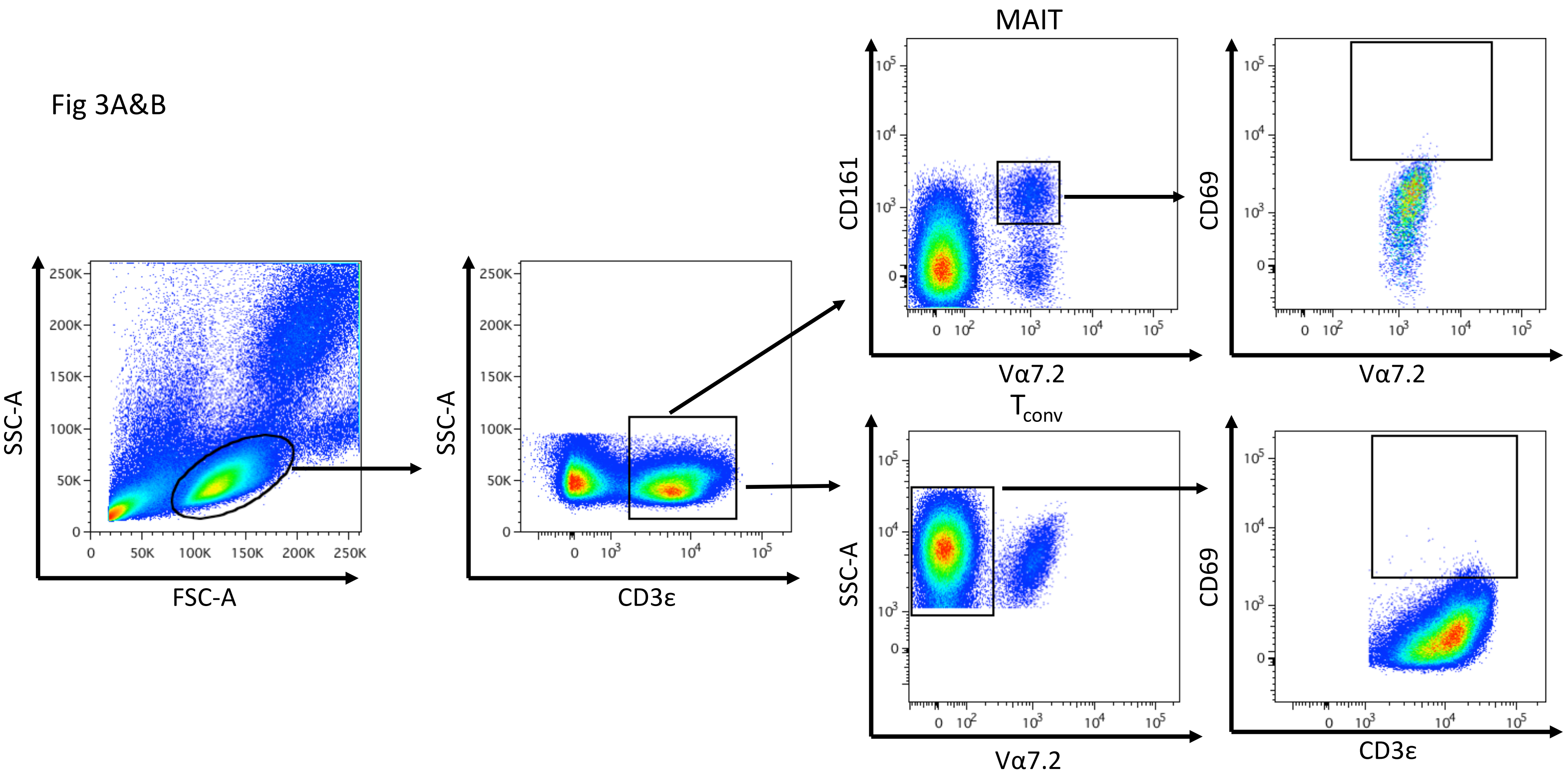

SSC-A
FSC-A
SSC-A
CD3ε
MAIT
CD161
Vα7.2
CD69
Vα7.2
Tconv
SSC-A
Vα7.2
CD69
CD3ε

Fig 3C&D

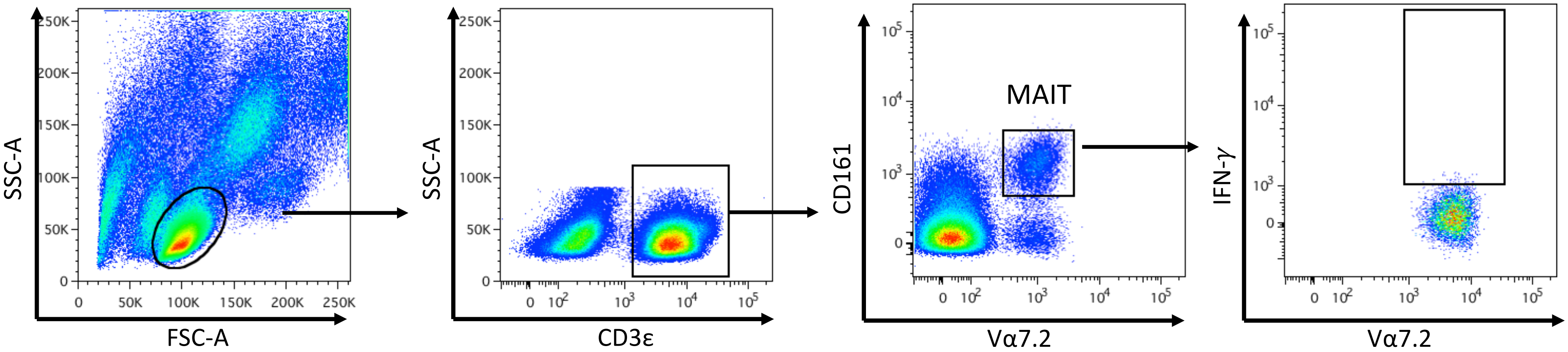

Fig 4B&E

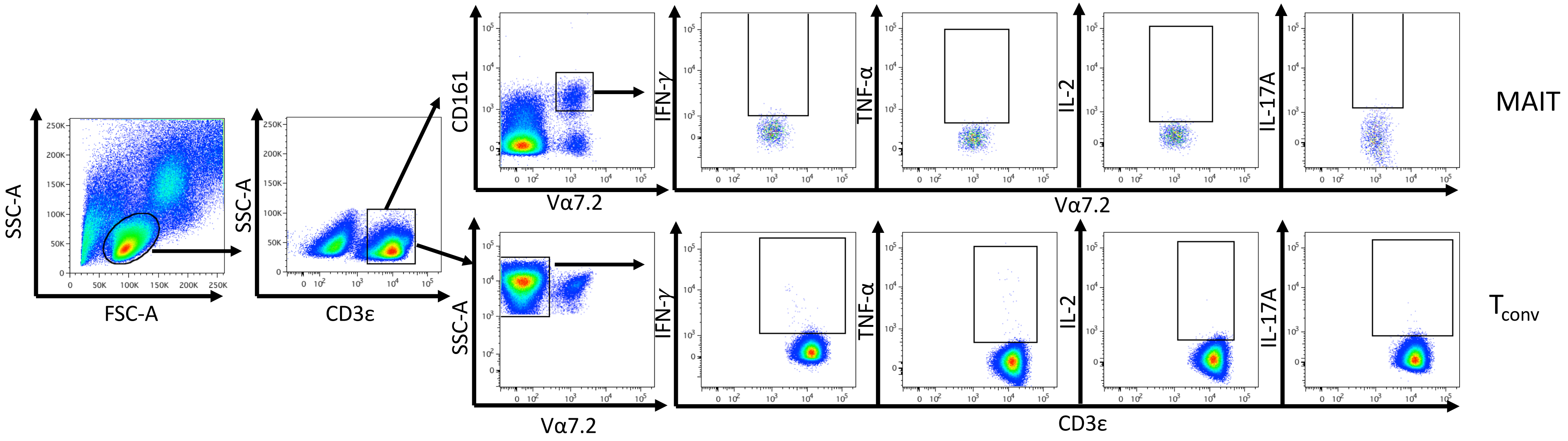

SSC-A
FSC-A
SSC-A
CD3ε
CD161
Vα7.2
SSC-A
Vα7.2
IFN-γ
TNF-α
IL-2
IL-17A
Vα7.2
CD3ε
MAIT
Tconv

Fig 4C

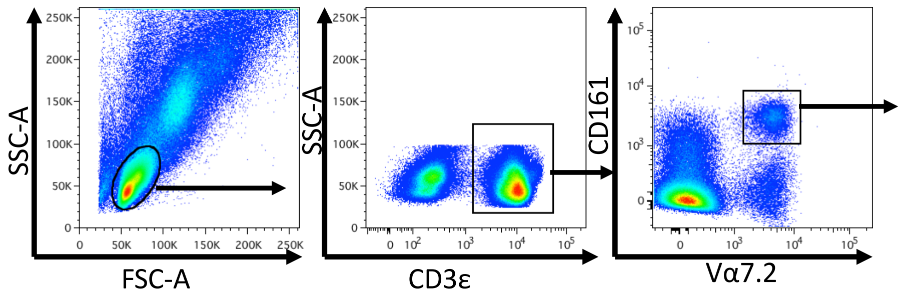

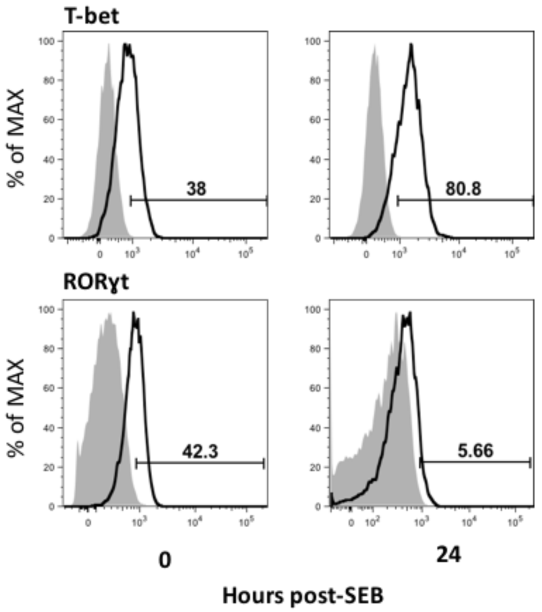

Figure 4D

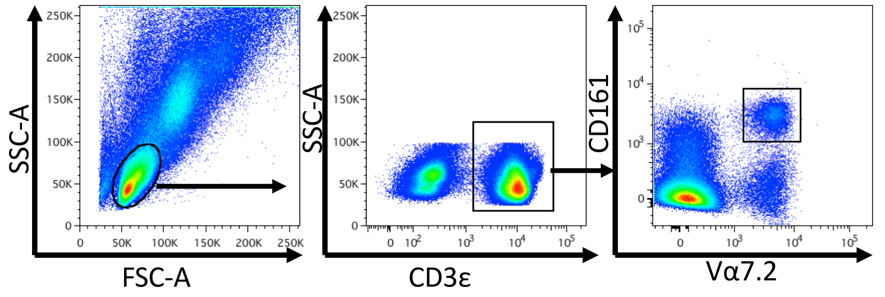

Fig 5G&H

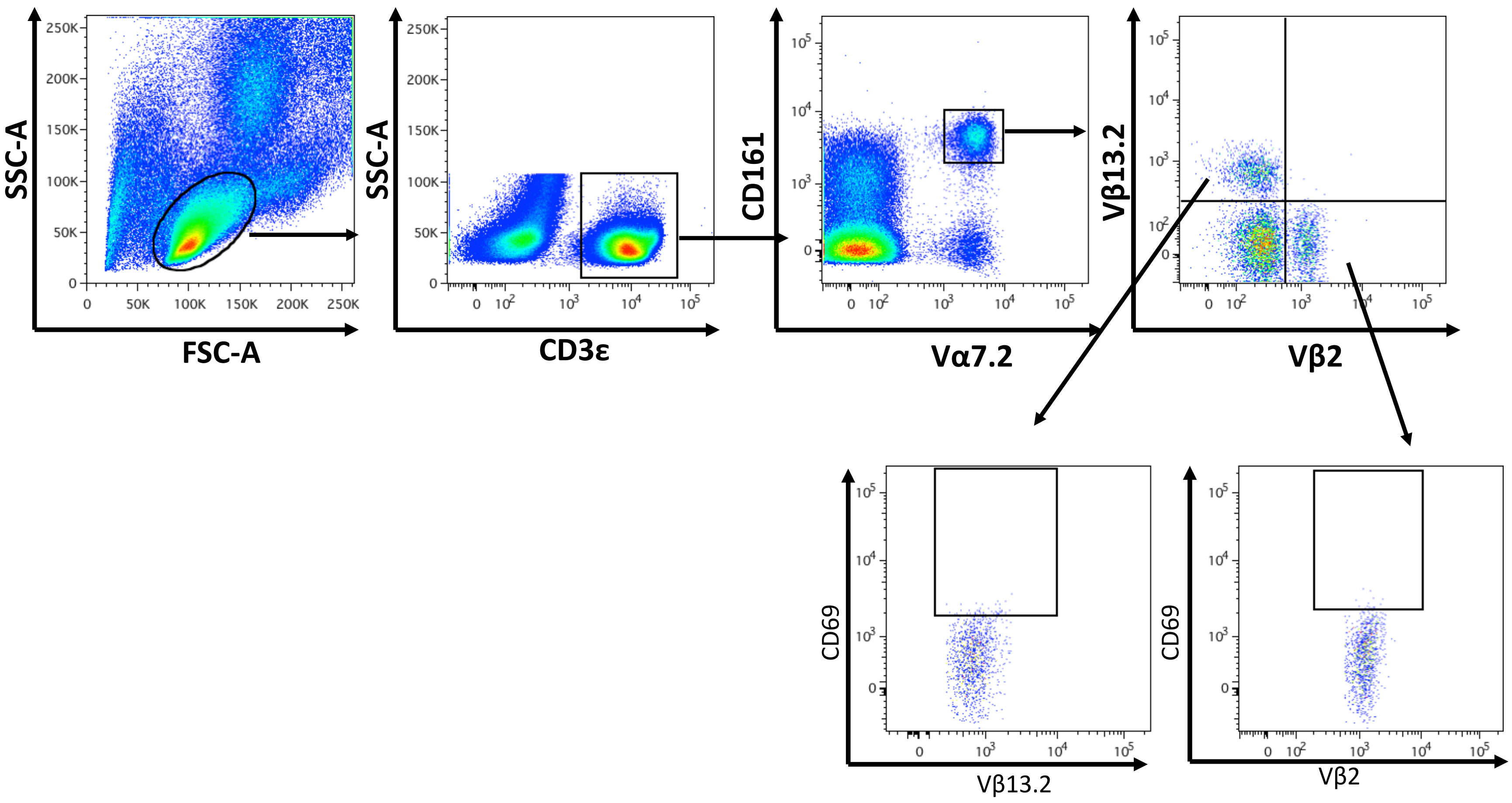

Fig 6a

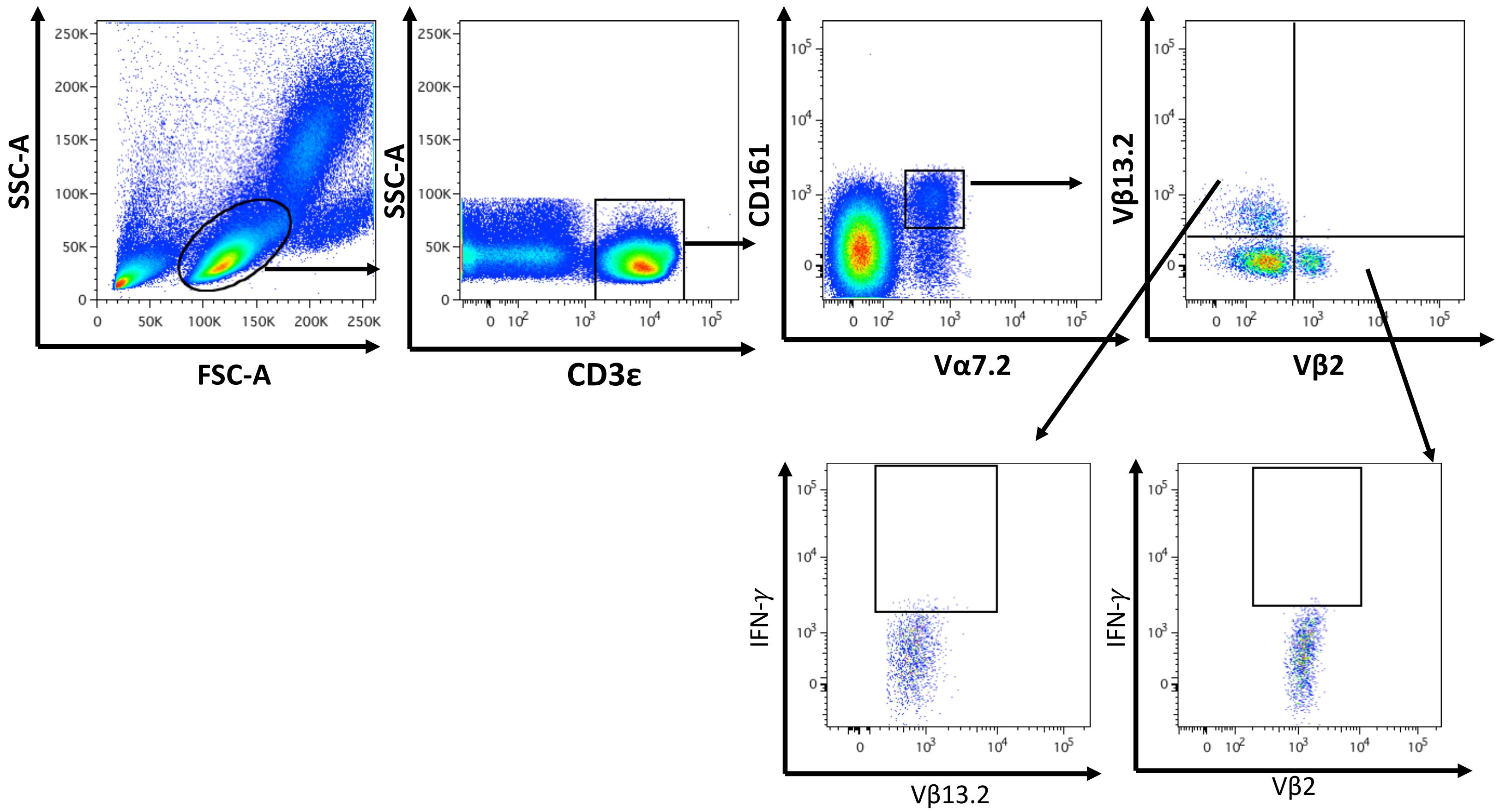

Fig 6C

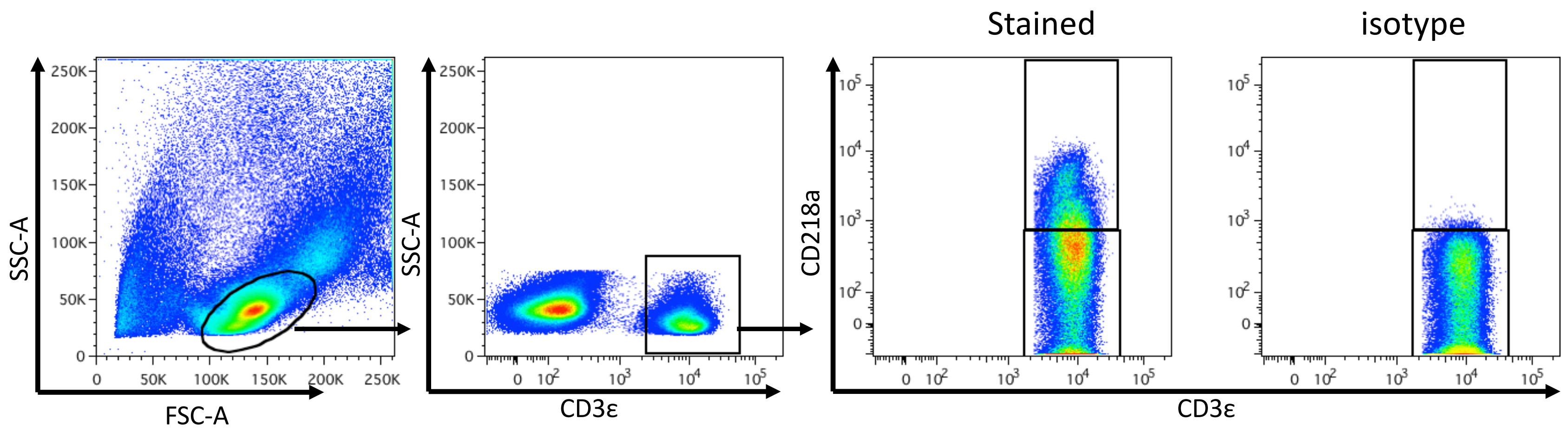

Fig 6D

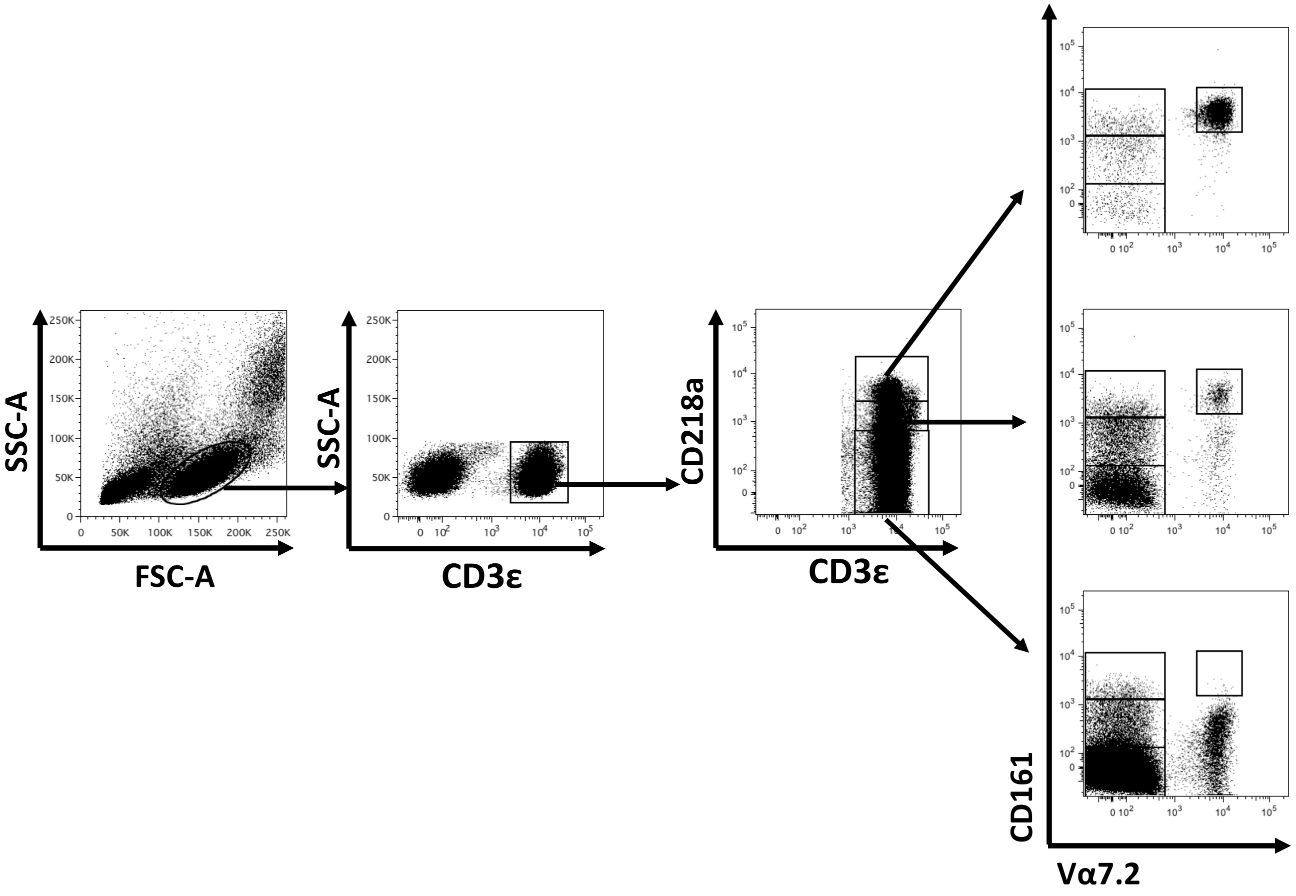

Fig 6E

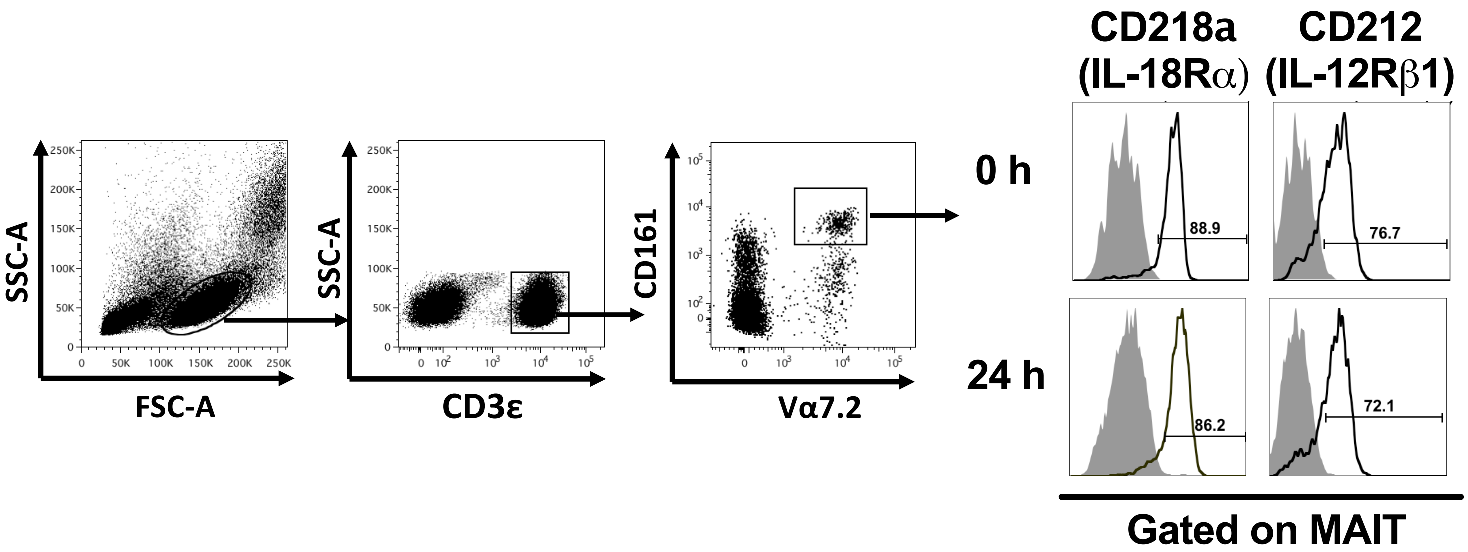

SSC-A
FSC-A
SSC-A
CD3ε
CD161
Vα7.2
CD218a (IL-18Rα)
CD212 (IL-12Rβ1)
0 h
24 h
88.9
76.7
86.2
72.1
Gated on MAIT

Fig 6F&I

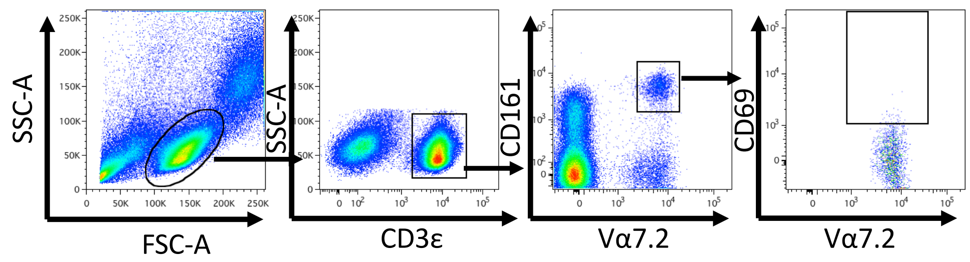

Fig 6G&I

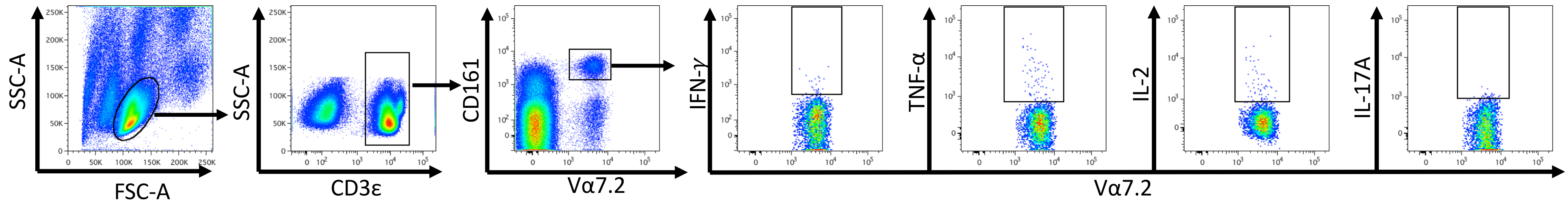

Fig 6H

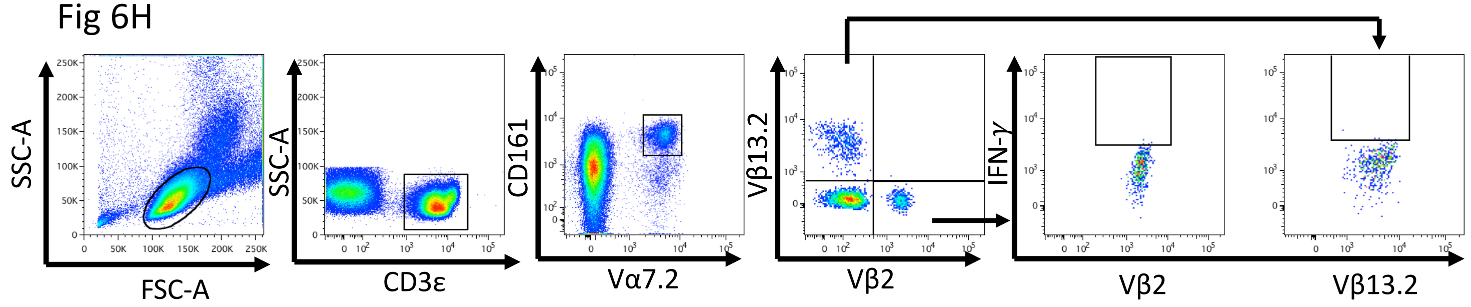

Fig 7a-c

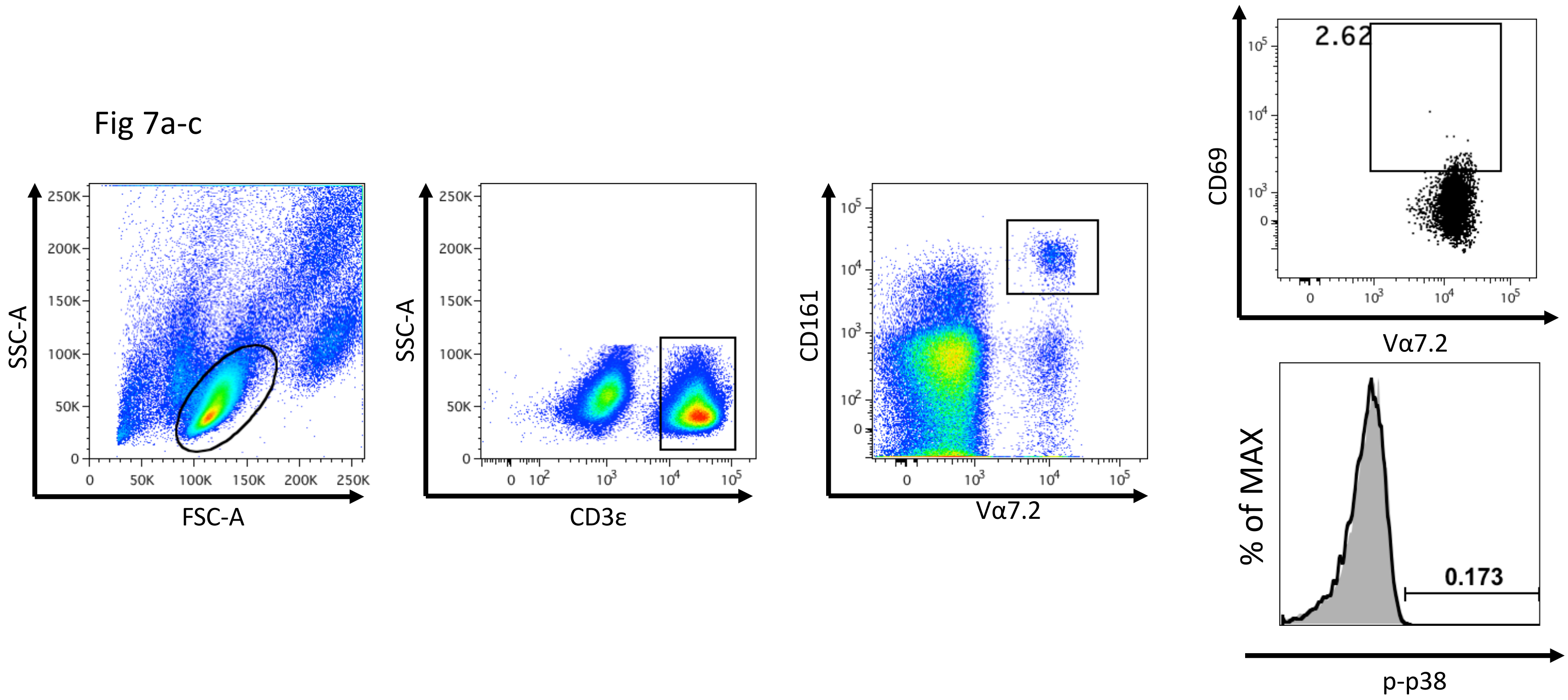

SSC-A
250K
200K
150K
100K
50K
0
0
50K
100K
150K
200K
250K
FSC-A
SSC-A
CD3ε
CD161
Vα7.2
CD69
2.62
Vα7.2
% of MAX
0.173
p-p38

Fig 8A&B

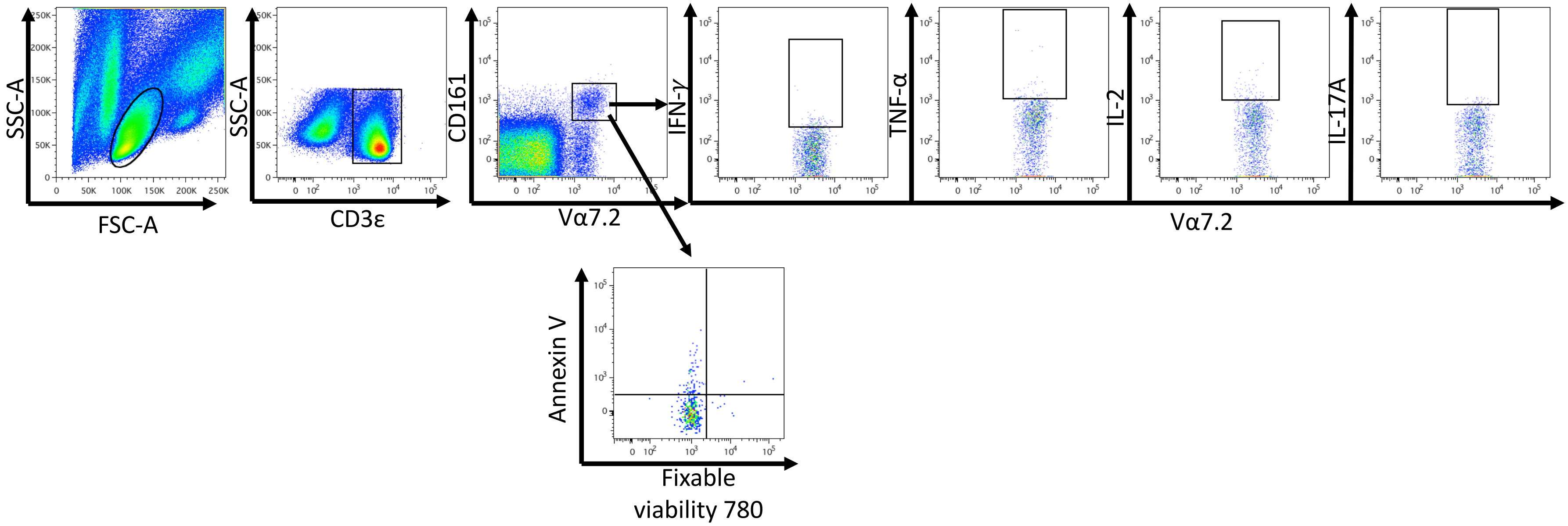
SSC-A
FSC-A
SSC-A
CD3ε
CD161
Vα7.2
IFN-γ
TNF-α
IL-2
IL-17A
Vα7.2
Annexin V
Fixable viability 780

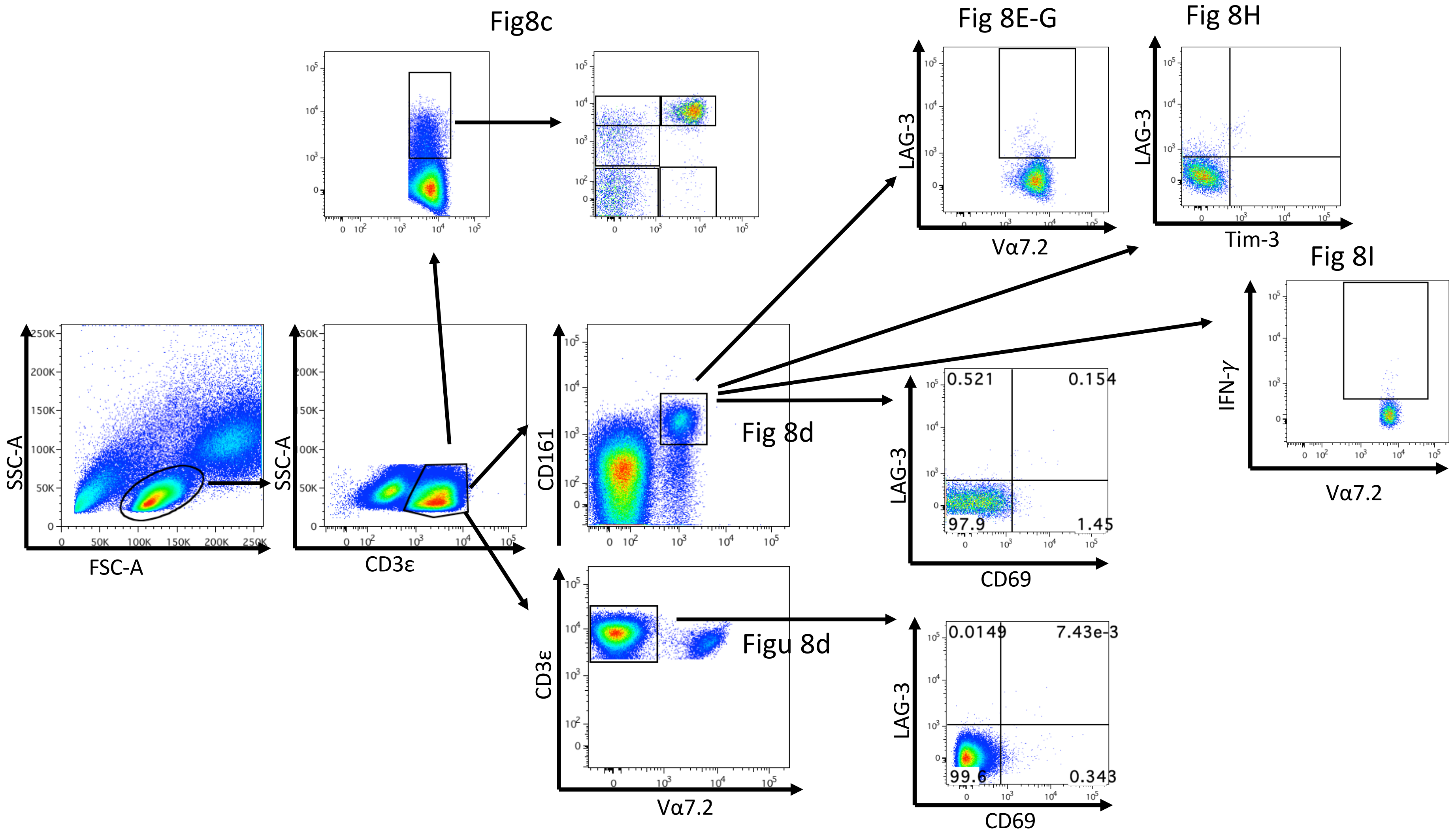

Fig8c
Fig 8E-G
Fig 8H
Fig 8I
Fig 8d
Figu 8d
SSC-A
FSC-A
CD3ε
CD161
Vα7.2
LAG-3
Tim-3
IFN-γ
CD69
0.521
0.154
97.9
1.45
0.0149
7.43e-3
99.6
0.343

Fig 9B

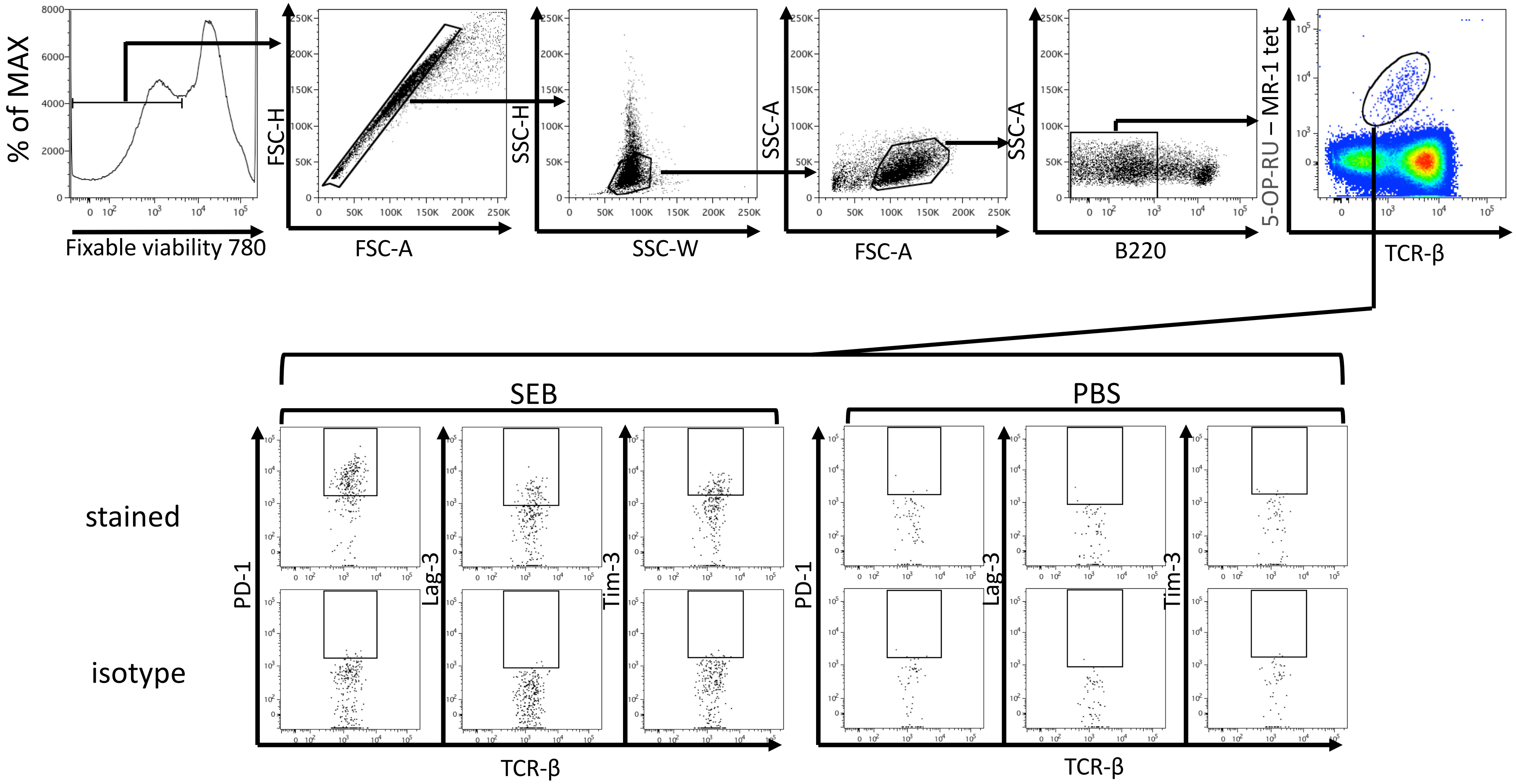
% of MAX
Fixable viability 780
FSC-H
FSC-A
SSC-H
SSC-W
SSC-A
FSC-A
SSC-A
B220
5-OP-RU – MR-1 tet
TCR-β
SEB
PBS
stained
isotype
PD-1
Lag-3
Tim-3
PD-1
Lag-3
Tim-3
TCR-β
TCR-β

Fig 10B

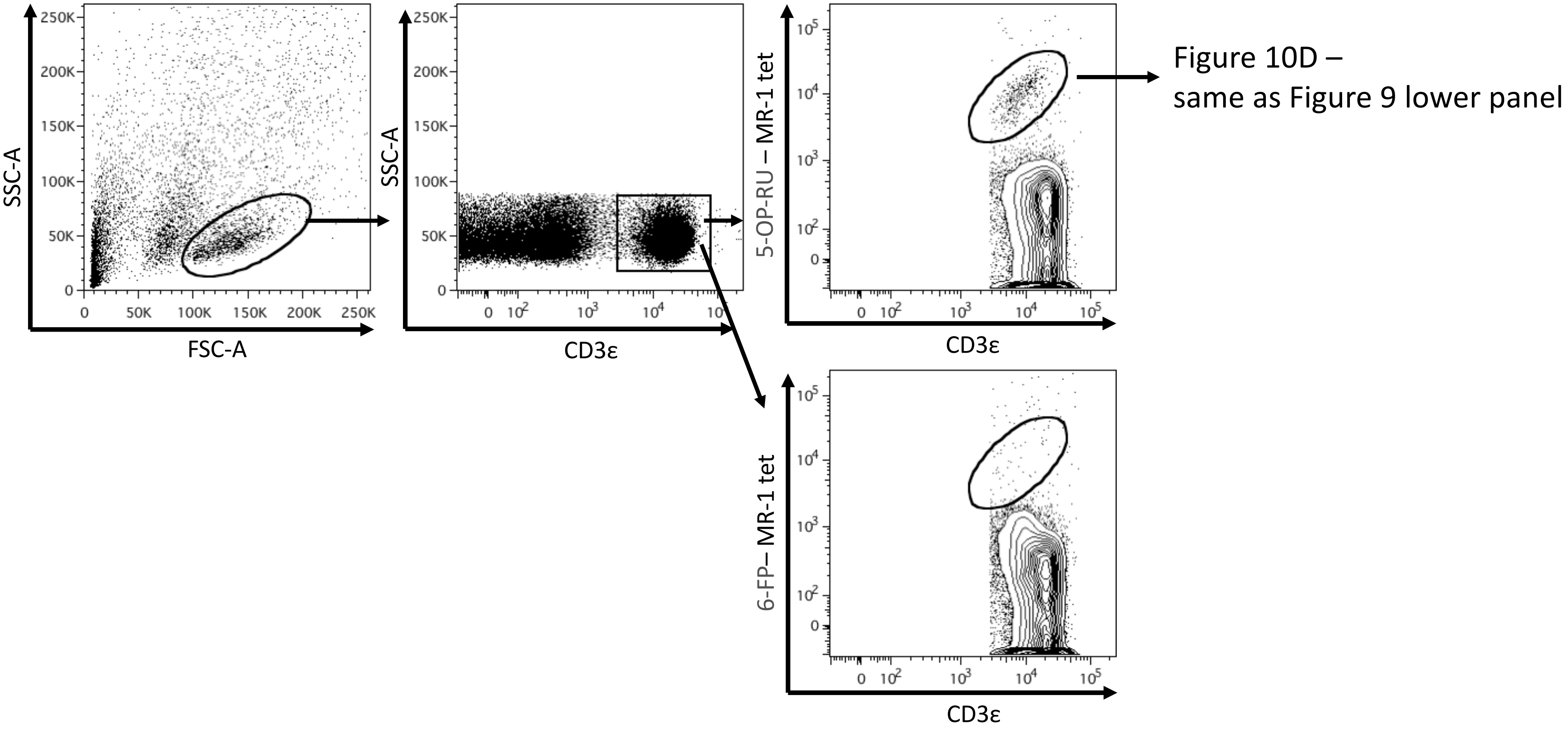
SSC-A
FSC-A
CD3ε
5-OP-RU – MR-1 tet
6-FP– MR-1 tet
Figure 10D –
same as Figure 9 lower panel

Fig 11

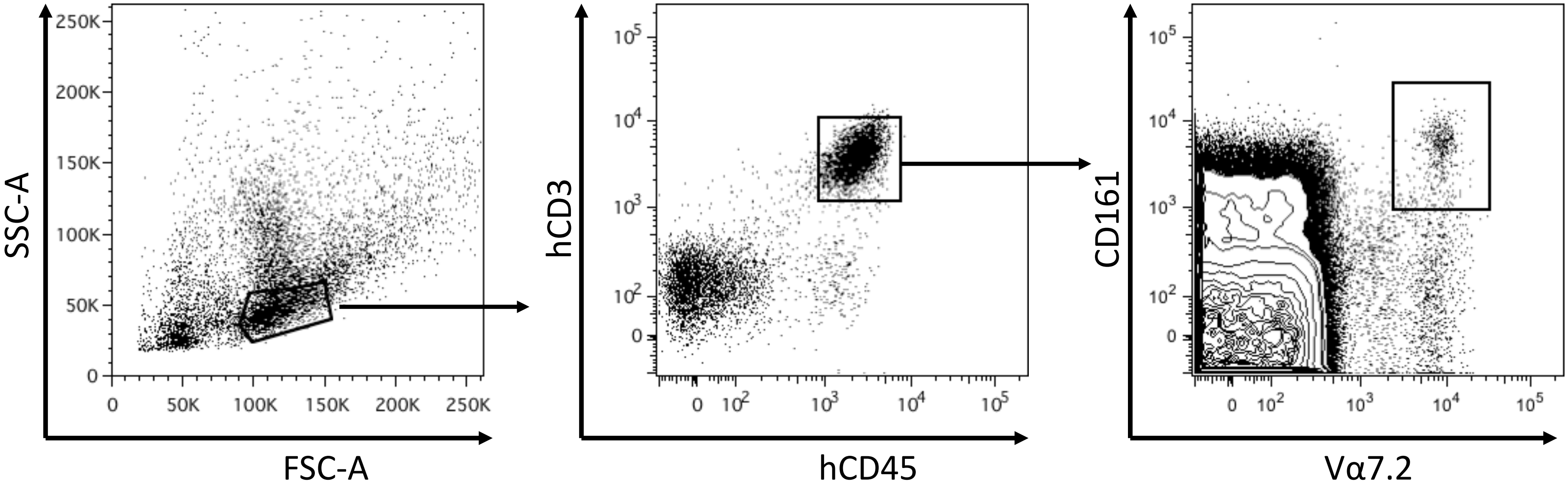

SSC-A
250K
200K
150K
100K
50K
0
0
50K
100K
150K
200K
250K
FSC-A
hCD3
10^5
10^4
10^3
10^2
0
0
10^2
10^3
10^4
10^5
hCD45
CD161
10^5
10^4
10^3
10^2
0
0
10^2
10^3
10^4
10^5
Vα7.2

S1 Fig

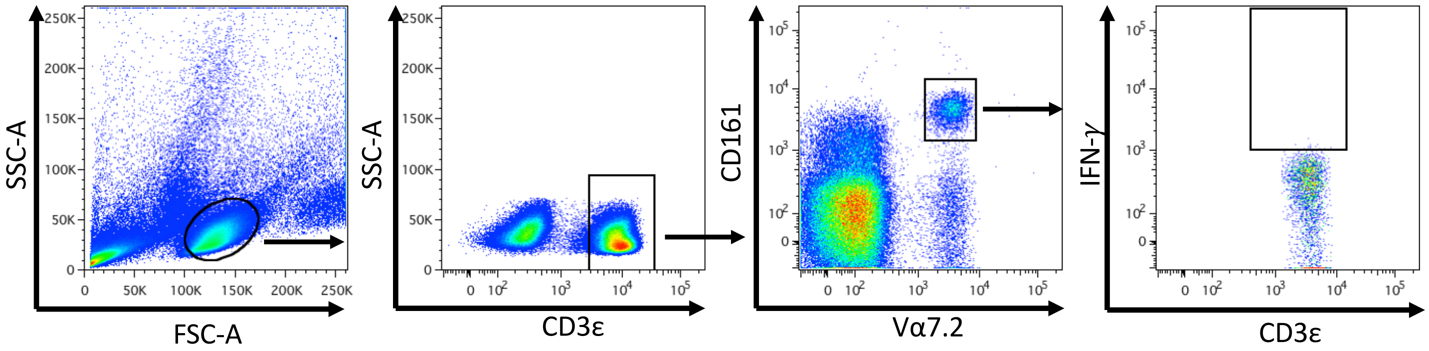

S2 Fig

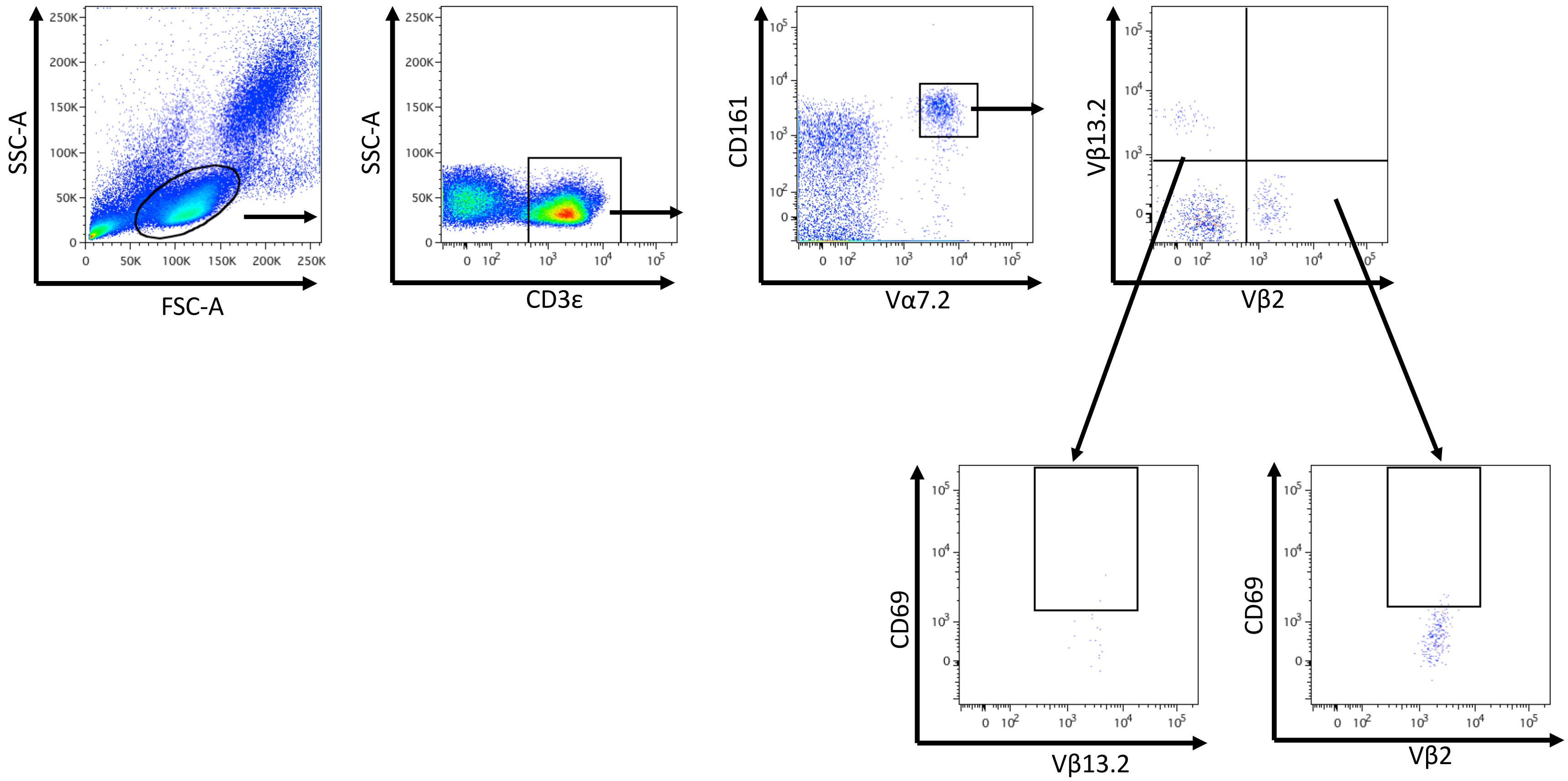

S3 Fig

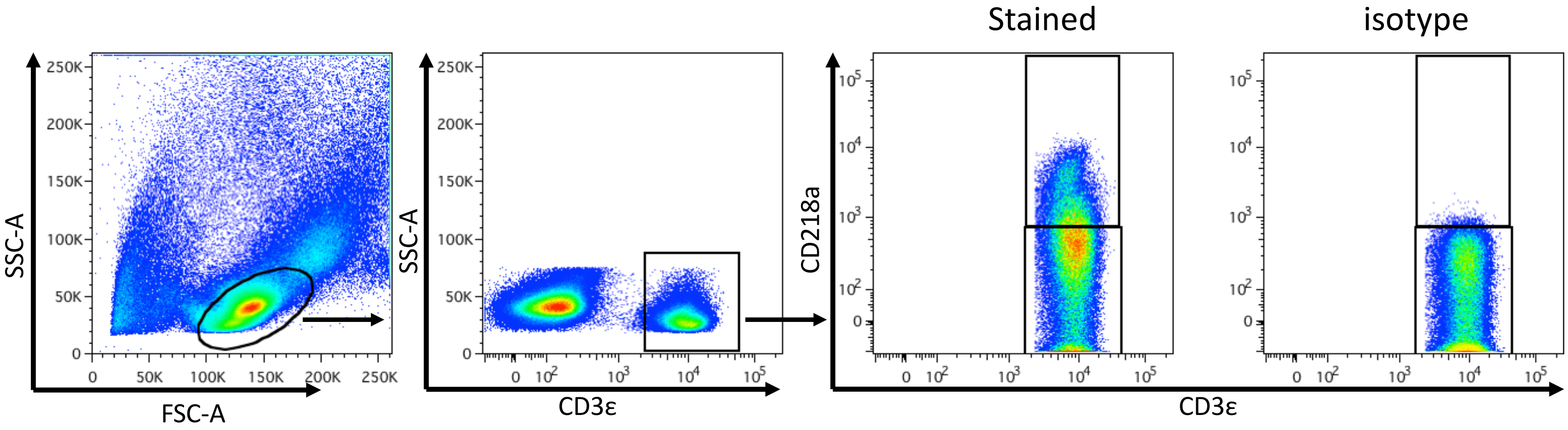

SSC-A
250K
200K
150K
100K
50K
0
FSC-A
50K
100K
150K
200K
CD3ε
CD218a
Stained
isotype

S4 Fig

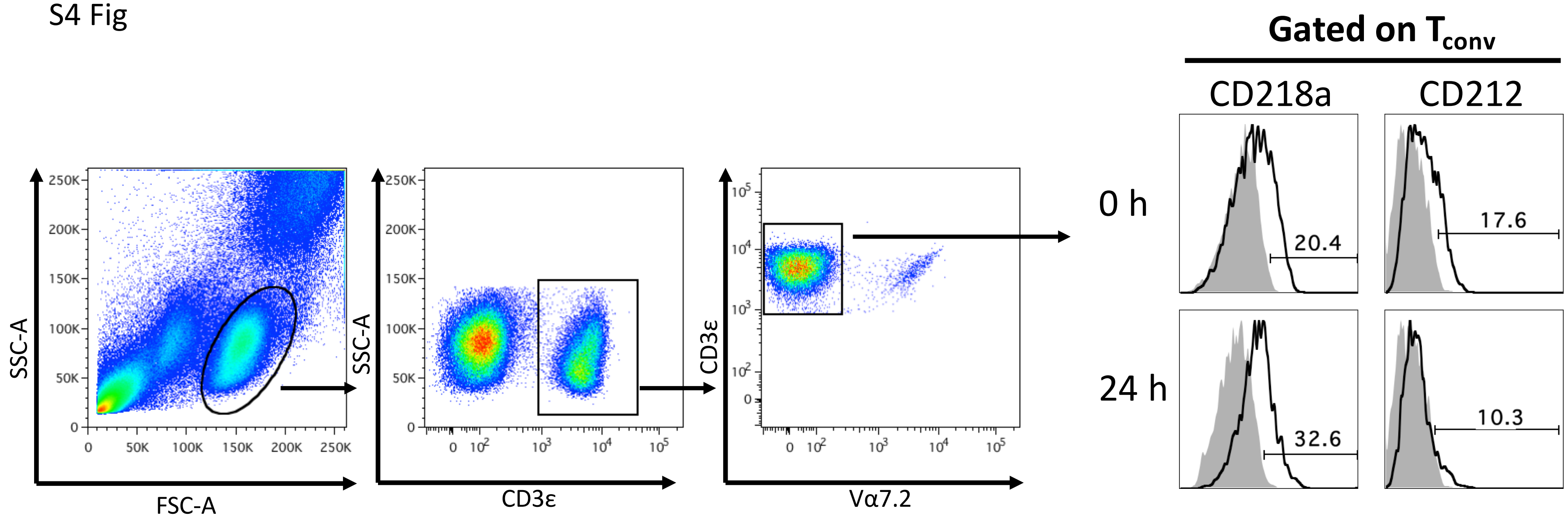

SSC-A
FSC-A
0
50K
100K
150K
200K
250K
CD3ε
Vα7.2
Gated on T_{conv}
CD218a
CD212
0 h
24 h
20.4
17.6
32.6
10.3

S5 Fig & S11 Fig

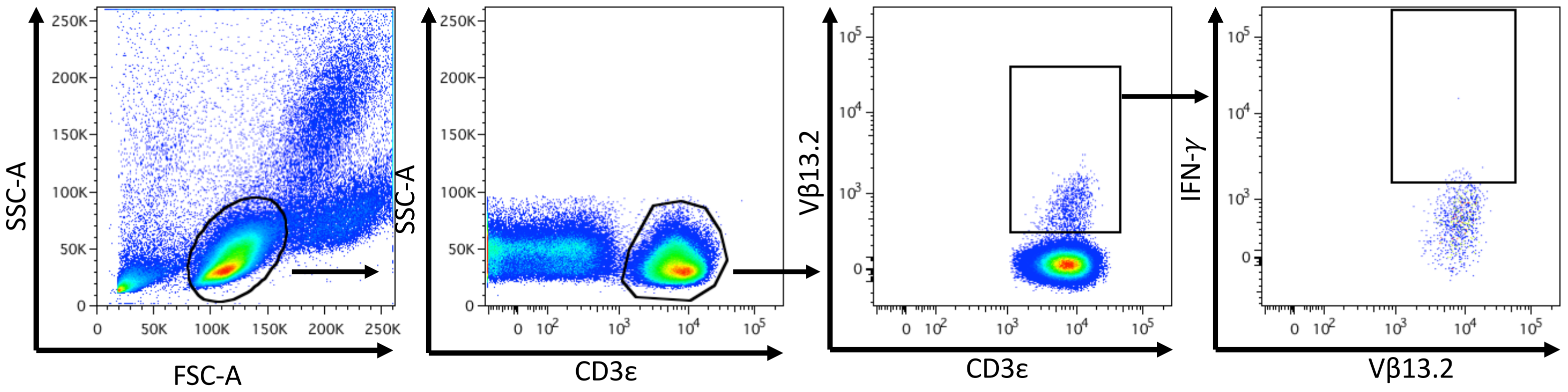

S6 Fig

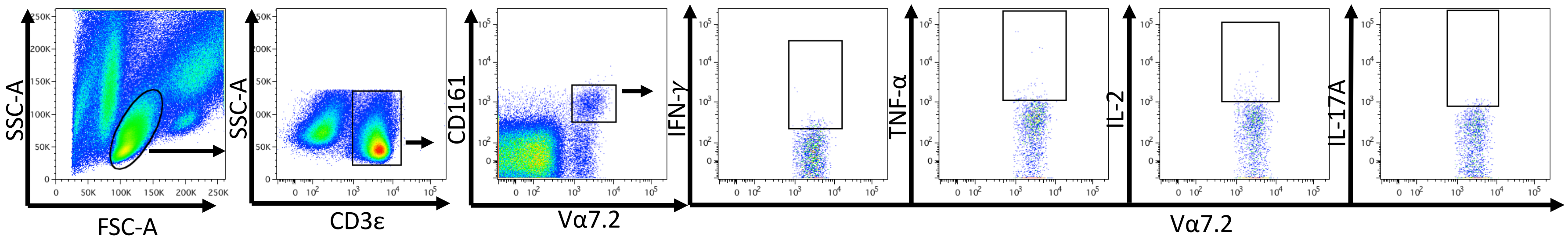

S7 Fig, S8 Fig, S10 Fig

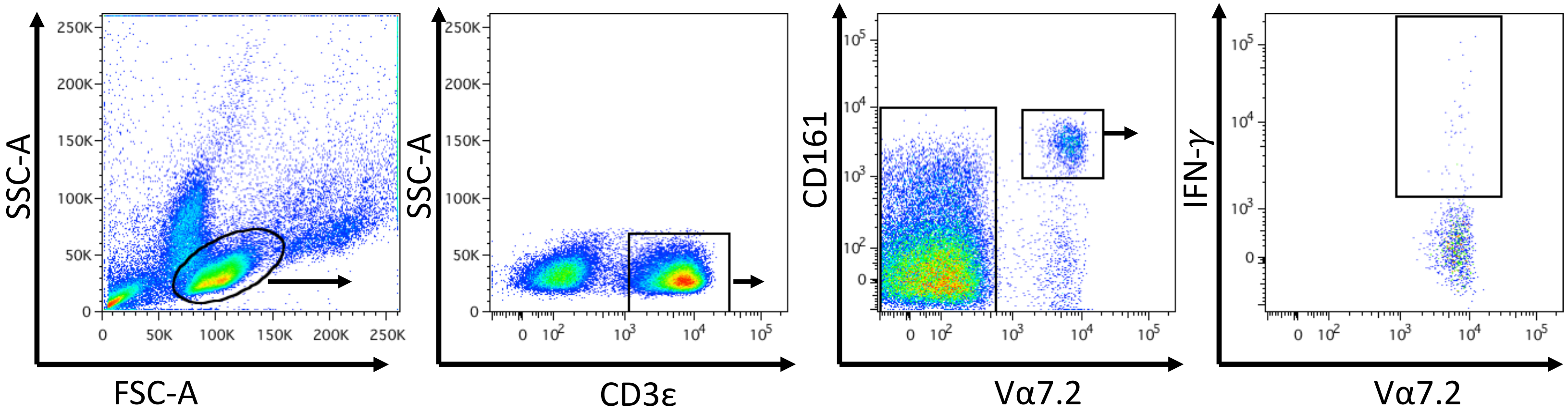

S12 Fig

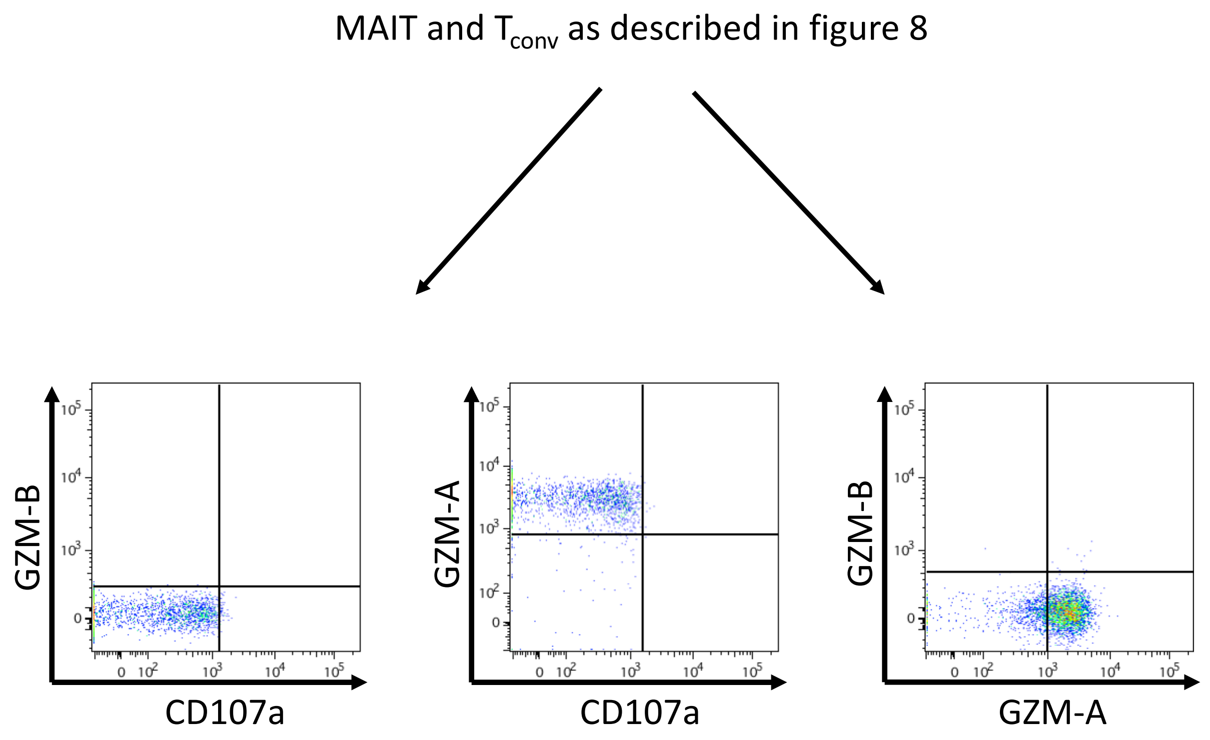
MAIT and T_{conv} as described in figure 8
GZM-B
CD107a
GZM-A
CD107a
GZM-B
GZM-A
