# Supplementary figures and images for: MAIT cells launch a rapid, robust and distinct hyperinflammatory response to bacterial superantigens and quickly acquire an anergic phenotype that impedes their cognate antimicrobial function: Defining a novel mechanism of superantigen-induced immunopathology and immunosuppression
Source: PLoS Biol. 2017 Jun 20;15(6):e2001930. doi: 10.1371/journal.pbio.2001930 (PMC5478099; doi:10.1371/journal.pbio.2001930)

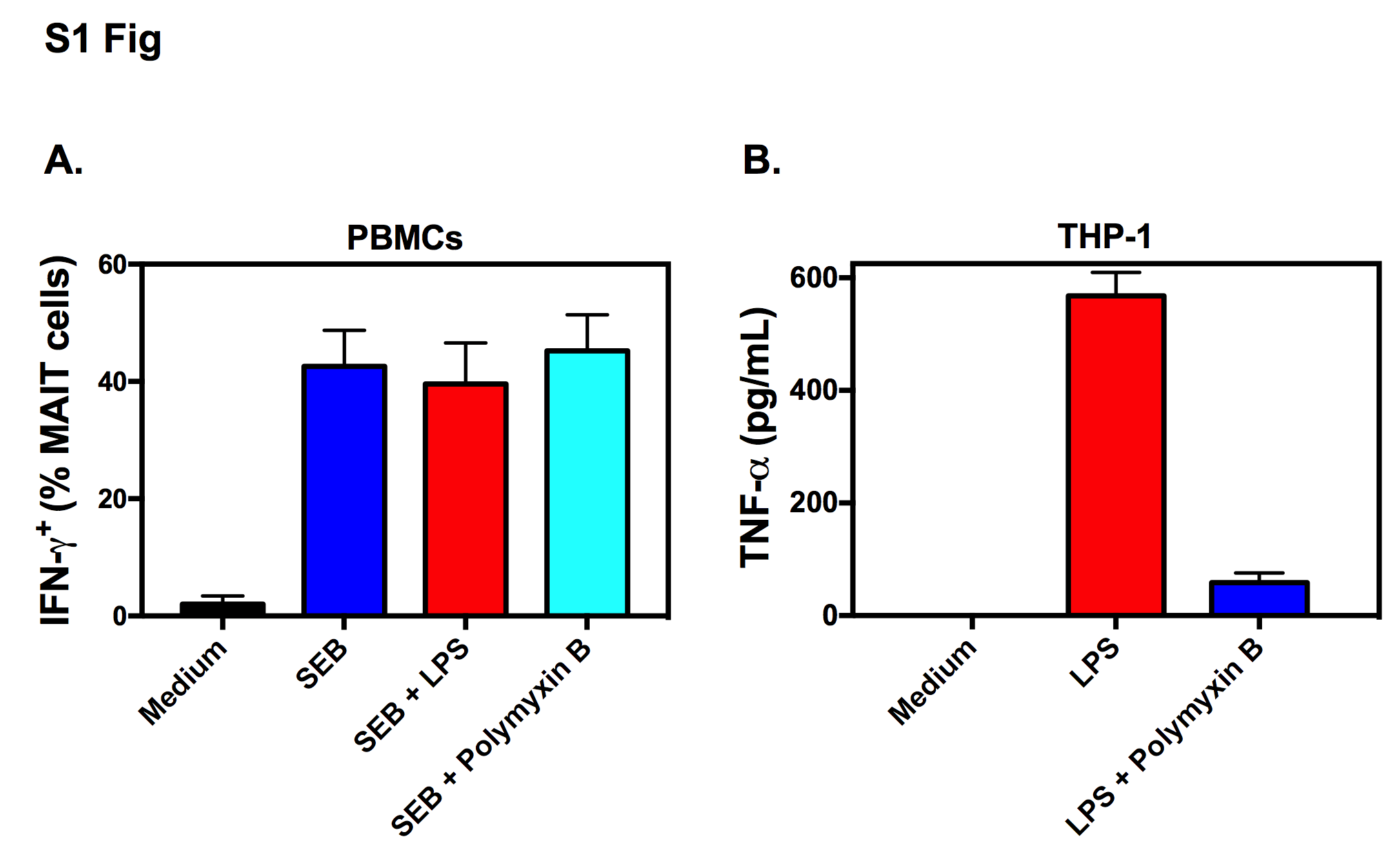

Supplement: S1 Fig — Human PBMCs from 6 healthy donors were left untreated or stimulated with 100 ng/mL of SEB in the presence or absence of 100 ng/mL of LPS or 100 μg/mL of polymyxin B as indicated. Twenty-four h later, the frequency of IFN-γ+ MAIT cells was determined by flow cytometry. Error bars represent SEM (A). THP-1 human monocytic cells were exposed to LPS for 24 h in the presence or absence of 100 μg/mL of polymyxin B, followed, 24 h later, by quantification of TNF-α in culture supernatant samples by ELISA. Error bars represent SD from triplicate culture wells (B). (TIFF) [file pbio.2001930.s004.tiff]

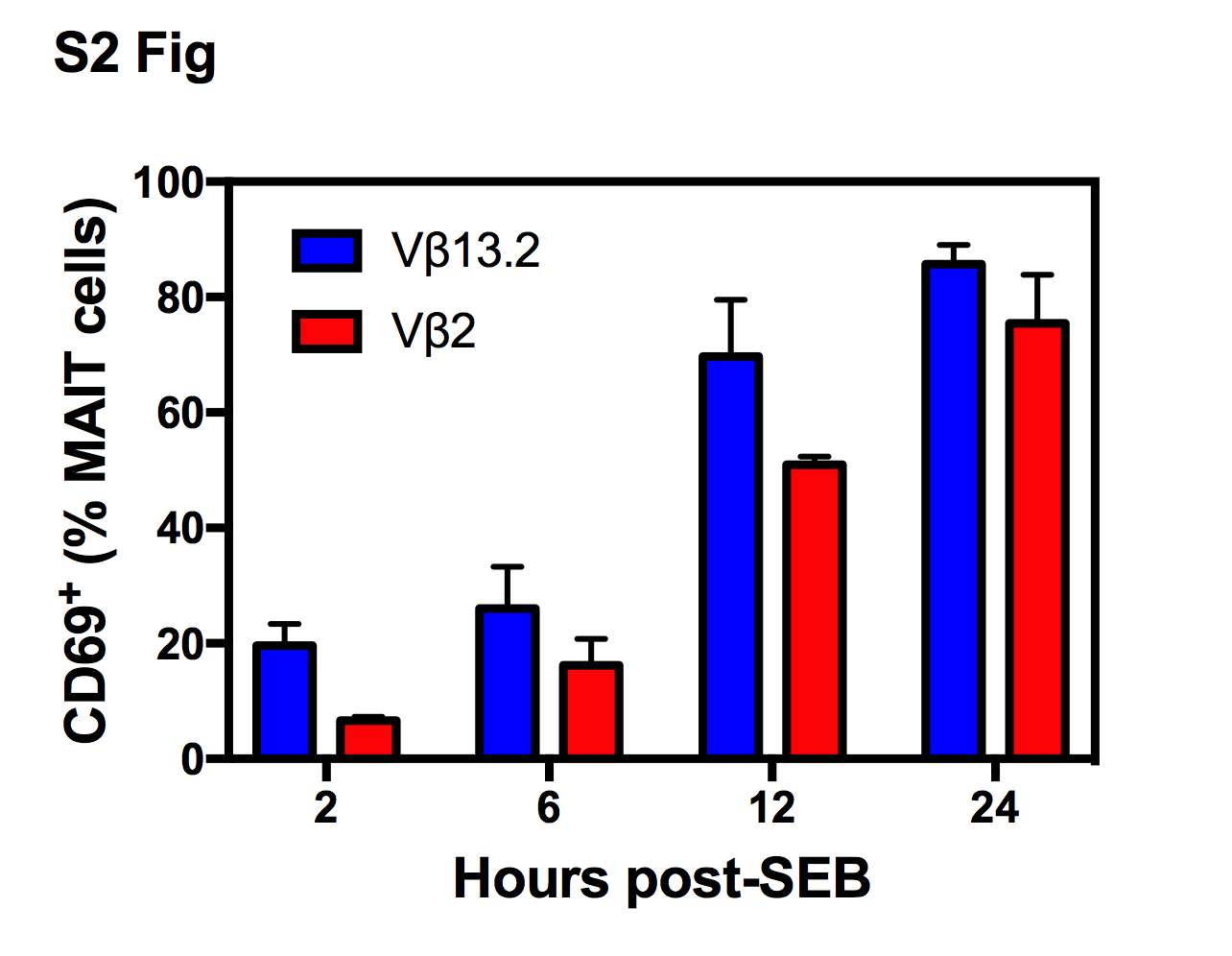

Supplement: S2 Fig — Human PBMCs from 3 donors were exposed to 100 ng/mL of SEB, and the expression of CD69 on TCRVβ13.2+ and TCRVβ2+ MAIT cell subsets was assessed at indicated time points by flow cytometry. Error bars represent SEM. (TIFF) [file pbio.2001930.s005.tiff]

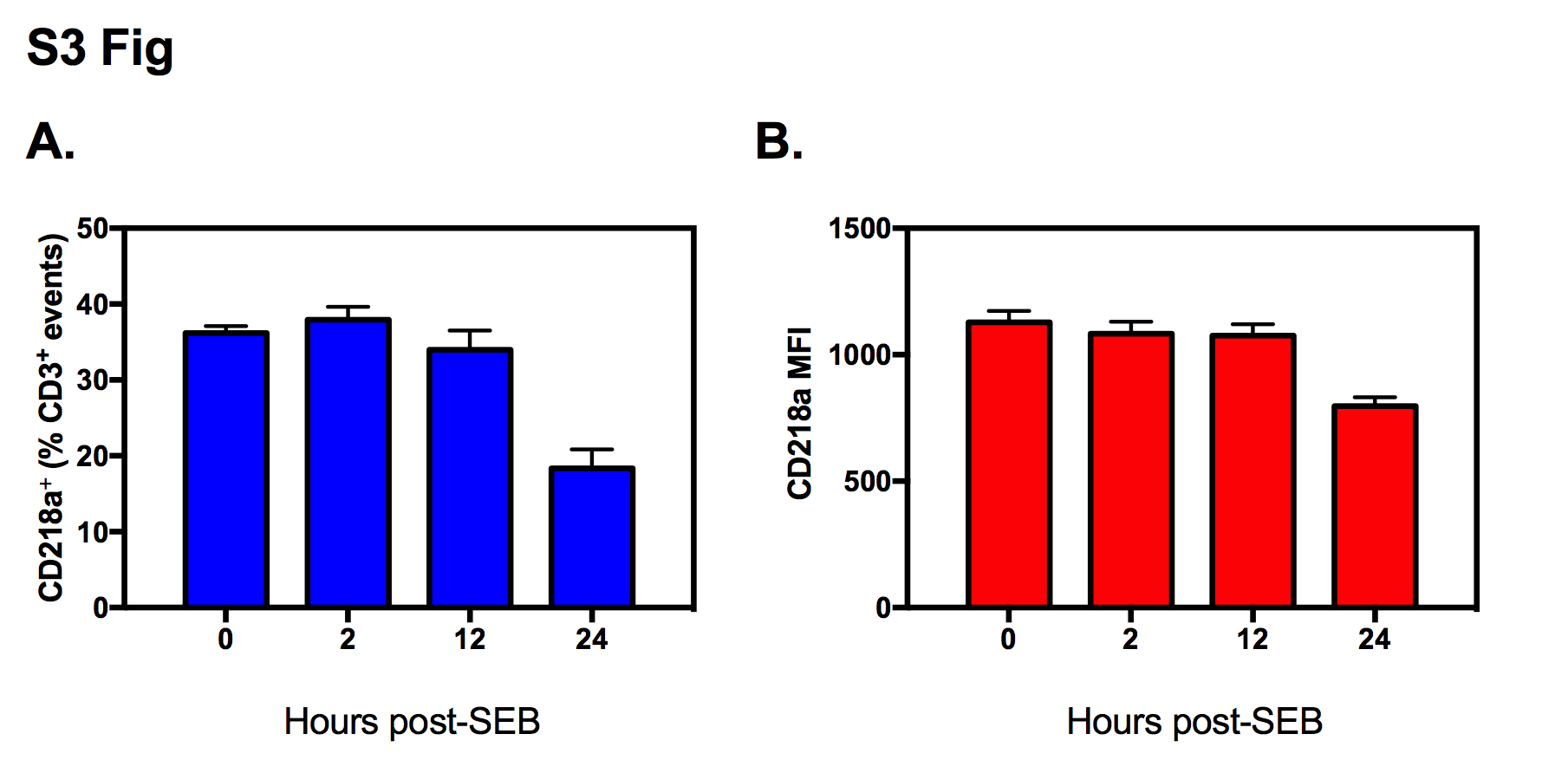

Supplement: S3 Fig — Human PBMCs (n = 3) were left untreated or stimulated with 100 ng/mL of SEB for indicated durations. The percentage of CD218a+ cells among unfractionated T cells (A) and the mean fluorescence intensity (MFI) of CD218a staining (B) were determined by flow cytometry. (TIFF) [file pbio.2001930.s006.tiff]

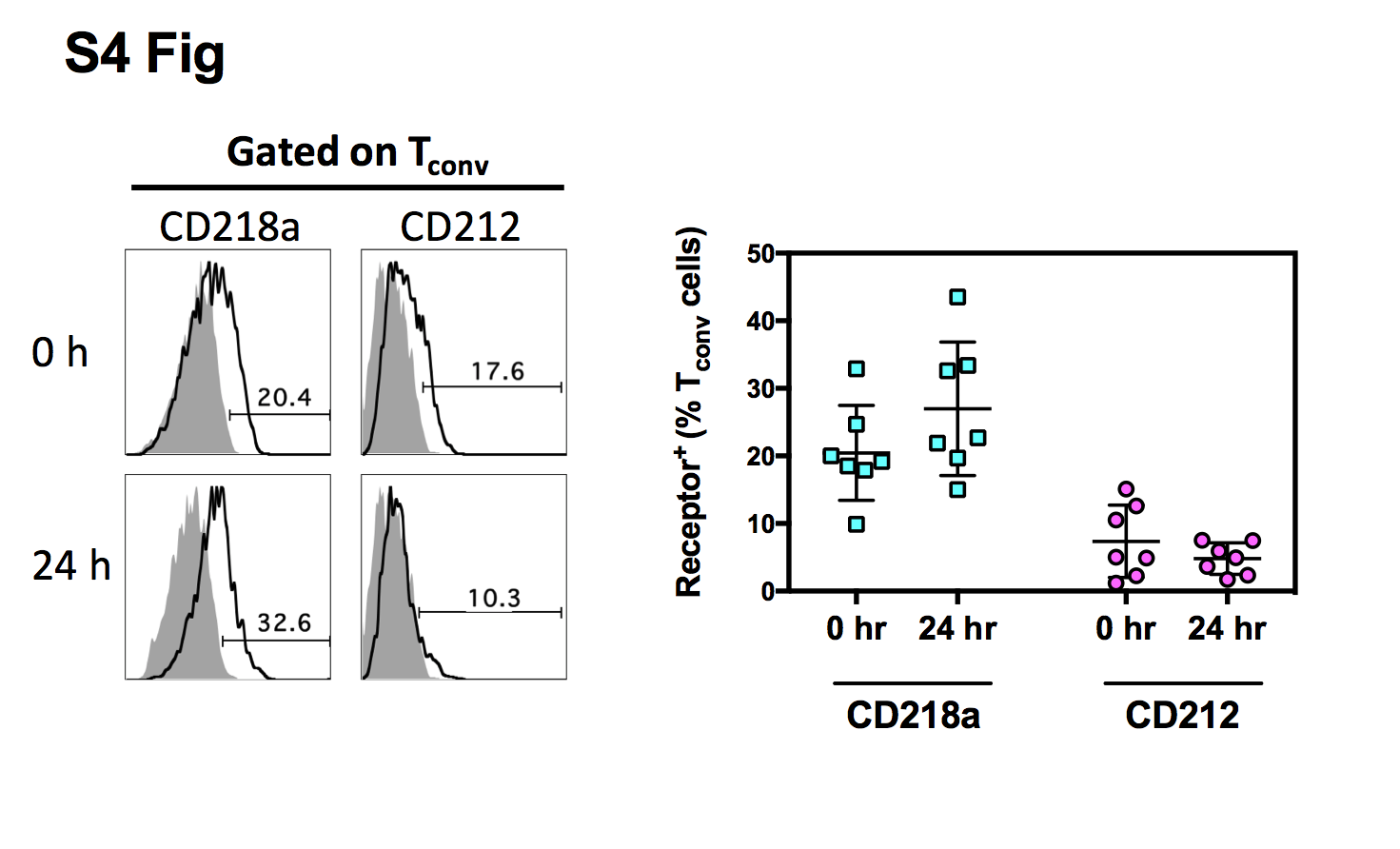

Supplement: S4 Fig — Freshly isolated and SEB-stimulated human PBMCs (n = 7) were analyzed by flow cytometry to determine the frequencies of CD218a+ and CD212+ cells among CD3+Vα7.2- Tconv cells. Filled and open histograms (left panel) correspond to staining with isotype controls and anti-CD218a/CD212, respectively. Each circle represents an individual in the right panel where error bars represent SEM. (TIFF) [file pbio.2001930.s007.tiff]

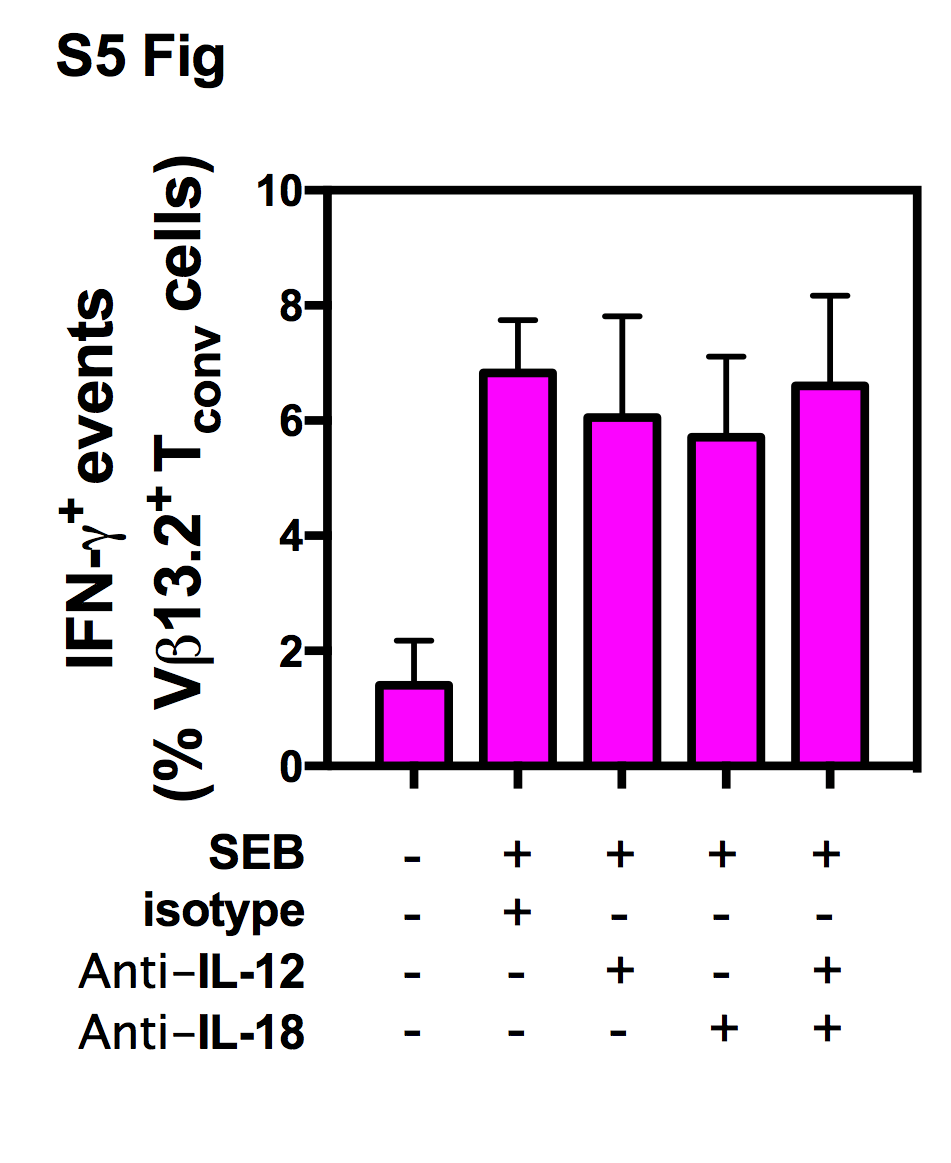

Supplement: S5 Fig — Human PBMCs (n = 6) were stimulated with 100 ng/mL of SEB in the presence of IL-12- and/or IL-18-neutralizing mAbs or an isotype control. Twenty-four h later, the frequency of IFN-γ+ cells among TCRVβ13.2+ Tconv cells was determined by flow cytometry. Error bars represent SEM. (TIFF) [file pbio.2001930.s008.tiff]

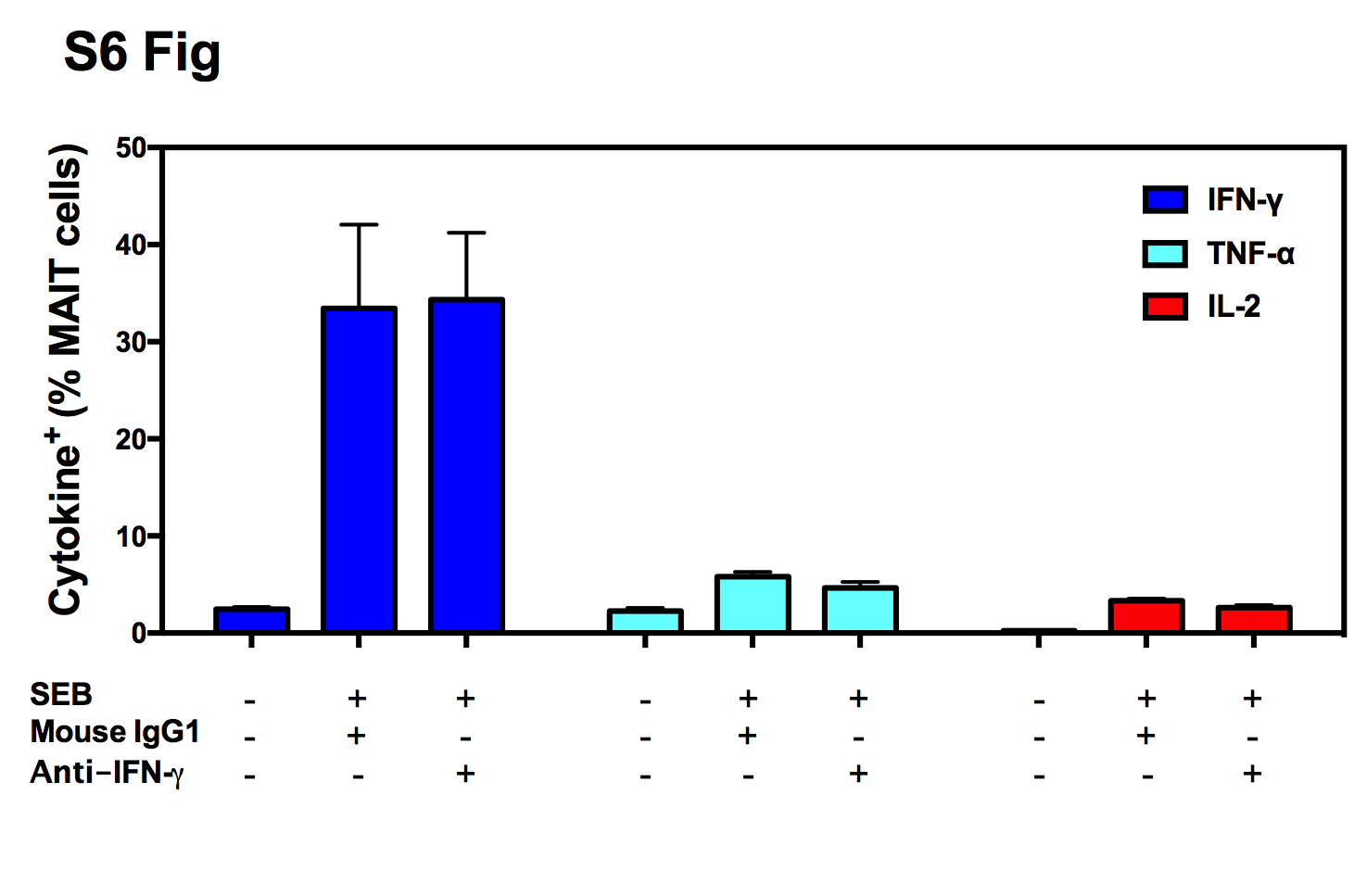

Supplement: S6 Fig — Human PBMCs (n = 4) were stimulated with SEB in the presence of an anti-IFN-γ mAb or isotype control. Twenty-four h later, the frequency of IFN-γ-, TNF-α- and IL-2-producing MAIT cells was determined by flow cytometry. Error bars represent SEM. (TIFF) [file pbio.2001930.s009.tiff]

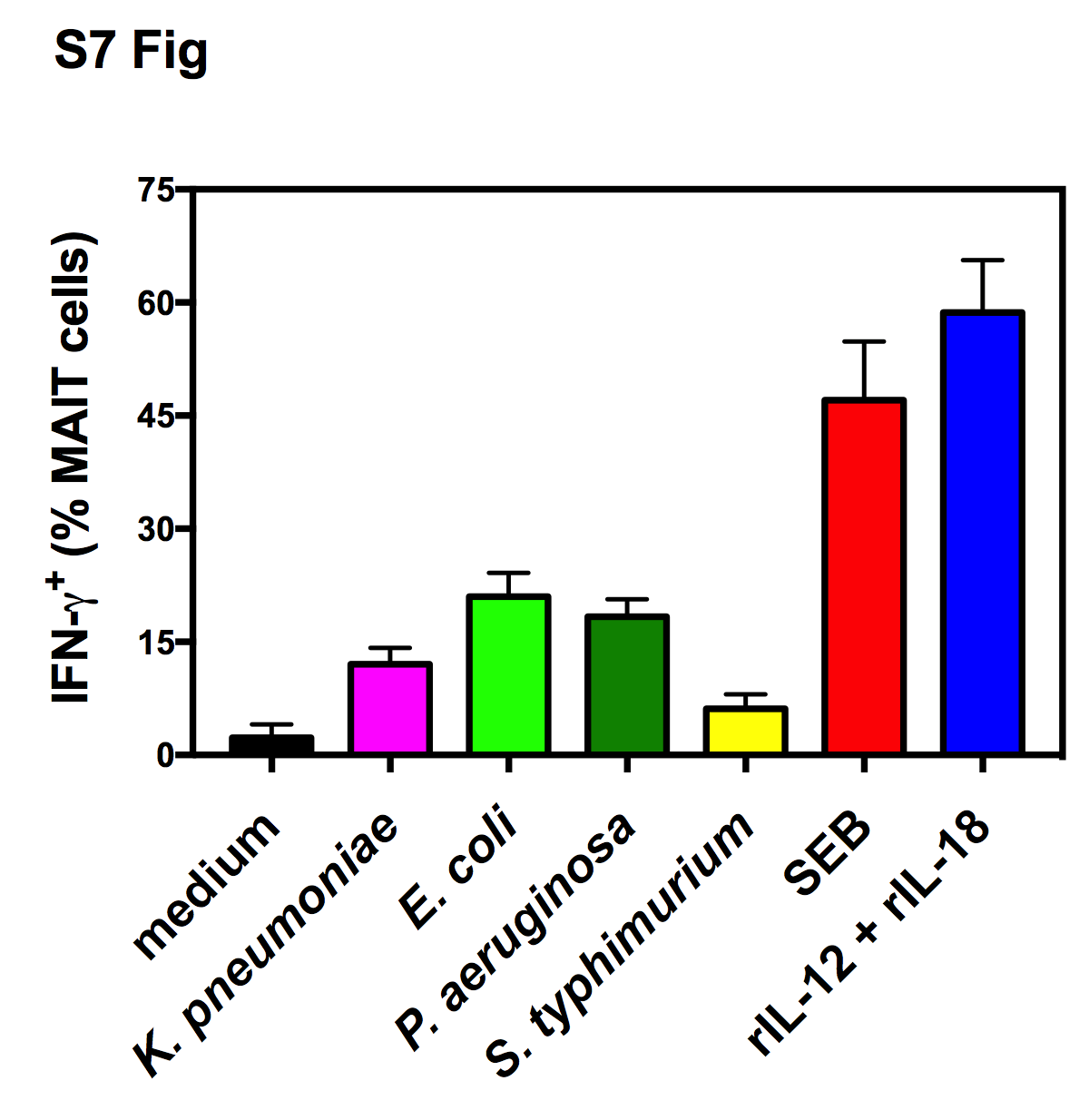

Supplement: S7 Fig — Human PBMCs (n = 7) were left untreated or exposed to SEB, a combination of rIL-12 and rIL-18, or bacterial cell lysates prepared from K. pneumoniae, E. coli, P. aeruginosa or S. typhimurium. Twenty-four h later, the percentage of IFN-γ-producing MAIT cells were calculated. Error bars represent SEM. (TIFF) [file pbio.2001930.s010.tiff]

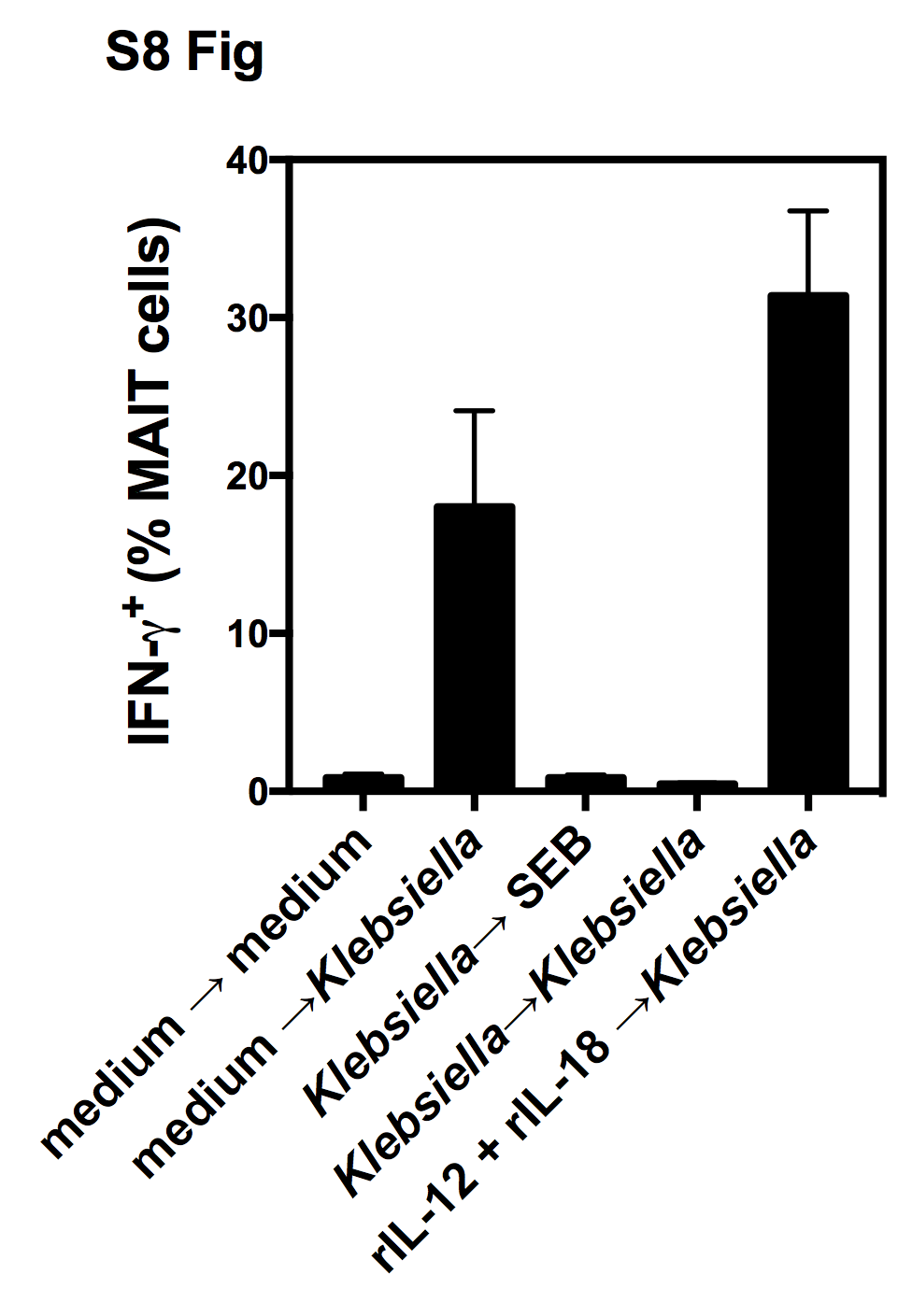

Supplement: S8 Fig — Human PBMCs (n = 13) were left untreated or subjected to stimulation with K. pneumoniae lysate or a combination rIL-12 and rIL-18. Twenty-four h later, cells were washed and rested for an additional 24 h before they were left in complete medium or challenged with SEB or K. pneumoniae lysate as indicated. This was followed, 24 h later, by cytofluorimetric calculation of IFN-γ+ MAIT cell frequencies. Error bars represent SEM. (TIFF) [file pbio.2001930.s011.tiff]

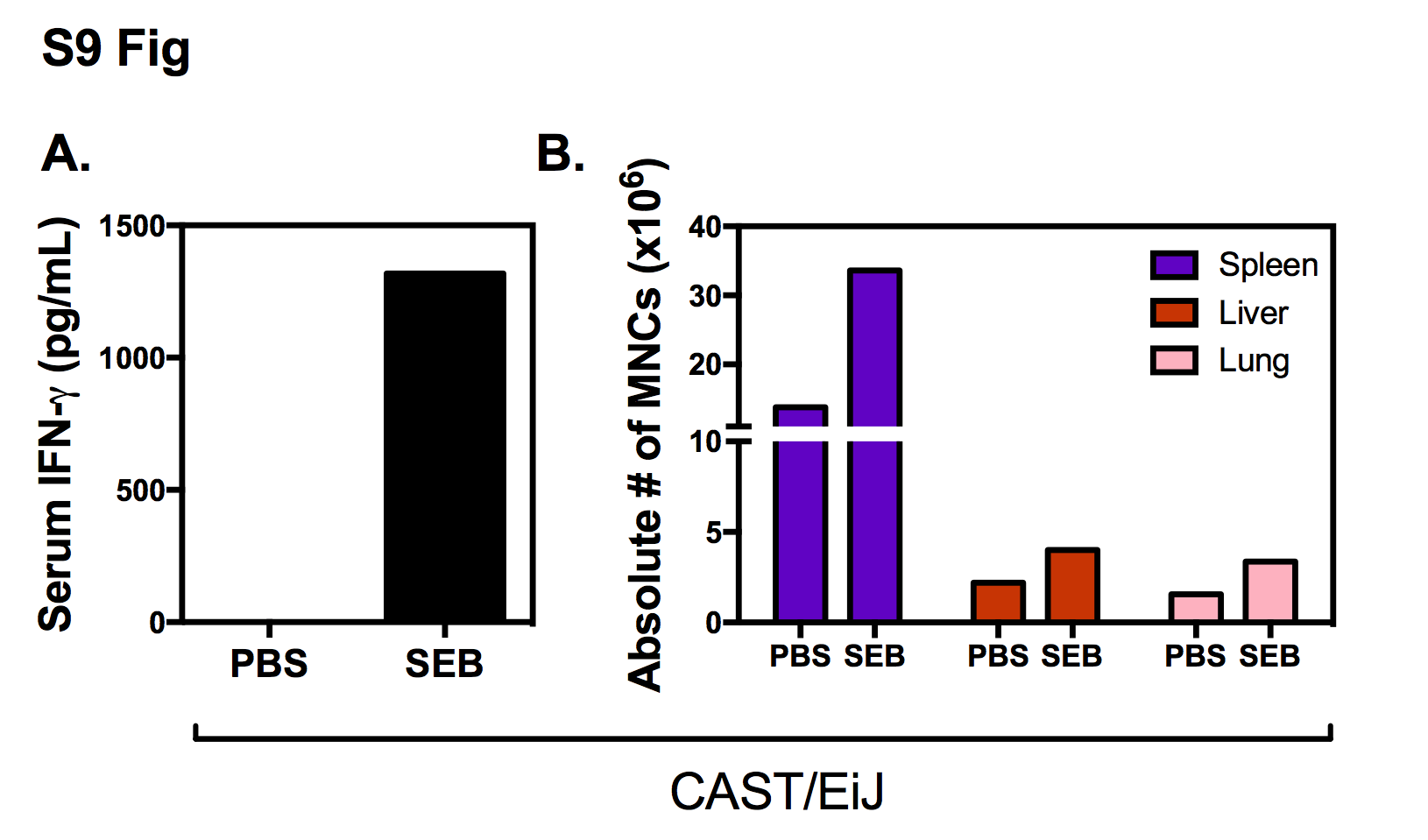

Supplement: S9 Fig — In a pilot experiment, one CAST/EiJ mouse was injected with sterile PBS and another mouse received a 100-μg i.p. injection of SEB. Twelve h later, serum IFN-γ levels were quantitated by ELISA (A). In addition, 4 days after PBS/SEB injection, splenic, hepatic and lung non-parenchymal mononuclear cells (MNCs) were enumerated (B). (TIFF) [file pbio.2001930.s012.tiff]

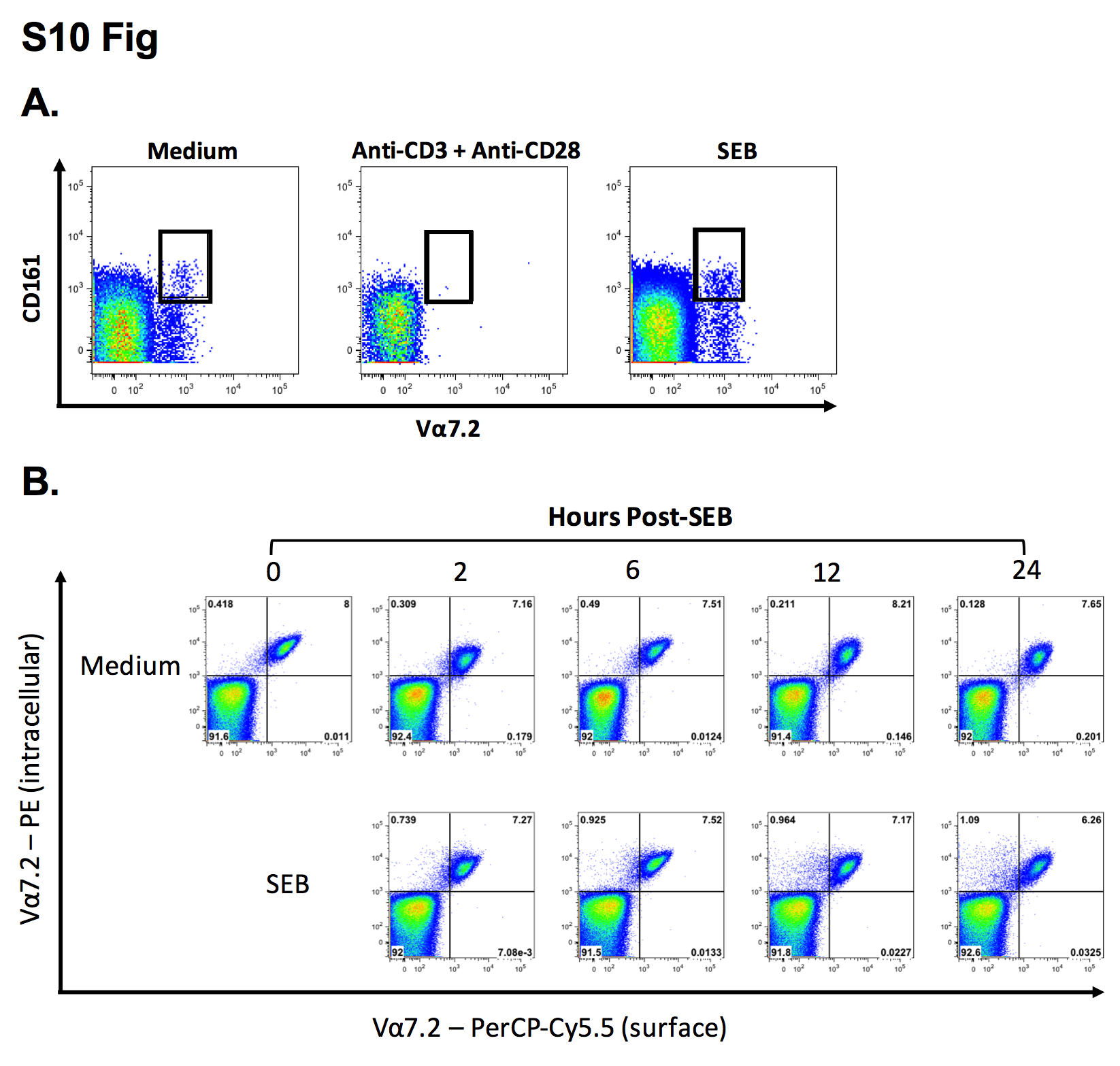

Supplement: S10 Fig — PBMCs (n = 3) were cultured in the absence or presence of SEB (100 ng/mL) or a combination of agonistic anti-human CD3 (clone OKT3) and anti-human CD28 (clone 9.3) mAbs, each of which was used at 0.5 μg/mL. Twenty-four h later, the presence and the frequency, when applicable, of Vα7.2+CD161high MAIT cells were assessed by flow cytometry (A). The extent of iTCR internalization, or lack thereof, was determined at indicated time points through step-wise staining of SEB-exposed cells with Vα7.2 mAbs labeled with two different fluorochromes. Cells were stained for surface iTCR before they were washed, fixed, permeablized and stained for intracellular iTCR. Representative data from one donor are illustrated. (TIFF) [file pbio.2001930.s013.tiff]

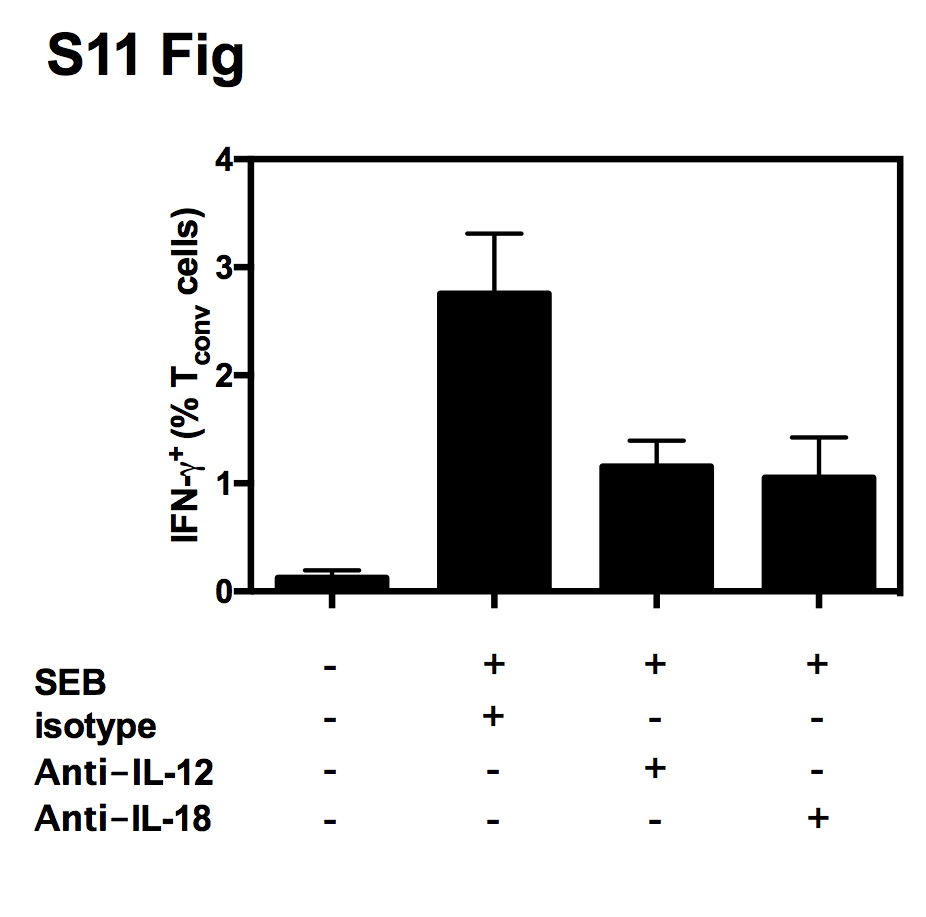

Supplement: S11 Fig — PBMCs (n = 7) were stimulated with SEB in the presence of anti-human IL-12, anti-human IL-18 or isotype control. Twenty-four h later, the frequency of IFN-γ+ Tconv was determined by flow cytometry. Error bars represent SEM. (TIFF) [file pbio.2001930.s014.tiff]

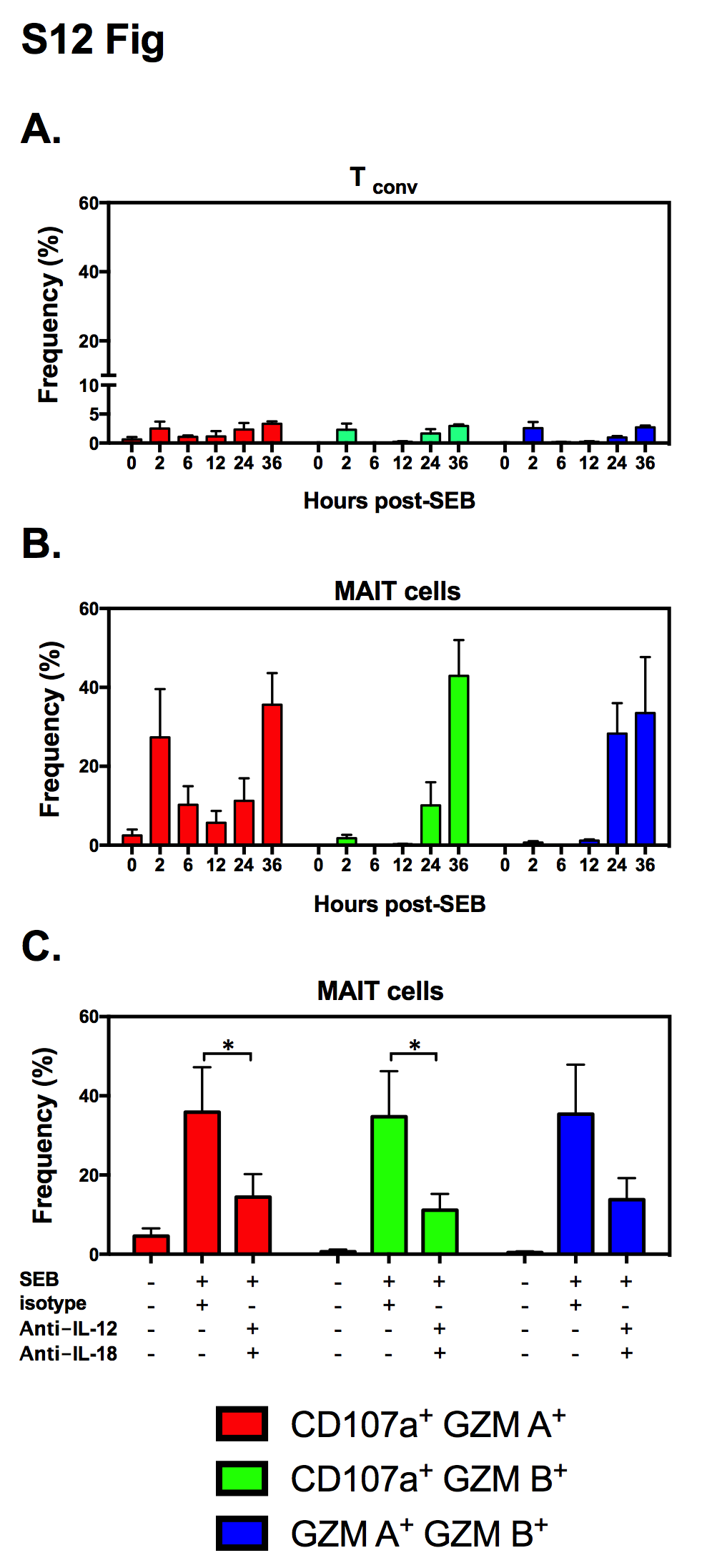

Supplement: S12 Fig — Human PBMCs (n = 7–8) were stimulated with SEB for indicated durations, and the percentages of cells co-expressing CD107a and/or granzymes A/B were determined among Tconv (A) and MAIT cells (B). For five samples, a combination of anti-IL-12 and IL-18 (or isotype control) was present in cultures (C). Error bars represent SEM. (TIFF) [file pbio.2001930.s015.tiff]
